# Earliest filter-feeding pterosaur from the Jurassic of China and ecological evolution of Pterodactyloidea

**DOI:** 10.1098/rsos.160672

**Published:** 2017-02-01

**Authors:** Chang-Fu Zhou, Ke-Qin Gao, Hongyu Yi, Jinzhuang Xue, Quanguo Li, Richard C. Fox

**Affiliations:** 1 Institute of Paleontology, Shenyang Normal University, Shenyang 110034, People's Republic of China; 2 School of Earth and Space Sciences, Peking University, Beijing 100871, People's Republic of China; 3 Institute of Vertebrate Paleontology and Paleoanthropology, Chinese Academy of Sciences, Beijing 100044, People's Republic of China; 4 School of Geosciences, University of Edinburgh, Edinburgh EH9 3JW, UK; 5 State Key Laboratory of Biogeology and Environmental Geology, China University of Geosciences, Beijing 100083, People's Republic of China; 6 Department of Biological Sciences, University of Alberta, Edmonton, Alberta, CanadaT6G 2E9

**Keywords:** Jurassic pterosaurs, feeding adaptations, palaeoecology, Yanliao Biota, western Liaoning of China

## Abstract

Pterosaurs were a unique clade of flying reptiles that were contemporaries of dinosaurs in Mesozoic ecosystems. The Pterodactyloidea as the most species-diverse group of pterosaurs dominated the sky during Cretaceous time, but earlier phases of their evolution remain poorly known. Here, we describe a 160 Ma filter-feeding pterosaur from western Liaoning, China, representing the geologically oldest record of the Ctenochasmatidae, a group of exclusive filter feeders characterized by an elongated snout and numerous fine teeth. The new pterosaur took the lead of a major ecological transition in pterosaur evolution from fish-catching to filter-feeding adaptation, prior to the Tithonian (145–152 Ma) diversification of the Ctenochasmatidae. Our research shows that the rise of ctenochasmatid pterosaurs was followed by the burst of eco-morphological divergence of other pterodactyloid clades, which involved a wide range of feeding adaptations that considerably altered the terrestrial ecosystems of the Cretaceous world.

## 1. Introduction

As a group of fascinating reptiles in Earth history, pterosaurs were the first flying vertebrates, appearing initially in Late Triassic, Norian time (215 Ma; [1–5]), some 65 million years earlier than the first bird (*Archaeopteryx ca* 150 Ma). Since the pioneer discovery in the late 1760s in Germany, over 110 pterosaur species have been named and described worldwide [6–8]. Most fossils have been found at world-famous Lagerstätten such as the Upper Jurassic Solnhofen limestone in Germany, Lower Cretaceous Santana Formation (*sensu stricto*) in Brazil, and more recently from Middle Jurassic through Lower Cretaceous beds in North China. However, the geologically oldest pterosaurs are from Late Triassic (Norian) marine deposits of the Alps and Greenland [5]. During their 149 million year history, from the Late Triassic (215 Ma) to the end of the Cretaceous (66 Ma), the evolution of pterosaurs resulted in a variety of eco-morphological adaptations, as evidenced by differences in skull shape, dentition, body size and body plan (neck length, tail length and wing span) [2–4,9,10]. The most striking differentiation of their feeding adaptations correlated with morphological and phylogenetic diversity took place concurrently with the rise of the Pterodactyloidea, the most species-diverse group of pterosaurs that ruled the sky from Late Jurassic to the end of Cretaceous. The new discovery from China reported here documents the only pre-Tithonian pterodactyloid known with a complete skull, shedding new light on the origin of the Ctenochasmatidae and the timing of the critical transition from fish-catching to filter-feeding, a major ecological shift in the early history of the pterodactyloid clade.

## 2. Material and methods

### 2.1. Age and stratigraphy

**Figure 1.**
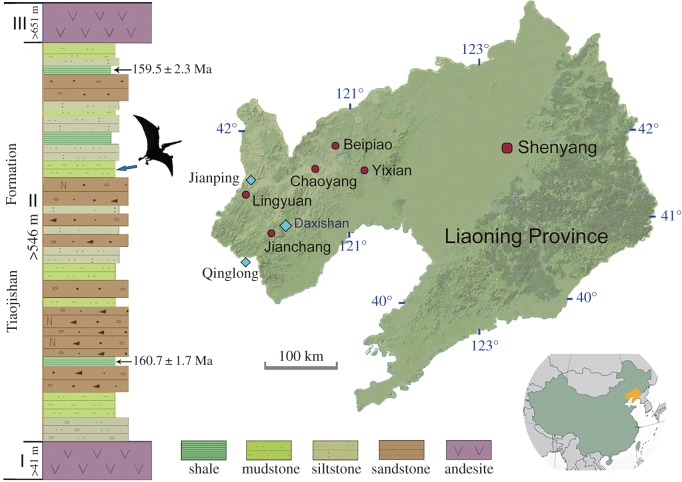
Map and geologic section showing the location of the Daxishan site (40°52′210^″^ N/119° 59′297^″^ E) in relation to the other two major fossil localities (Jianping and Qinglong) of the Tiaojishan Formation in Liaoning Province, northeastern China, and the stratigraphic horizon at which the new pterosaur specimen was collected. Isotopic dates are from references [11,12].

The fossil beds cropping out at the Daxishan locality (figure 1; electronic supplementary material, figure S1) pertain to the Upper Jurassic Tiaojishan Formation, which disconformably overlays the Middle Jurassic Haifanggou (Jiulongshan) Formation, and is beneath the Lower Cretaceous Tuchengzi Formation. The Tiaojishan Formation is composed of 130–970 m thick volcanic rocks, deposited during an interval of *ca* 2.3 Myr from 161.8 ± 0.4 Ma to 159.5 ± 0.6 Ma [11,12]. This formation, along with the underlying Haifanggou Formation, has yielded fossils of the Yanliao Biota, immediately antedating the world-famous Jehol Biota [13]. Recent discoveries of vertebrate fossils from the Tiaojishan Formation include small paravian dinosaurs, mammals and salamanders [14–17]. Pterosaurs recently recovered from the Tiaojishan Formation include rhamphorhynchids, anurognathids and darwinopterans [18–20]. All of these non-pterodactyloids were insectivores or piscivores; no filter-feeding pterosaurs have been previously known from the Tiaojishan Formation nor correlative or earlier beds elsewhere [3,4].

### 2.2. Phylogenetic analysis

There are several published datasets [8,21–23] that can be used in this study. We chose to use the one presented in reference [8] because it provides substantially more extensive sampling of taxa (113 species) and characters (224 characters) than other available datasets. The new taxon was coded in the most recent species-level morphological dataset of all pterosaurs that has been published [8]. Analysis of this dataset (electronic supplementary material, Appendix), using TNT [24,25] with the same settings as in the original publication [8], resulted in three most parsimonious trees, the strict consensus of which (TL = 881.494 steps; CI = 0.354; RI = 0.799) is presented as a calibrated cladogram, enriched with information on species diversity and eco-morphological disparity throughout the history of pterosaur evolution (figure 3*a*,*b*). The new pterosaur is placed as the sister taxon to *Ctenochasma* within the Ctenochasmatidae, and this sister-group relationship is supported by two derived characters: ratio of mandible to skull in length = 0.81; and marginal teeth inclined laterally (see electronic supplementary material).

### 2.3. Statistical methods

Two separate statistical analyses were performed based on a total of 34 morphological characters (electronic supplementary material, table S1) selected from the most recently published dataset [8]. These characters were coded for 89 species that have available information on inferred feeding adaptations (electronic supplementary material, table S2). Metric multidimensional scaling (MDS) analysis was conducted by using the PAST 3.0 software package [26] to provide visualization of the level of similarity/dissimilarity of pterosaur species in terms of feeding-related morphological features. Permutational multivariate analysis of variance (PERMANOVA; [27]) was performed in PAST 3.0 based on Gower distance. Different types of pterosaur feeding adaptations were segregated into 10 categories as *a priori* (electronic supplementary material, table S3). Pairwise comparisons resulted in significant segregation of different feeding adaptations as shown in figure 3*c*,*d* (see electronic supplementary material for details).

## 3. Systematic palaeontology

Pterosauria Kaup, 1834

Pterodactyloidea Plieninger, 1901

Ctenochasmatidae Nopcsa, 1928

*Liaodactylus primus* gen. et sp. nov.

*LSID*. urn:lsid:zoobank.org:act:68CF3BCE-05F5-4EBE-9DBB-F699385EC4D0

*LSID*. urn:lsid:zoobank.org:act:C2917D5A-9AEE-4775-9446-1FC8498E6764

### 3.1. Etymology

Liao, abbreviation for Liaoning; dactylos (Gr.), finger, suffix used for pterosaur names; primus (L.), for the early age of the new pterodactyloid taxon.

### 3.2. Holotype

Palaeontological Museum of Liaoning (PMOL) AP00031, a nearly complete skull, with articulated mandibles and atlas-axis complex.

### 3.3. Type locality and horizon

Fossil site approximately 500 m west of the Daxishan village, Jianchang County, western Liaoning Province, China; Upper Jurassic Tiaojishan Formation.

### 3.4. Diagnosis

Distinguished from all other ctenochasmatid species by having: rostrum approximately 49% of total skull length; nasoantorbital fenestra approximately 31% of skull length; lateral process of pterygoid present, subdividing subtemporal fenestra; mandibular symphysis approximately 30.5% of jaw length; maxillary tooth row extending posteriorly below nasoantorbital fenestra.

### 3.5. Description

**Figure 2.**
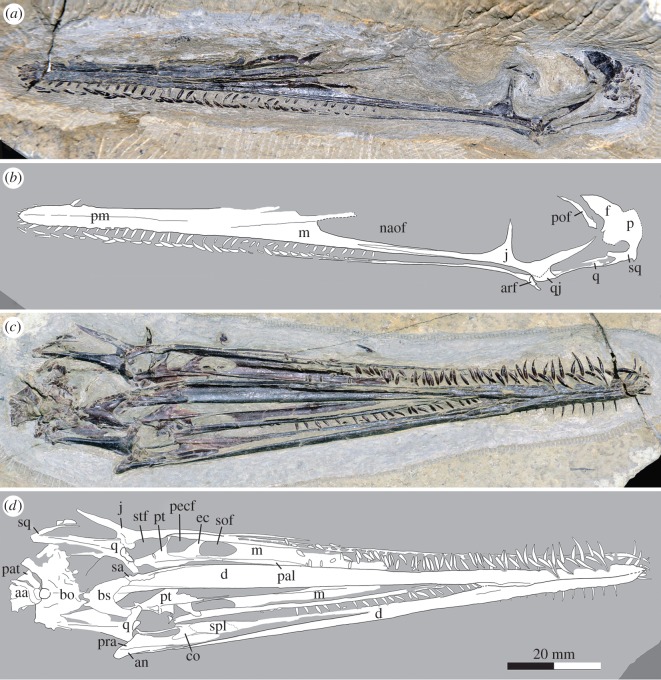
Holotype of *Liaodactylus primus* gen. et sp. nov. (PMOL-AP00031): photographs and line drawings of the nearly complete skull with mandibles in left lateral (*a*,*b*), and palatal (*c*,*d*) views. aa, atlas-axis complex; an, angular; arf, articular facet; bo, basioccipital; bs, basisphenoid; co, coronoid; d, dentary; ec, ectopterygoid; f, frontal; j, jugal; m, maxilla; naof, nasoantorbital fenestra; p, parietal; pal, palatine; pat, proatlas; pecf, pterygo-ectopterygoid fenestra; pm, premaxilla; pof, postfrontal; pra, prearticular; pt, pterygoid; q, quadrate; qj, quadratojugal; sa, surangular; sof, suborbital fenestra; spl, splenial; sq, squamosal; stf, subtemporal fenestra.

**Figure 3.**
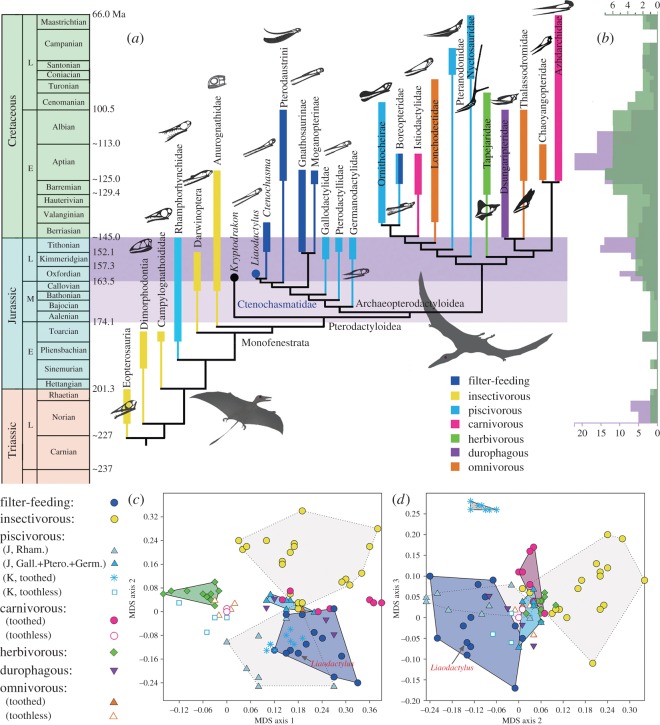
Phylogenetic and eco-morphological diversity of main pterosaur clades. (*a*) Time-calibrated cladogram showing stratigraphic range, eco-morphological diversity of pterosaur clades. Horizontal band in purple highlights Late Jurassic diversification of ctenochasmatids and closely related clades in Archaeopterodactyloidea, antedating Cretaceous diversification of Eupterodactyloidea. Light-purple band indicates hypothesized Middle Jurassic origins of pterodactyloid clades. (*b*) Colour-shaded histogram indicates eco-morphological disparity (green shade denoted by the upper scale) in relation to species diversity (purple shade denoted by the bottom scale) through the evolutionary history of pterosaurs. (*c,d*) MDS ordination plots of pterosaur morphospace (see the electronic supplementary material for details).

The holotype specimen is a nearly complete skull and mandibles, with the first two cervical vertebrae preserved in articulation with the skull (figure 2). The skull is lightly built, 133 mm long, with a slightly concave dorsal profile in lateral view. The external naris is confluent with the antorbital fenestra to form a large nasoantorbital fenestra (figure 2*a*,*b*). This is a diagnostic feature of the Monofenestrata, a major pterosaur clade consisting of the Darwinoptera, Anurognathidae and Pterodactyloidea ([8,18]; figure 3*a*). The rostrum is almost half the length (49.1%) of the skull, a derived feature in the evolution of pterosaurs. The elongation of the rostrum is accompanied by a significant increase in the number of marginal teeth, giving a total of 152 teeth in both sides of the upper and lower jaws of the new pterosaur. The upper tooth row extends posteriorly to below the anterior one-third of the nasoantorbital fenestra. Anterior teeth in both upper and lower jaws are long and needle-like, and lean obliquely laterally (figure 2*c*,*d*; electronic supplementary material, figure S2). Those teeth in the middle and posterior portions of the tooth row gradually decrease in size posteriorly so that the posteriormost teeth are merely short pegs. The teeth are closely spaced to form a ‘comb dentition’, a filter-feeding specialization. The jugal is extremely slender, having a tapering anterior process that extends to near the anterior border of the large nasoantorbital fenestra; the suborbital part of the jugal is narrowed to not much deeper than the anterior process. The quadrate is inclined to a subhorizontal position. The mandibular symphysis is short, nearly one-third of the length of the mandible (electronic supplementary material, figure S3).

## 4. Discussion

The Ctenochasmatidae comprise a diverse group of pterosaurs [8] that were exclusively filter feeders as reflected by their long jaws and characteristic ‘comb dentition’ [2,3]. The family represents a long-ranged clade (Oxfordian–Albian: 160–100 Ma; [28]), and the only pterodactyloid clade that crossed the Jurassic-Cretaceous transition (figure 3*a*). The family contains several well-known forms such as the Late Jurassic *Gnathosaurus* and *Ctenochasma* from Germany, and the Early Cretaceous *Pterodaustro* from Argentina. *Gnathosaurus* has an elongated rostrum (54% of skull length), with 128–136 sharp teeth, and a spatulate and spoon-shaped rostral tip bearing enlarged and projecting teeth. Such jaw structure and tooth morphology would have allowed *Gnathosaurus* to sieve for crustaceans or other small marine organisms [2–4]. In comparison, *Ctenochasma* has a more elongated rostrum (64% of skull length) and as many as 200–552 needle-like fine teeth suitable for filtering crustaceans, tiny molluscs or insect larvae [29,30]. *Pterodaustro*, popularly called the ‘flamingo pterosaur’, represents the most remarkable filter-feeding pterosaur known from the fossil record. It has a greatly elongated (85% of skull length) and strongly upwardly curved rostrum, a huge number (more than 1000) of densely spaced ‘teeth’ (elastic bristles) in its lower jaws, for filtering small crustaceans, microscopic plankton or algae from open water along lake shores [2–4,31]. Several other forms (*Gegepterus*, *Feilongus* and *Moganopterus*) from the Early Cretaceous Yixian Formation (120–129 Ma) of China are also members of the Ctenochasmatidae [8], as they are also characterized by elongate jaws bearing a varied number of needle-like teeth (see the electronic supplementary material). These Early Cretaceous forms, however, have a more elongate rostrum (67–69% of skull length) than the new pterosaur described here.

The new pterosaur *Liaodactylus* is the oldest known ctenochasmatid, predating the previously Tithonian (152 Ma) record (*Gnathosaurus* and *Ctenochasma* from Germany) by at least 8–10 Myr [28]. A purported ctenochasmatid from the Middle Jurassic of the United Kingdom (cf. *Gnathosaurus* sp.) has been shown to be a stem crocodilian [4,8]. Moreover, *Liaodactylus* is the first Oxfordian pterodactyloid to be documented by cranial remains: the most basal pterodactyloid *Kryptodrakon*, an approximate contemporary of *Liaodactylus*, is represented only by an incomplete skeleton lacking the skull from the Upper Jurassic Shishugou Formation, Xinjiang, China [8,32].

Pterosaurs display an extraordinary eco-morphological disparity in feeding adaptations, expressed in skull, jaws and dentition. Previous authors have identified several feeding categories, including: insectivorous, piscivorous, filter-feeding and several other feeding adaptations in different groups of pterosaurs [2–4,33–41]. Plotting this information on to the phylogenetic framework resulting from this study, we have integrated for the first time eco-morphological disparity in feeding adaptations across the phylogenetic framework among pterosaur clades (figure 3*a*). In this study, recognition of these different eco-morphological types by previous authors is supported by our statistical analysis, in which morphospace patterns are in congruence with partitioning of feeding adaptations (figure 3*c*,*d*). Of the non-pterodactyloid pterosaurs, Late Triassic Eopterosauria (eight species), the basalmost pterosaur clade, were mainly insectivorous [2,5,34], as inferred from their lightly built skull and heterodont teeth with multicuspid crowns. Jurassic insectivores include the Dimorphodontia (two species), Campylognathoididae (two species) and Darwinoptera (five species), whereas the Anurognathidae (four species) were the only Jurassic insectivores that survived the Jurussic–Cretaceous transition, but became extinct in the Early Cretaceous Aptian (*ca* 122 Ma). All of these generally have a lightly built skull with sharply pointed teeth, although different families are distinguished from one another by other morphological features. Jurassic Rhamphorhynchidae (nine species) were exclusively fish-catchers, as evidenced by their enlarged, widely spaced and procumbent teeth, and by stomach contents in some specimens [2–4,34,35]. Although a fairly large number of taxa (17 species and six families) have been documented by fossils from Late Triassic to Late Jurassic (215–160 Ma), the first 55 million years of pterosaur evolution were marked with low eco-morphological diversity, as the pterosaurs during this time period were predominantly insectivorous or piscivorous.

The rise of the ctenochasmatid clade signifies the first major ecological shift in pterosaur evolution from insectivorous/piscivorous to filter-feeding, followed by a second and more dynamic transition in Cretaceous time as evidenced by the surge of species diversity and eco-morphological differentiation of pterodactyloid clades (figure 3*a*,*b*). However, primitive pterodactyloids (Archaeopterodactyloidea) also display relatively low eco-morphological diversity. Besides the filter-feeding Ctenochasmatidae, other families (Gallodactylidae, Pterodactylidae and Germanodactylidae) of this group from the Late Jurassic of Germany and the UK were essentially fish-eaters, although their diet might also have included invertebrates ([2–4]; see the electronic supplementary material).

The most striking eco-morphological differentiation in pterosaur evolution occurred within the Eupterodactyloidea, a group of advanced pterodactyloids (figure 3*a*). During Cretaceous time, at least 55 species in 11 families within the Eupterodactyloidea had engaged in a variety of feeding adaptations, including filter-feeding, fish-eating, carnivory and scavenging, herbivory including frugivory, mollusc shell-crushing (durophagy) and omnivory. Except for the filter-feeding and fish-eating boreopterids, other eupterodactyloid clades display essentially unified feeding habit within each family. The Early Cretaceous istiodactylids with razor-edged and interlocked teeth have been regarded as predatory carnivores; whereas, the Late Cretaceous azhdarchids with a giraffe-sized body and enormous but edentulous jaws have been hypothesized as foragers feeding on small animals and carrion in diverse terrestrial environments [4,36–38]. The Early Cretaceous tapejarids may have been herbivorous, as inferred from their short, high-profile skull with a prominent dorsal crest and parrot-like beak[3,4,39–41]. The Early Cretaceous dsungaripterids were most likely mollusc-shell crushers, as evidenced by their tweezer-like beak and robust crushing dentition [2–4]. Fish-catching eupterodactyloids involved four families: ornithocheirids and boreopterids were typical fish-catchers with long jaws, large and loosely spaced teeth; whereas, the nyctosaurids and pteranodontids with a large skull but tapering and toothless jaws were suitable for seizing fish in open-water environments [33,34]. In addition, several families (Lonchodectidae, Thalassodromidae and Chaoyangopteridae) were probably omnivorous, as they lack skull and tooth features for a specific type of dietary preference [3,4].

To sum up, the new fossil discovery reported in this paper documents geologically the earliest record of filter-feeding pterosaurs, marking the initial step of eco-morphological diversification of pterodactyloids from insectivorous/piscivorous to a variety of feeding adaptations. Combining our phylogenetic results with morphometric analysis reveals patterns of pterosaur evolution: the first 55 Ma (Late Triassic–Late Jurassic) of pterosaur evolution were characterized by low eco-morphological diversity with only insectivores/piscivores involved. The rise of the Ctenochasmatidae in the Late Jurassic marked a major ecological shift within the pterodactyloid clade. This ecological transition is followed by the burst of species diversity and eco-morphological differentiation of pterodactyloids, which significantly altered the terrestrial ecosystems of the Cretaceous world.

## Supplementary Material

Supplementary Information

## Data accessibility

All supplementary information (SI) has been uploaded as the electronic supplementary material.
