# Supplementary material for: Earliest filter-feeding pterosaur from the Jurassic of China and ecological evolution of Pterodactyloidea
Source: R Soc Open Sci. 2017 Feb 1;4(2):160672. doi: 10.1098/rsos.160672 (PMC5367317; doi:10.1098/rsos.160672)
Supplement: Supplementary Information [file rsos160672supp1.docx]

**Supplementary Information**

Earliest filter-feeding pterosaur from the Jurassic of China and ecological evolution of Pterodactyloidea

Chang-Fu Zhou^1^, Ke-Qin Gao^2^, Hongyu Yi^3,4^, Jinzhuang Xue^2^, Quanguo Li^5^ & Richard C. Fox^6^

^1^Institute of Paleontology, Shenyang Normal University, Shenyang 110034, China

^2^School of Earth and Space Sciences, Peking University, Beijing 100871, China

^3^Institute of Vertebrate Paleontology and Paleoanthropology, Chinese Academy of Sciences, Beijing 100044, China

4School of Geosciences, University of Edinburgh, Edinburgh EH9 3JW, United Kingdom

^5^State Key Laboratory of Biogeology and Environmental Geology, China University of Geosciences, Beijing 100083, China

^6^Department of Biological Sciences, University of Alberta, Edmonton, AB T6G 2E9, Canada

Correspondence and requests for materials should be addressed to K.-Q.G. (email: [kqgao@pku.edu.cn](mailto:kqgao@pku.edu.cn)).

**Content of Supplementary Information:**

Material and Methods

Phylogenetic Diversity and Eco-morphological Disparity

Morphospace of Pterosaur Feeding Adaptations

Geological Setting

Anatomical Description of the Holotype (PMOL-AP00031)

References

Supplementary figures S1 to S3

Supplementary tables S1 to S3

Appendix. Taxon/Character Data Matrix for Phylogenetic Analysis

**Material and Methods**

The holotype and the only known specimen (PMOL-AP00031) of the new pterosaur (*Liaodactylus* *primus* gen. et sp. nov.) consists of a nearly complete skull with articulated mandibles and atlas-axis complex. The specimen was originally exposed in palatal view, but has been manually prepared to reveal the dorsolateral aspect of the skull as shown in our figure 2. To reveal jaw structures, the specimen was CT-scanned using a High-Resolution X-ray CT scanner (Nikon XT H 320 LC) at the China University of Geosciences (Beijing). The scanning (220 kV, 445 μA) produced 1999 slices of CT images. Three-dimensional reconstruction of the snout and jaws (figure S2) was completed using VGStudio MAX 2.1.

Phylogenetic analysis was performed to resolve the relationships of the new pterosaur within the Pterodactyloidea. We adopted a recent morphological data matrix from reference [8]. With incorporation of the new taxon, the revised data matrix includes 224 characters coded for 113 taxa (SI Appendix). A total of 80 characters was scored for the new taxon; the remaining characters were coded as unknown (18 characters were treated as inapplicable), because the new specimen preserves no postcranial skeleton except the atlas-axis complex. As in the original data matrix, we used a combination of discrete numerical characters and continuous characters with a range of values scaled between 0 and 1 [42].

We conducted a parsimony analysis using TNT [24,25]. Search parameters are the same as in reference [8]: ratchet with 2000 replications, ambiguous branch support abandoned, zero-length branches collapsed automatically, and resultant trees filtered for the best score. The matrix and search parameters are available as an executable TNT file (SI Appendix). Our analysis resulted in three most parsimonious trees (MPTs), the strict consensus of which has a tree length of 881.494 steps, a consistency index of 0.354, and a retention index of 0.799. Incorporation of the new pterosaur taxon caused no change of the overall tree topology in comparison with the single MPT in reference [8]. The new pterosaur was grouped with *Ctenochasma* as sister taxa within the Ctenochasmatidae. The *Liaodactylus*-*Ctenochasma* sister-group relationship is supported by two synapomorphies ([character]-[character state]): ratio of mandible to skull in length= 0.81 ([2]-[0.37]); and marginal teeth inclined laterally ([141]-[1]). The new taxon shares with other ctenochasmatids the following derived cranial character states: skull length maximum at squamosal relative to skull height maximum at jaw articulation is range from 9.949 to 10.774 ([0]-[0.769–0.846]); rostrum length at external naris relative to skull length at squamosal is 0.50 ([3]-[0.555]); retroarticular process on mandible pointing posteroventrally ([130]-[0]).

The consensus tree is shown as a time-calibrated cladogram with species and generic-level taxa merged into generic or family-level clades (figure 3). The cladogram was further enhanced with information on species diversity and eco-morphological disparity throughout the history of pterosaur evolution (see below). Generic and family-level clades were color-coded to show eco-morphological evolution of pterosaurs from the Late Triassic (Norian) time through the Late Cretaceous (Maastrichtian) time. Ecology of *Kryptodrakon* is coded as unknown, because the fossil material lacks the skull and dentition.

**Phylogenetic Diversity and Eco-morphological Disparity**

The pterosaur skull and dentition display diverse anatomical features reflecting a variety of eco-morphotypes related to their distinctive feeding adaptations. Previous studies [2–4] have discussed different types of feeding adaptations, with relevant information involving a total of 108 species in 20 family groups. Eco-morphotypes recognized so far include: insectivorous, piscivorous, filter-feeding, carnivorous, durophagous, omnivorous, and herbivorous. We list and discuss these eco-morphotypes as follows:

1. **Insectivorous**: pterosaurs having insectivorous feeding adaptations included several non-pterodactyloid clades: the Late Triassic Eopterosauria, Jurassic Dimorphodontia + Campylognathoididae + Darwinoptera, and the Jurassic-Cretaceous Anurognathidae. All of these were small to medium-sized pterosaurs, with a wingspan ranging from less than half a meter (*Anurognathus*: 0.4 m) to about 2 m (*Cuspicephalus*).

The small-bodied anurognathids have a delicately built skull, large orbits, and short, wide jaws equipped with small, pointed, and widely spaced teeth; all of these features have been regarded as indicative of preying on aerial insects [2–4,34,43].

The Darwinoptera, including the Wukongopteridae and *Pterorhynchus*, are medium-sized pterosaurs, having a long tail as a primitive feature but a large nasoantorbital fenestra and a long neck as derived features. Darwinopterans have slender and long jaws, but the teeth are of different types, having been described as spike-like, cone-shaped, or conical and robust. Although some species are assumed to have been predatory [18], their slender jaws and types of dentition, in keeping with lacking of any predatory body design (e.g. sharp claws), are more indicative of an insectivorous feeding habit, rather than a raptor-sort of adaptation [4].

The Early Jurassic Dimorphodontia and Campylognathoididae are widely accepted as insectivorous from studies of the dentition and jaw mechanics [3–5,34,35]. The dimorphodontians had a short and lightly built skull, and straight jaws with a large gape. Their sharply pointed anterior teeth were suitable for penetrating insect exoskeletons, while the much smaller posterior teeth are analogous to the many modern insect-eating reptiles [4,34]. Similar to the dimorphodontians, campylognathoidids also had a lightly built skull, relatively straight and slender jaws, and closely spaced and triangular teeth [4,34,35]. These features imply that campylognathoidids had similar feeding style as the former group.

The Triassic eopterosaurians have been regarded as insectivorous, piscivorous or both [2–5,34]. These are small pterosaurs, having a short and lightly built skull, and straight jaws with a large gape. Most of them have heterodont teeth with tricuspid crowns, showing the type of dentition possibly analogous to that of modern teiid lizards *Ameiva* and *Kentropyx* [34]. Of these, *Preondactylus*, *Peteinosaurus*, and *Austriadactylus* are thought to have been predominantly insectivorous based on the above-mentioned cranial and dental features [4,5,34]. Several other forms in the Eopterosauria (*Eudimorphodon, Caviramus* and *Raeticodactylus*) were also insectivorous, although perhaps not exclusively. Some authors [34] have suggested that these forms may have included scaly fish in their diet based on tooth wear, whereas others [5] have argued that the extent of dental wear could be simply caused by a slow rate of tooth replacement. Nevertheless, these Triassic forms share many similar cranial and dental features with other insectivorous eopterosaurians, and the results of our statistical analysis of the cranial and dental features indicated that these cannot be unambiguously distinguished from other eopterosaurians, which were predominantly insectivorous pterosaurs (see Morphospace section below).

1. **Piscivorous:** Piscivorous pterosaurs included both toothed and toothless species. Toothed forms in the Rhamphorhynchidae and Ornithocheirae are characterized by having a “fish grabbing” dentition with enlarged anterior teeth in both upper and lower jaws [2–4,35]. Their piscivorous habit in life is corroborated with evidence from stomach contents in some rhamphorhynchid specimens [2,44].

The Nyctosauridae and Pteranodontidae are toothless piscivores, having long narrow and up-curved jaws in most taxa. These are known mainly from Upper Cretaceous marine beds in North America, and their piscivorous adaptations are also corroborated from evidence of stomach contents of fish remains [2–4,33].

The Pterodactylidae, Germanodactylidae, and Gallodactylidae are also considered mainly piscivorous, although their diet probably included other food sources. These pterosaurs are known by fossils from Upper Jurassic marine deposits in Germany and adjacent areas, implying they lived in environments with fish and invertebrates abundantly available as food resources.

Pterodactylids have a lightly-built skull and slender, long jaws. Their small and conical teeth are similar in size along the tooth row, but lacking anteriorly enlarged ‘fish grabbing’ teeth. Supported by direct evidence of stomach contents (fish remains) and indirect evidence of preservation of impressions of gular sac, pterodactylids have been interpreted as mainly fed by gleaning small fish from shallow water, although invertebrates may also be included in their diet [2,3,4].

The Germanodactylidae have been grouped with the Dsungaripteridae as sister clades in the Dsungaripteroidea by some authors [22,45–48], but rejected by recent phylogenetic analyses ([8,21,49,50], this study). The teeth of germanodactylids are more similar to those of pterodactylids than to those of dsungaripterids, implying similar feeding adaptations as in pterodactylids.

Gallodactylids have a structurally bizarre beak, with as many as 12 peg-like rostral teeth, upward-curved rostrum and downward-curved jaws [4,51]. These bizarre pterosaurs have been interpreted as having varied types of feeding styles, such as feeding on jellyfish or epizoan parasitic organisms, or as filter feeders like flamingoes [51]; or shell-crushing as open-billed storks, or pinecone feeders such as crossbills [4]. However, the jaws of gallodactylids have elongate peg-like teeth rostrally and no horny beak, evidence refuting possibilities of shell-crushing or pinecone feeding. A small number of peg-like teeth and downward-curved jaws are evidently unsuitable for filter-feeding habits. Based on the available evidence, gallodactylids can be parsimoniously interpreted as mainly piscivorous, like extant pelicans, but might also have fed on small prey (invertebrates) in shallow water.

(3) **Filter-feeding**: Filter feeding is best known for the Ctenochasmatidae, and possibly some but not all species in the Boreopteridae [2–4].

The Ctenochasmatidae are characterized by long jaws and the so-called ‘comb-dentition’ as filter-feeding adaptations [2–4]. Within the family, *Ctenochasma* has more than 200 needle-like teeth. These teeth are densely packed (interdental space= 1 mm), suitable for filtering small prey. Two other forms, *Gnathosaurus* and *Plataleorhynchus*, had their jaws spatulated anteriorly, and carried over 100 delicate teeth. The anterior teeth are elongated and laterally-oriented. These features are indicative of a feeding style like that of extant spoonbills [2–4]. Interdental space of these forms implies that their closely spaced teeth could filter out and hold prey larger than 2.5 mm in diameter. Several other forms in the family Ctenochasmatidae (*Gegepterus*, *Feilongus*, and *Moganopterus*) are also characterized by elongated jaws bearing a varied number of needle-like teeth. *Gegepterus* has over 100 teeth as commonly seen in other ctenochasmatids; whereas *Feilongus* and *Moganopterus* have long jaws but relatively short tooth rows (64–76 teeth) confined to anterior one-third of the jaw. Loss of posterior teeth in the latter two forms is probably a derived feature acquired within the ctenochasmatid clade.

Of all known pterosaurs, *Pterodaustro* is the most specialized filter-feeder, characterized by its greatly elongated and upward-curved jaws, numerous extremely elongate and densely packed mandibular teeth, and rows of ossicles along the upper jaws. Its diet possibly included tiny planktonic prey, seeds, tiny arthropods and mollusks [2–4,52,53].

The Boreopteridae are another family with a high number of strongly elongated and slender teeth. Within the family, *Boreopterus* had 116 teeth, and *Zhenyuanopterus* had 184 teeth. The teeth are about ten times taller than wide in anterior series, and steadily decreased in height posteriorly along the tooth row. The slender teeth are evenly spaced, and suitable for combing water for small prey [4]. These characters are generally compatible to those of the Ctenochasmatidae, implying a similar filter-feeding style convergent to that of the Ctenochasmatidae. The interdental space of boreopterids, however, is about 10 mm in *Boreopterus* and *Zhenyuanopterus*, much wider than in ctenochasmatids, suitable for filtering larger prey. Interestingly, *Guidraco* as the basalmost taxon within the boreopterid clade ([8], this study) was piscivorous based on its “fish grabbing” dentition enhanced by evidence of coprolites [54]. We color-coded the boreopterid clade as polymorphic (piscivorous + filter-feeding) in our figure 3*c,d*.

(4) **Carnivorous**: The Azhdarchidae and Istiodactylidae are identified as carnivorous. Azhdarchids were giraffe-sized pterosaurs, and were almost globally distributed. The skull is long and low-profiled, bearing a long and edentate beak. This group has been speculated as scavenging, carnivorous, fish-eating, or omnivorous feeders [2,4,33,37,55,56]. Based on their huge body size, however, terrestrial foraging with a carnivorous diet appears to be plausible interpretations for azhdarchids [4,37].

The Istiodactylidae were possibly scavengers as evidenced by their laterally compressed, razor-edged and interlocked teeth [4,34,38,57].

(5) **Durophagous**: This type of feeding adaptation is only known for the Dsungaripteridae, from Lower Cretaceous lacustrine deposits in China, Mongolia and Chile [4]. Jaws of these pterosaurs are anteriorly toothless, forming a tweezer-like beak for picking up shelled prey. Teeth are short, blunt, robust, and are only developed on posterior part of the jaw. This type of dentition, corroborated with their chunky skull and powerful jaws, is suitable for crushing shells of bivalve mollusks or crustaceans [2–4].

(6) **Herbivorous**: A total of 13–14 species in the Tapejaridae are thought to have been frugivorous. Fossils of these pterosaurs are known from Lower Cretaceous beds in Brazil, Morocco, Spain, and China. Tapejarids were small to medium-sized, having a high-profiled skull with a prominent bony crest on top of the skull roof. They had small eye sockets, a short but pointed snout, and a parrot-like beak. Their parrot-like beak has been interpreted as suitable for feeding on fruits, assisted by their high bony crest of the skull [3,4,39–41]. No evidence from stomach contents has been reported, but frugivorous is probably the most plausible interpretation, although this type of feeding habit had to be seasonal, with the diet of these pterosaurs perhaps also including seeds, insects, or other invertebrates [4,13].

(7) **Omnivorous**: Pterosaurs identified as omnivorous included those in the Lonchodectidae, Thalassodromidae and Chaoyangopteridae [3,4,58,59].

The Lonchodectidae were small to medium-sized, with a wingspan at about 2 m. This group is known by fossils from Lower Cretaceous deposits in the United Kingdom, Brazil, and possibly China. Lonchodectids have been hypothesized as having a life-style similar to seagulls today [3], but limb proportions similar to those of azhdarchoids suggests that they were more likely to have been terrestrial ‘walkers’ [4]. They have many short and laterally compressed teeth that point to no specific kind of diet. Based on available evidence mainly from teeth, lonchodectids were probably generalized feeders, and are treated as omnivorous in this study.

The Thalassodromidae and Chaoyangopteridae were medium-sized and edentulous pterosaurs that lived during Early Cretaceous time. Having a relatively wide skull with a short and straight beak, thalassodromids have been thought to be piscivorous by skim-feeding [59]; however, this hypothesis has been rejected, and with a stork-like or even predatory lifestyle suggested as an alternative [4,3758,60]. Chaoyangopterids have a low-profile skull with a long beak similar to that of carnivorous azhdarchids, but their medium body size seems unsuitable for predation on relatively large and agile animals. Instead, these were probably more or less omnivorous, with a diet that included small animals, nutritious plants, or even carrion [4]. Our statistical analysis lumps the Thalassodromidae and Chaoyangoteridae together with other toothless forms, which included both long-beaked carnivorous and short-beaked herbivorous pterosaurs.

**Morphospace of Pterosaur Feeding Adaptations**

We performed two separate statistical analyses: metric multidimensional scaling (MDS) analysis was conducted to provide visualization of the level of similarity/dissimilarity of pterosaur species in terms of feeding-related morphological features; permutational multivariate analysis of variance (PERMANOVA, [27]) was conducted to compare multivariate means of recognized different eco-morphological types among pterosaur taxa. The two separate statistical analyses were based on a total of 34 morphological characters (table S1) selected from the published dataset in reference [8]. These characters were coded for 89 species that have available information on inferred feeding adaptations (table S2). Other 20 species were excluded because none of these can be coded with more than 25% of the characters used in our statistical analyses.

For MDS analysis, we first used the software package PAST 3.0 [26] to obtain pairwise Gower distance from the tabulated characters. Missing data (unknown or inapplicable) were coded as “?”, and were treated as pairwise deletion; that is, if the value of a character is missing in one of the two taxa in a pair for comparison, this character is omitted from the computation of the distance between those two taxa. After this step, the Gower distance matrix was subjected to GINKGO v.1.7.0 [61] for MDS analysis. The obtained scores of the sampled 89 pterosaur species along three MDS axes were used to build two-dimentional MDS ordination plots as shown in figure 3*c,d*.

PERMANOVA analysis was performed in PAST 3.0 based on Gower distance. Different types of pterosaur feeding adaptations were segregated into ten categories as a priori (see table S3). Pairwise comparisons resulted significant segregation of different feeding adaptations among most pterosaur taxa, whereas a few pterosaurs (3 out of 45 pairwise comparisons) resulted non-significant segregation (see table S3). The non-significant results of the three pairwise comparisons may be influenced by small sample size and/or morphological similarities by convergent evolution.

Morphospace patterns as shown in MDS ordinate plots support partitioning of different eco-morphological types (figure 3*c*,*d*). Insectivorous taxa and piscivorous rhamphorhynchids are well separated; filter-feeding taxa partly overlap piscivorous rhamphorhynchids. Three families the in Archaeopterodactyloidea inferred as piscivorous (Pterodactylidae + Germanodactylidae + Gallodactylidae) are clustered in a small area between insectivorous and filter-feeding blocks. Herbivorous, Cretaceous dentate piscivorous, and dentate carnivorous taxa are set well apart, with each occupying a small area separated from the other along at least one MDS axis. Shell-crushers, omnivores, and edentate piscivores seem to converge to their own centroid in the morphospace, but they are usually embedded within the occupied areas of other feeding categories.

**Geological Setting**

The holotype and the only known specimen (PMOL-AP00031) of the new pterosaur (*Liaodactylus* *primus* gen. et sp. nov.) was found at a locality near Daxishan village (figure S1), Linglongta township, Jianchang County, western Liaoning Province, China. In the past few years, the Daxishan locality has received a considerable amount of attention because of the discovery of important vertebrate fossils, including paravian dinosaurs, pterosaurs, and early eutherian mammals [14,15,18,62,63]. The fossil beds cropping out at the Daxishan locality pertain to the Upper Jurassic Tiaojishan Formation (also called the Lanqi Formation in some literature), outcrops of which are widely distributed in the Western Hills of Beijing, Hebei and Liaoning provinces. The formation has yielded high-precision ^40^Ar/^39^Ar dates of 161.8 ± 0.4 Myr near its base, and 159.5 ± 0.6 Myr for the uppermost part in the Beipiao area, western Liaoning Province [12,64]. In terms of geologic contacts, the Tiaojishan Formation disconformably overlays the Middle Jurassic Haifanggou Formation [65]. The Tiaojishan Formation as exposed in the Daxishan section has been subdivided into three members [11,66,67]: both the lower and the upper members of the formation in this section are composed of extrusive volcanic rocks, mainly andesites, and are measured as >41 m and >650 m in thickness, respectively. The middle member, >546 m thick, consists of a sequence of lacustrine sandstones, siltstones, mudstones and volcanic shales. The middle member contains three fossil-bearing horizons, confined to a short sequence of *ca*. 100 m in thickness. The new pterosaur specimen was collected from the tuffaceous shales of the upper part of the middle member, along with other fossil material, including fishes, turtles, conchostracans, and plants. The fossil-bearing horizon has been dated at 160.5 ± 0.99 Myr [68]. The top part of the member has been dated at 159.5 ± 2.3 Myr, and the lower part of this member at 160.7 ± 1.7 Myr [11]. These dates are largely compatible with the high-precision range of 161.8 ± 0.4–159.5 ± 0.6 Myr from the Beipiao area [12,64].

Vertebrate fossils found at the Daxishan locality include fishes, amphibians, turtles, pterosaurs, feathered dinosaurs, and therian and non-therian mammals, all from the middle member of the Tiaojishan Formation. These are important components of the Yanliao Biota, immediately predating the Early Cretaceous Jehol Biota. Although a recent paper [69] has referred to the Daxishan fossil assemblage as part of the “Daohugou Biota” (*sensu lato*), the term “Yanliao Biota” is the more appropriate and has long been clearly defined by previous authors [13,70,71]. Fossils of the Yanliao Biota are mainly known from the Middle Jurassic Haifanggou Formation and the Upper Jurassic Tiaojishan Formation. Besides the Daxishan locality, vertebrate fossils from other localities within the Tiaojishan Formation include important salamander specimens from the Reshuitang and Guancaishan localities near Lingyuan and Jianping, respectively [16,17]. More recently, significant fossil discoveries have been reported from a locality near Qinglong, Hebei Province, yielding salamanders [17], pterosaurs [20,72–74], non-avian dinosaurs [75], and mammals [76,77].

**Anatomical Description of the Holotype (PMOL-AP00031)**

The skull (PMOL-AP00031) has a total length of 133 mm from the rostral tip of the snout to the posterior extremity of the squamosal. The skull is lightly built, with an elongated snout and several large fenestrae. The naris is confluent with the antorbital fenestra, forming a large nasoantorbital fenestra. Presence of such a large fenestra is a diagnostic feature of the Monofenestrata, the clade that includes the Pterodactyloidea, Darwinoptera and the Anurognathidae [8,18]. The nasoantorbital fenestra (41.4 mm long) is 31% of the total skull length, a plesiomorphic condition in the Ctenochasmatidae: the opening is reduced to much smaller proportions in other taxa of the family (*ca*. 28% of the skull length in *Gnathosaurus*; 12.6% in *Ctenochasma*; 10–12% in *Pterodaustro*). Furthermore, *Liaodactylus* exhibits several character states shared with other members of the Ctenochasmatidae: the dorsal margin of the skull is slightly concave in lateral view (except in *Gnathosaurus*), the quadrate shaft is strongly inclined to a subhorizontal position, and more than 128 slender and closely spaced teeth are contained in the jaws.

The slender rostrum (66.5 mm long), formed by the premaxilla and maxilla, is 49.1% of the total skull length. This ratio is smaller than in other ctenochasmatids (figure S3), in which the elongate rostrum is about 54% in *Gnathosaurus*, and 64% in *Ctenochasma*, and an extreme condition in *Pterodaustro*, more than 85% of the skull length [52]. The rostrum is parallel-sided, similar to all other ctenochasmatids but *Gnathosaurus* and *Plataleorhynchus*, in which the rostrum terminates in a spoon-shaped expansion (figure S3). The upper tooth row extends posteriorly, terminating below the anterior one-third of the nasoantorbital fenestra. This is a unique feature of the new taxon, differing from other ctenochasmatids, in which the tooth row is entirely anterior to the nasoantorbital fenestra (figure S3).

The jugal is slender, bearing a long and tapering anterior process, a subvertical orbital process, and a strongly inclined temporal process in parallel with the quadrate. The anterior process is extremely slender and elongated, forming the posterior two-thirds of the lower border of the nasoantorbital fenestra. Below the orbit, the main body of the jugal is strongly narrowed, in contrast to a much deeper condition in other ctenochasmatids (figure S3). The temporal process is significantly longer than the orbital process, probably a primitive condition within the Ctenochasmatidae. The suture between the jugal and quadratojugal is indistinguishable because of extensive fusion of the two bones. Below the large orbit, the jugal bends posteroventrally, forming a slightly curved buccal margin. The quadrate is dorsally in articulation with the squamosal, and ventrally with the lower jaw. The quadrate shaft is strongly inclined, setting a 160-degree angle with the buccal margin, a derived feature shared with other ctenochasmatids. The cranio-mandibular joint is located directly below the orbit.

Dorsally, the braincase is roofed by the frontal and parietal, and the two bones have no clear suture line because of fusion. This part of the skull roof rapidly turns downward posteriorly, forming a rounded posterior margin of the skull in lateral view.

In palatal view, the premaxillae are largely obscured because the upper and lower jaws are preserved in interlocking positions. The posterior part of the maxilla is exposed as a wide palatal shelf (flange) with a straight medial border for the large interpterygoid vacuity. The posterior end of the palatal shelf is deeply notched for the suborbital fenestra, with the ectopterygoid closing the posterior rim of the fenestra. The palatine is a slender spike, in articulation with the maxilla along the medial border of the suborbital fenestra. The ectopterygoid is triradiate, with a widened lateral process closing the posterior rim of the suborbital fenestra. A short anterior process meets the slender process of the palatal shelf of the maxilla. The posterior process overlaps the pterygoid and forms a large part of the middle rim of the pterygo-ectopterygoid fenestra. The pterygoid bears a thin lateral bar completely separating the pterygo-ectopterygoid fenestra from the subtemporal fenestra. This lateral process is present but extremely short in *Gnathosaurus* [78,79], whereas the actual condition is unknown for other members of the Ctenochasmatidae. The pterygoid is medially expanded, but a midline contact with its opposite element is obscured by the right mandible. The pterygoid also sends a posterior process to articulate with the basisphenoid and quadrate, forming the medial border of the subtemporal fenestra.

The basisphenoid is a short and wide median plate, anteriorly notched for the posterior border of the interpterygoid vacuity. Anterolateral to the notch, which is widely U-shaped, are two stout basipterygoid processes that contact both the pterygoid and quadrate. Posteriorly, the basisphenoid is constricted transversely, and then is partially overlapped by the basioccipital. The occipital condyle is spherical, whereas the foramen magnum is obscured by the atlas-axis complex. A rugose ridge extends along the midline of the basioccipital anterior to the occipital condyle.

The mandible is slenderly built, straight, and is 117 mm long. The dentary is the main element of the mandible, whereas the surangular and angular have a little exposure posteriorly on dorsal and ventral margin of the mandible, respectively. The dentary symphysis is 30.5% of the mandibular length, proportionally shorter than in other ctenochasmatids (figure S3). The retroarticular process is formed by the angular, and is well developed as in other ctenochasmatids.

The new pterosaur has a total of more than 152 teeth in its jaws. This total number of marginal teeth is greater than in *Gnathosaurus* (128–136; [45]), similar to that in *Gegepterus* (150; [80]), but is significantly smaller than *Ctenochasma* (200–552; [30]) and *Pterodaustro* (nearly a thousand teeth; [52]). The upper jaws of the new pterosaur carry 82 teeth, a slightly greater number than in the lower jaws (70 teeth). The first tooth in both the upper and lower jaws is short, only half the size of the second. Other anterior teeth are slender, long, slightly curved, and outward projecting. Those in the middle and posterior part of the tooth row gradually decrease in size posteriorly, and become short and peg-like at the end of the tooth row. All of the teeth are equally spaced, with a mean interdental space of about 2 mm, nearly as wide as the maximum diameter of each tooth. With the jaws closed, the upper and lower teeth are in interlocking positions (figure S2), forming a filter-feeding apparatus as in other ctenochasmatids.

The atlas-axis complex is exposed in ventrolateral view. The two cervical vertebrae are fused along a recognizable trace of suture. In addition, the proatlas is a single element, having two symmetrical, plate-like portions divided by a midline and an anterior process (figure 2*c,d*). The atlas portion of the complex displays a rounded and cup-shaped cotyle anteriorly for articulation with the spherical occipital condyle. Posterodorsally, the atlas portion bears a prominent neural spine partly exposed in ventral view. The axis portion displays the centrum as rectangular in ventrolateral view, and the neural arch carries a postzygapophysis for articulation with the first trunk vertebra, but the neural spine is not exposed.

**Supplementary References**

**Cited in Main Text and in Supplementary Information**

1. Padian K. 1985 The origins and aerodynamics of flight in extinct vertebrates. *Palaeont.* **28**, 413–433.
2. Wellnhofer P. 1991 *The Illustrated Encyclopedia of Pterosaurs.* London: Salamander Books.
3. Unwin DM. 2006. *The Pterosaurs from Deep Time.* New York: Pi Press.
4. Witton MP. 2013 *Pterosaurs: Natural History, Evolution, Anatomy.* Princeton and Oxford: Princeton Univ. Press.
5. Dalla Vecchia FM. 2013 Triassic pterosaurs. In *Anatomy, Phylogeny, and Palaeobiology of Early Archosaurs and their Kin*, vol. 379 (eds SJ Nesbitt, JB Desojo, RB Irmis), pp. 119–155. London: Geological Society of London, Special Publications.(doi:10.1144/SP379.14)
6. Barrett PM, Butler RJ, Edwards NP, Milner AR. 2008 Pterosaur distribution in time and space: an atlas. *Zitteliana* **B28**, 61–107.
7. Butler RJ, Brusatte SL, Andres B, Benson RBJ. 2012 How do geological sampling biases affect studies of morphological evolution in deep time? A case study of pterosaur (Reptilia: Archosauria) disparity. *Evolution* **66**, 147–162. (doi:10.1111/j.1558-5646.2011.01415.x)
8. Andres B, Clark J, Xu X. 2014 The earliest pterodactyloid and the origin of the group. *Curr. Biol.* **24**, 1011–1016. (doi:10.1016/j.cub.2014.03.030)
9. Benson RBJ, Frigot RA, Goswami A, Andres B, Butler RJ. 2014 Competition and constraint drove Cope’s rule in the evolution of giant flying reptiles. *Nat. Comm.* **5**, 3567. (doi:10.1038/ncomms4567)
10. Foth C, Brusatte SL, Butler RJ. 2012 Do different disparity proxies converge on a common signal? Insights from the cranial morphometrics and evolutionary history of Pterosauria (Diapsida: Archosauria). *J. Evol. Biol.* **25**, 904–915. (doi:10.1111/j.1420-9101.2012.02479.x)
11. Wang LL, Hu DY, Zhang LJ, Zheng SL, He HY, Deng CL, Wang XL, Zhou ZH, Zhu RX. 2013 SIMS U-Pb zircon age of Jurassic sediments in Linglongta, Jianchang, western Liaoning: Constraint on the age of oldest feathered dinosaurs. *Chin. Sci. Bull. (Chin. Ver.)* **58**, 1346–1353. (doi:10.1360/972012-535)
12. Chang S-C, Zhang H, Hemming SR, Mesko GT, Fang Y. 2014 ^40^Ar/^39^Ar age constraints on the Haifanggou and Lanqi formations: When did the first flowers bloom? In *Advances in ^40^Ar/^39^Ar Dating: from Archaeology to Planetary Sciences*, vol. 378 (eds F Jourdan, DF Mark, C Veratt), pp. 277–284. London: Geological Society of London, Special Publications.(doi:10.1144/sp378.1)
13. Zhou ZH, Wang Y. 2010 Vertebrate diversity of the Jehol Biota as compared with other lagerstätten. *Sci. China Earth Sci.* **53**, 1894–1907. (doi:10.1007/s11430-010-4094-9)
14. Hu D, Hou L, Zhang L, Xu X. 2009 A pre-*Archaeopteryx* troodontid theropod from China with long feathers on the metatarsus. *Nature* **461**, 640–643. (doi:10.1038/nature08322)
15. Luo Z-X, Yuan C-X, Meng Q-J, Ji Q. 2011 A Jurassic eutherian mammal and divergence of marsupials and placentals. *Nature* **476**, 442–445. (doi:10.1038/nature10291)
16. Gao K-Q, Shubin NH. 2012 Late Jurassic salamandroid from western Liaoning, China. *Proc. Natl. Acad. Sci. USA* **109**, 5767–5772. (doi:10.1073/pnas.1009828109)
17. Gao K-Q, Chen J, Jia J. 2013 Taxonomic diversity, stratigraphic range, and exceptional preservation of Juro-Cretaceous salamanders from northern China. *Can. J. Earth Sci.* **50**, 255–267. (doi:10.1139/e2012-039)
18. Lü J, Unwin DM, Jin X, Liu Y, Ji Q. 2010 Evidence for modular evolution in a long-tailed pterosaur with a pterodactyloid skull. *Proc. R. Soc. B* **277**, 383–389. (doi:10.1098/rspb.2009.1603)
19. Cheng X, Wang X, Jiang S, Kellner AWA. 2012 A new scaphognathid pterosaur from western Liaoning, China. *Hist. Biol.* **24**, 101–111. (doi:10.1080/08912963.2011.635423)
20. Lü J, Hone DWE. 2012 A new Chinese anurognathid pterosaur and the evolution of pterosaurian tail lengths. *Acta Geol. Sin.* **86**, 1317–1325.
21. Kellner AWA. 2003 Pterosaur phylogeny and comments on the evolutionary history of the group. In *Evolution and Palaeobiology of Pterosaurs*, vol. 217 (eds E Buffetaut, JM Mazin), pp. 105–137. London: Geological Society of London, Special Publications.
22. Unwin DM. 2003 On the phylogeny and evolutionary history of pterosaurs. In *Evolution and Palaeobiology of Pterosaurs*, vol. 217 (eds E Buffetaut, JM Mazin), pp. 139–190. London: Geological Society of London, Special Publications.
23. Wang X, Rodrigues T, Jiang S, Cheng X, Kellner AWA. 2014 An Early Cretaceous pterosaur with an unusual mandibular crest from China and a potential novel feeding strategy. *Sci. Rep*. **4**, 6329. (doi:10.1038/srep06329)
24. Goloboff PA, Farris J, Nixon K. 2008 TNT: tree search using new technology, vers. 1.1, Willy Hennig Society Education; http://www.zmuc.dk/public/phylogeny/tnt.
25. Goloboff PA, Mattoni CI, Quinteros AS. 2008 TNT, a free program for phylogenetic analysis. *Cladistics* **24**, 774–786. (doi:10.1111/j.1096-0031.2008.00217.x)
26. Hammer Ø, Harper DAT, Ryan PD. 2001 PAST: Paleontological Statistics Software Package for education and data analysis. *Palaeont. Electron.* **4**, 1–9.
27. Anderson MJ. 2001 A new method for non-parametric multivariate analysis of variance. *Austral Ecol.* **26**, 32–46.
28. Cohen KM, Finney SC, Gibbard PL, Fan J-X. 2013 The ICS International Chronostratigraphic Chart. *Episodes* **36**, 199–204.
29. Wellnhofer P. 1970 Die Pterodactyloidea (Pterosauria) der Oberjura-Plattenkalke Süddeutschlands. *Bayer. Akad. Wiss., Math.-Wiss. Kl., Abh*. **141**, 1–133.
30. Bennett SC. 2007 A review of the pterosaur *Ctenochasma*: taxonomy and ontogeny. *N. Jb.* *Geol. Paläont. Abh*. **245**, 23–31. (doi:10.1127/0077-7749/2007/0245-0023)
31. Bonaparte J. 1971 Descripción del Cráneo y Mandíbulas de *Pterodaustro guinazui* (Pterodactiloidea-Pterodaustriidae nov.) de la Formación Lagarcito, San Luis, Argentina. *Publ. Mus. Mun. Cienc. Nat.Mar del Plata* **1**, 263–272.
32. Choiniere JN, Clark JM, Forster CA, Xu X. 2010 A basal coelurosaur (Dinosauria: Theropoda) from the Late Jurassic (Oxfordian) of the Shishugou Formation in Wucaiwan, People’s Republic of China. *J. Vert. Paleont.* **30**, 1773–1796. (doi:10.1080/02724634.2010.520779)
33. Bennett SC. 2001 The osteology and functional morphology of the Late Cretaceous pterosaur *Pteranodon*: Part I. General description of osteology. *Palaeontographica Abt. A* **260**, 1–153.
34. Ösi A. 2011 Feeding-related characters in basal pterosaurs: implications for jaw mechanism, dental function and diet. *Lethaia* **44**, 136–152. (doi:10.1111/j.1502-3931.2010.00230.x)
35. Padian K. 2008 The Early Jurassic pterosaur *Campylognathoides* Strand, 1928. *Spec. Pap. Palaeont.* **80**, 65–107. (doi:10.1111/j.1475-4983.2008.00795.x)
36. Witton MP. 2012 New insights into the skull of *Istiodactylus latidens* (Ornithocheiroidea, Pterodactyloidea). *PLoS ONE* **7**, e33170. (doi:10.1371/journal.pone.0033170)
37. Witton MP, Naish D. 2008 A reappraisal of azhdarchid pterosaur functional morphology and paleoecology. *PLoS ONE* **3**, e2271. (doi:10.1371/journal.pone.0002271)
38. Howse SCB, Milner AR, Martill DM. 2001 Pterosaurs. In *Dinosaurs of the Isle of Wight* (eds DM Martill, D Naish), pp. 324–335. London: Palaeontological Association.
39. Wellnhofer P, Kellner AWA. 1991 The skull of *Tapejara wellnhoferi* Kellner (Reptilia, Pterosauria) from the Lower Cretaceous Santana Formation of the Araripe Basin, Northeastern Brazil. *Mitt. Bayer Staatssam. Paläont. Hist. Geol.* **31**, 89–106.
40. Wang X, Zhou Z. 2006 Pterosaur assemblages of the Jehol Biota and their implication for the Early Cretaceous pterosaur radiation. *Geol. J.* **41**, 405–418. (doi:10.1002/gj.1046)
41. Vullo R, Marugán-Lobón J, Kellner AWA, Buscalioni AD, Gomez B, de la Fuente M, Moratalla JJ. 2012 A new crested pterosaur from the Early Cretaceous of Spain: the first European tapejarid (Pterodactyloidea: Azhdarchoidea). *PLoS ONE* **7**, e38900. (doi:10.1371/journal.pone.0038900)

**Additional References cited in SI and Supplementary Figure Captions**

1. Wiens JJ. 2001 Character analysis in morphological phylogenetics: problems and solutions. *Syst. Biol.* 50, 689–699.
2. Bennett SC. 2007 A second specimen of the pterosaur *Anurognathus ammoni*. *Paläont. Z.* **81**, 376–398. (doi:10.1007/BF02990250)
3. Bennett SC. 2014 A new specimen of the pterosaur *Scaphognathus crassirostris*, with comments on constraint of cervical vertebrae number in pterosaurs. *N. Jb. Geol. Paläeont. Abh.* **271**, 327–348. (doi:10.1127/0077-7749/2014/0392)
4. Wellnhofer P. 1978 *Pterosauria* (*Handbuch der Paläoherpetologie* 19). Stuttgart: Gustav Fischer Verlag.
5. Young CC. 1964 On a new pterosaurian from Sinkiang, China. *Vert. PalAsiat.* **8**, 221–238.
6. Young CC. 1973 *Reports of Paleontological Expedition to Sinkiang II.* Beijing: Science Press.
7. Lü J, Azuma Y, Dong Z, Barsbold R, Kobayashi Y, Lee Y-N. 2009 New material of dsungaripterid pterosaurs (Pterosauria: Pterodactyloidea) from western Mongolia and its palaeoecological implications. *Geol. Mag.* **146**, 690–700. (doi:10.1017/S0016756809006414)
8. Bennett SC. 2003 Morphological evolution of the pectoral girdle of pterosaurs: myology and function. In *Evolution and Palaeobiology of Pterosaurs*, vol. 217 (eds E Buffetaut, JM Mazin), pp. 191–215. London: Geological Society of London, Special Publications.
9. Andres B, Ji Q. 2008 A new pterosaur from the Liaoning Province of China, the phylogeny of the Pterodactyloidea, and convergence in their cervical vertebrae. *Palaeont.* **51**, 453–470. (doi:10.1111/j.1475-4983.2008.00761.x)
10. Bennett SC. 2013 The morphology and taxonomy of the pterosaur *Cycnorhamphus*. *N. Jb. Geol. Paläont. Abh.* **267**, 23–41. (doi: 10.1127/0077-7749/2012/0295)
11. Chiappe LM, Kellner AWA, Rivarola D, Davila S, Fox M. 2000 Cranial morphology of *Pterodaustro guinazui* (Pterosauria: Pterodactyloidea) from the Lower Cretaceous of Argentina. *Nat. Hist. Mus. Los Angeles County Contrib. Sci.* **483**, 1–19.
12. Codorniú L, Chiappe LM, Cid FD. 2013 First occurrence of stomach stones in pterosaurs. *J. Vert. Paleont.* **33**, 647–654. (doi:10.1080/02724634.2013.731335)
13. Wang X, Kellner AWA, Jiang S, Cheng X. 2012 New toothed flying reptile from Asia: close similarities between early Cretaceous pterosaur faunas from China and Brazil. *Naturwissenschaf.* **99**, 249–257. (doi:10.1007/s00114-012-0889-1)
14. Lawson DA. 1975 Pterosaur from the Latest Cretaceous of West Texas: Discovery of the largest flying creature. *Science* **187**, 947–948. (doi:10.1126/science.187.4180.947)
15. Kellner AWA, Langston W. 1996 Cranial remains of *Quetzalcoatlus* (Pterosauria, Azhdarchidae) from Late Cretaceous sediments of Big Bend National Park, Texas. *J. Vert. Paleont.* **16**, 222–231. (doi:10.1080/02724634.1996.10011310)
16. Martill DM. 2014 A functional odontoid in the dentary of the Early Cretaceous pterosaur *Istiodactylus latidens*: Implications for feeding. *Cret. Res.* **47**, 56–65. (doi: 10.1016/j.cretres.2013.11.005)
17. Unwin DM, Martill DM. 2007 Pterosaurs of the Crato Formation. In *Window into an Ancient World: The Crato Fossil Beds of Brazil* (eds Martill DM, Bechly G, Loveridge RF), pp. 475–524. London: Cambridge University Press.
18. Kellner AWA, Campos DA. 2002 The function of the cranial crest and jaws of a unique pterosaur from the Early Cretaceous of Brazil. *Science* **297**, 389–392. (doi:[10.1126/science.1073186](http://dx.doi.org/10.1126/science.1073186))
19. Humphries S, Bonser RHC, Witton MP, Martill DM. 2007 Did pterosaurs feed by skimming? Physical modelling and anatomical evaluation of an unusual feeding method. *PLoS Biol.* **5**, e204. (doi:10.1371/journal.pbio.0050204)
20. De Cáceres M, Oliva F, Font X, Vives S. 2007 GINKGO, a program for non-standard multivariate fuzzy analysis. *Adv. Fuzzy Sets. Syst.* **2**, 41–56.
21. Li Q, Gao K-Q, Vinther J, Shawkey MD, Clarke JA, D’Alba L, Meng Q, Briggs DE, Prum RO. 2010 Plumage color patterns of an extinct dinosaur. *Science* **320**, 1369–1372. (doi:10.1126/science.1186290)
22. Xu X, You H, Du K, Han F. 2011 An *Archaeopteryx*-like theropod from China and the origin of Avialae. *Nature* **475**, 465–470. (doi:10.1038/nature10288)
23. Chang S-C, Zhang H, Renne PR, Fang Y. 2009 High-precision 40Ar/39Ar age constraints on the basal Lanqi Formation and its implications for the origin of angiosperm plants. *Earth Planet Sci. Lett.* **279**, 212–221. (doi:10.1016/j.epsl.2008.12.045)
24. Chen P. 2003 Cretaceous biostratigraphy of China. In *Biostratigraphy of China*, (eds W-T Zhang, P-J Chen, AR Palmer), pp. 423–463. Beijing: Science Press.
25. Duan Y, Zheng S-L, Hu D-Y, Zhang L-J, Wang W-L. 2009 Preliminary report on Middle Jurassic strata and fossils from Linglongta area of Jianchang, Liaoning. *Global Geol.* **28**, 143–147.
26. Gao F-L, Wang M-C, Zhang G-R, Pan Y-Q. 2015 New discovery of the Middle Jurassic Yanliao Biota in Daxishan area of Linglongta, Liaoning Province. *Geol. Resour.* **24**, 7–11.
27. Liu Y-Q, Kuang H-W, Jiang X-J, Peng N, Xu H, Sun H-Y. 2012 Timing of the earliest known feathered dinosaurs and transitional pterosaurs older than the Jehol Biota. *Palaeogeogr. Palaeoclimatol. Palaeoecol.* **323-325**, 1–12. (doi:10.1016/j.palaeo.2012.01.017)
28. Sullivan C, Wang Y, Hong DWE, Wang Y, Xu X, Zhang F. 2014 The vertebrates of the Jurassic Daohugou Biota of northeastern China. *J. Vert. Paleont.* **34**, 243–280. (doi:10.1080/02724634.2013.787316)
29. Hong YC. 1983 *Middle Jurassic Fossil Insects in North China.* Beijing: Geological Publishing House.
30. Guo X-Q, Han J-G, Ji S-A. 2012 Advance in the study of vertebrate fossils of the Middle Jurassic Yanliao Biota in western Liaoning Province and adjacent areas. *Geol. Bull. China* **31**, 928–935.
31. Lü J-C. 2009 A new non-pterodactyloid pterosaur from Qinglong County, Hebei Province of China. *Acta Geol. Sin.* **83**,189–199.
32. Lü J, Unwin DM, Zhao B, Gao C, Shen C. 2012 A new rhamphorhynchid (Pterosauria: Rhamphorhynchidae) from the Middle/Upper Jurassic of Qinglong, Hebei Province, China. *Zootaxa* **3158**,1–19. (doi:10.11646/%25x)
33. Jiang S, Wang X, Cheng X, Costa FR, Huang J, Kellner AWA. 2015 Short note on an anurognathid pterosaur with a long tail from the Upper Jurassic of China. *Hist. Biol.* **27**, 718–722. (doi:10.1080/08912963.2014.954570)
34. Xu X, Zheng X, Sullivan C, Wang X, Xing L, Wang Y, Zhang X, O’Connor JK, Zhang F, Pan Y. 2015 A bizarre Jurassic maniraptoran theropod with preserved evidence of membranous wings. *Nature* **52**, 70–73. (doi:10.1038/nature14423)
35. Zheng X, Bi S, Wang X, Meng J. 2013 A new arboreal haramiyid shows the diversity of crown mammals in the Jurassic period. *Nature* **500**,199–202. (doi:10.1038/nature12353)
36. Luo Z-X, Meng Q-J, Ji Q, Liu D, Zhang Y-G, Neander AI. 2015 Evolutionary development in basal mammaliaforms as revealed by a docodontan. *Science* **347**, 760–764. (doi:10.1126/science.1260880)
37. Ösi A, Prondvai E, Frey E., Pohl B. 2010 New interpretation of the palate of pterosaurs. *Anat. Rec. A* **293**, 243–258. (doi:10.1002/ar.21053.)
38. Pinheiro FL, Schultz CL. 2012 An unusual pterosaur specimen (Pterodactyloidea, ?Azhdarchoidea) from the Early Cretaceous Romualdo Formation of Brazil, and the Evolution of the pterodactyloid palate. *PLoS ONE* **7**, e50088. (doi:10.1371/journal.pone.0050088)
39. Wang X, Kellner AWA, Zhou Z, Campos DA. 2007 A new pterosaur (Ctenochasmatidae, Archaeopterodactyloidea) from the Lower Cretaceous Yixian Formation of China. *Cret. Res.* **28**, 245–260. (doi:10.1016/j.cretres.2006.08.004)

**Supplementary figures S1-S3**


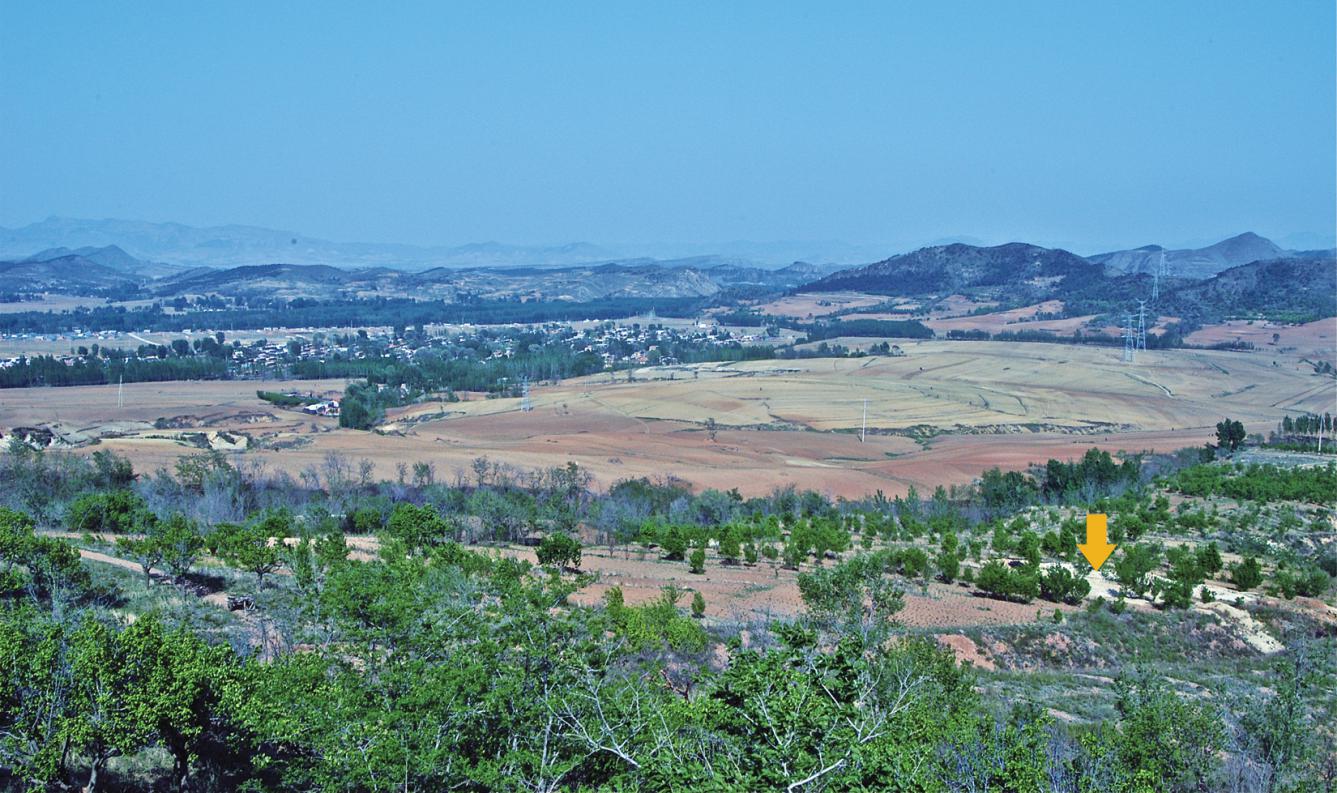


Figure S1. Photographic image of the Daxishan locality (arrow), showing fossil beds of the Upper Jurassic Tiaojishan Formation cropping out near Daxishan village.


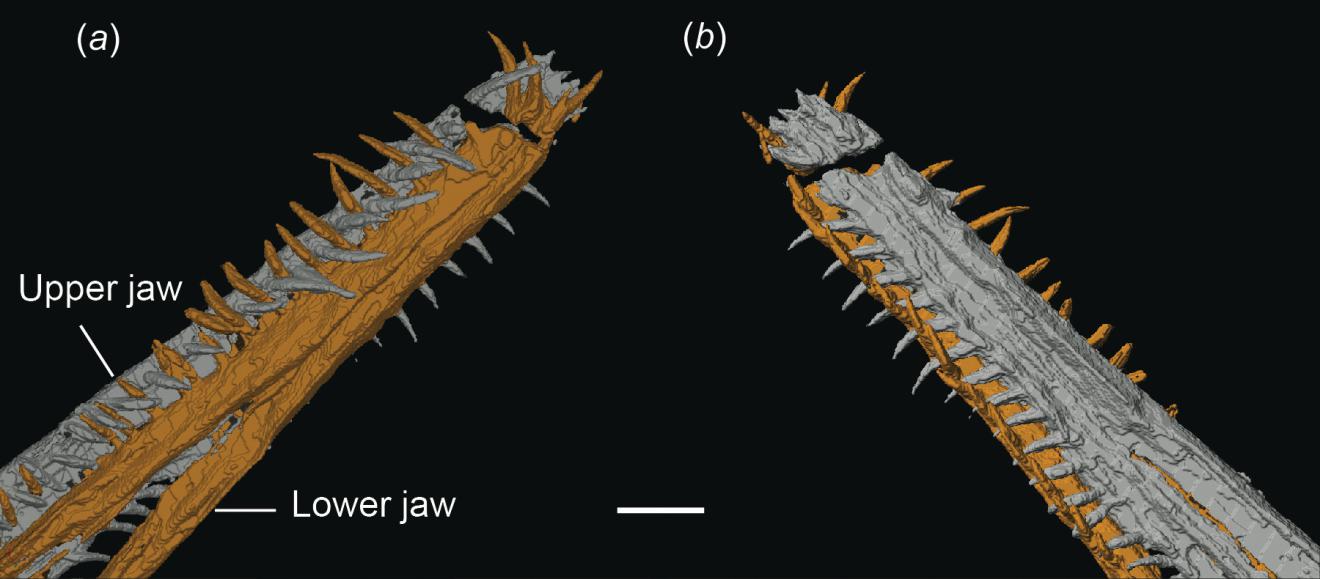


Figure S2. Reconstruction of PMOL-AP00031 (holotype of *Liaodactylus primus* gen. et sp. nov.) with interlocking jaws displayed in ventrolateral (*a*) and dorsolateral (*b*) views. Scale bar equals 5 mm.


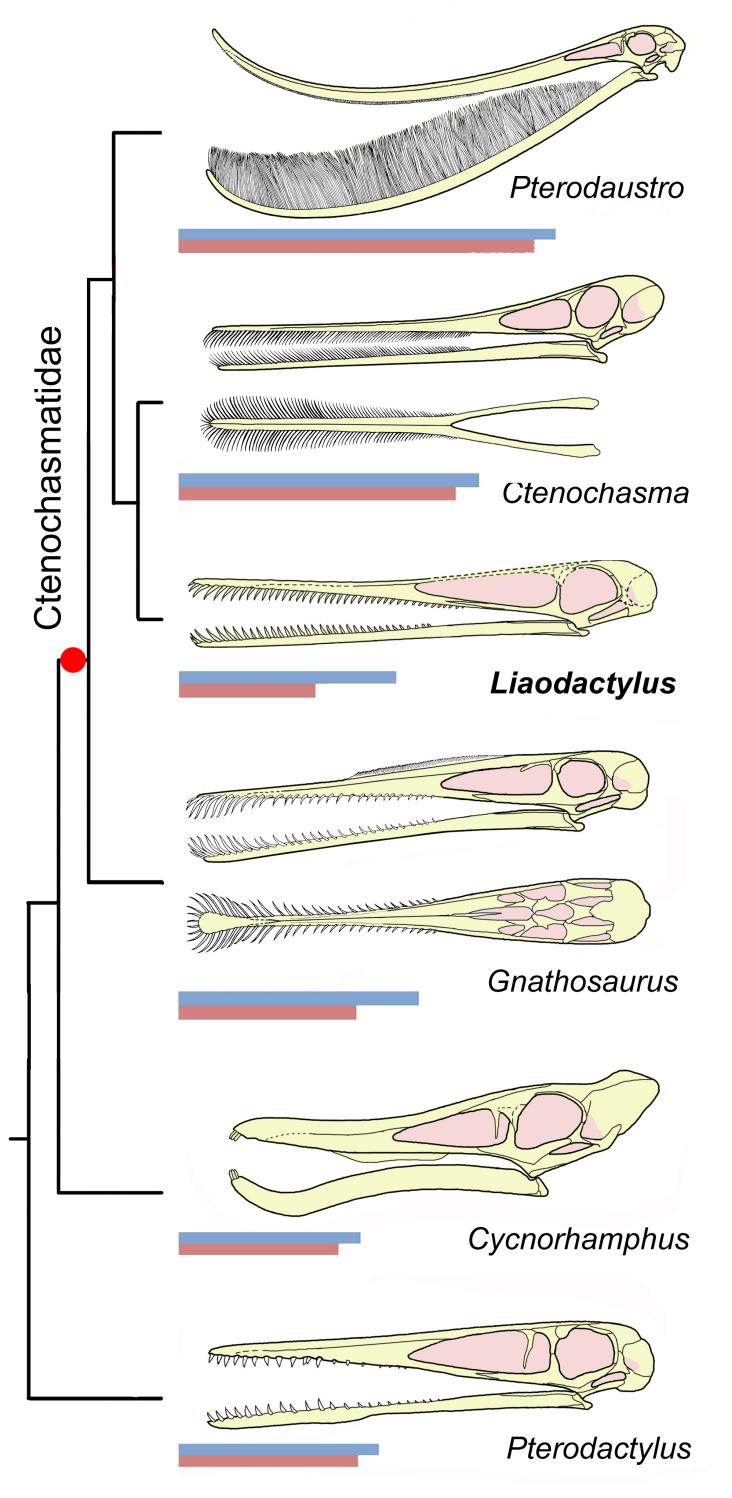


Figure S3. Skull and mandibles of ctenochasmatid pterosaurs, showing evolutionary increase of length of rostrum (blue bar) and mandibular symphysis (brown bar) within Ctenochasmatidae. Skulls of outgroup taxa *Cycnorhamphus* (Gallodactylidae) and *Pterodactylus* (Pterodactyllidae) are shown for comparison. Skull of *Pterodactylus*, *Ctenochasma*, *Gnathosaurus* and *Pterodaustro* adapted from reference [45]; *Cycnorhamphus* adapted from reference [51].

**Supplementary Tables**

Table S1. List of morphological characters used in statistical analyses. Characters are numbered as in reference [8].

| 0. Skull, length/height: values, 1.664-12.438; continuous coded 0-1. |
| --- |
| 1. Skull/dorsal vertebra in length: values, 6.348-59.164; continuous coded 0-1. |
| 2. Mandible/skull in length: values, 0.603-1.152; continuous coded 0-1. |
| 3. Rostrum/skull in length: values, 0.021-0.888; continuous coded 0-1. |
| 13. Rostral tooth row/skull in length: values, 0.051-0.807; continuous coded 0-1. |
| 14. Teeth number/1000: continuous coded 0-1. |
| 15. Mandibular symphysis/mandible in length: values, 0.052-0.688;  continuous coded 0-1. |
| 16. Mandibular tooth row/mandible in length: values, 0.228-0.884;  continuous coded 0-1. |
| 42. Rostrum, shape: (0) compressed laterally; (1) shortened anteroposteriorly; |
| (2) depressed dorsoventrally. |
| 43. Jaws, lateral taper: (0) attenuated; (1) subparallel; (2) wide. |
| 44. Rostrum anterior end: (0) upturned; (1) straight; (2) downturned. |
| 45. Rostrum anterior expansion: (0) absent; (1) present. |
| 46. Skull, anterior expansion, horizontal outline: (0) elliptical; (1) triangular;  (2) quadrangular. |
| 89. Quadrate, inclination relative to ventral margin of skull: (0) anteriorly;  (1) subvertical; (2) 120° posteriorly; (3) 150° posteriorly. |
| 116. Mandibular tip: (0) upturned; (1) straight; (2) downturned. |
| 125. Mandible mid-depth/length: (0) ≤1/9; (1) >1/9. |
| 135. Teeth: (0) present; (1) absent. |
| 136. Teeth variation in shape: (0) isodont; (1) heterodont. |
| 137. Mesial heterodont teeth: (0) recurved triangle; (1) slender needle; (2) recurved spike. |
| 138. Cheek teeth: (0) recurved triangle; (1) bulbous triangle; (2) slender needle;  (3) recurved cone; (4) labiolingually compressed triangle; (5) recurved spike. |
| 139. Teeth texture: (0) smooth; (1) striated; (2) sharp mesial and distal keels;  (3) medial carinae. |
| 140. Teeth, crown, height/width: (0) <4; (1) ≥4. |
| 141. Teeth, lateral orientation: (0) vertical; (1) inclined laterally. |
| 142. Teeth inter-space: (0) nearly touching; (1) less than teeth widths;  (2) subequal to teeth widths; (4) more than teeth widths. |
| 143. Teeth, size variation: (0) transition along tooth row; (1) sharp disparity in size between mesial and distal teeth. |
| 144. Upper/lower dentition in size: (0) upper much larger; (1) upper subequal or slightly larger; (2) much smaller than lowers. |
| 145. Teeth, displacement of maximum curvature: (0) less than tooth diameter; (1) at least tooth diameter. |
| 146. Teeth, curvature orientation: (0) posterior; (1) lingual. |
| 147. Teeth, inclination: (0) upright; (1) mesial teeth procumbent. |
| 149. Cheek teeth, multi-cusps: (0) present; (1) absent. |
| 150. Teeth, largest denticles: (0) serrations; (1) cuspules; (2) crenulations; (3) low cusps; (4) tall cusps. |
| 152. Rostral dentition, anteriorly positioned relative to rostral tip: (0) reaches tip; (1) posterior to tip. |
| 155. Lower dentition anteriorly positioned relative to mandibular tip: (0) reaches tip; (1) posterior to tip. |
| 156. Rostrum occlusal margin: (0) horizontal or ventrally reflected; (1) dorsally reflected. |

Table S2. Taxon-Character dataset used in statistical analyses. Dataset is from reference [8] with the addition of *Liaodactylus primus* in this study. Color shades correspond to varied feeding adaptations as color coded in figure 3.

| Feeding category | Taxon/Character | 0 | 1 | 2 | 3 | 13 | 14 | 15 | 16 | 42 | 43 | 44 | 45 | 46 | 89 | 116 | 125 |
| --- | --- | --- | --- | --- | --- | --- | --- | --- | --- | --- | --- | --- | --- | --- | --- | --- | --- |
| Insectivorous | *Eudimorphodon ranzii* | 0.166 | 0.189 | 0.475 | 0.28 | 0.814 | 0.12 | 0.043 | 0.704 | 0 | 0 | 1 | 0 | ? | 2 | 1 | 0 |
|  | *Eudimorphodon rosenfeldi* | 0.077 | 0.164 | 0.345 | 0.28 | 0.663 | 0.066 | 0.255 | 0.762 | 0 | ? | 1 | 0 | ? | 2 | 1 | 0 |
|  | *Eudimorphodon cromptonellus* | ? | ? | ? | ? | ? | 0.048 | ? | ? | ? | ? | ? | ? | ? | ? | ? | 0 |
|  | *Peteinosaurus zambellii* | ? | ? | ? | ? | ? | 0.148 | ? | ? | ? | ? | ? | ? | ? | ? | ? | 0 |
|  | *Caviramus schesaplanensis* | ? | ? | ? | ? | ? | 0.068 | 0.117 | ? | ? | ? | ? | 0 | ? | ? | 1 | 1 |
|  | *Raeticodactylus filisurensis* | 0.13 | 0.143 | 0.513 | 0.286 | 0.644 | 0.078 | 0.229 | 0.505 | 0 | ? | 1 | 0 | ? | 0 | 1 | 1 |
|  | *Austriadactylus cristatus* | 0.206 | 0.14 | 0.374 | 0.167 | 0.68 | 0.1 | 0.114 | 0.593 | 0 | 0 | 1 | 0 | ? | ? | 2 | 0 |
|  | *Preondactylus buffarinii* | 0.163 | 0.445 | 0.497 | 0.112 | 0.795 | 0.07 | 0.037 | 0.52 | 0 | ? | 1 | 0 | ? | ? | 1 | 0 |
|  | *Dimorphodon macronyx* | 0.177 | 0.288 | 0.534 | 0.203 | 0.732 | 0.124 | 0.116 | 0.658 | 0 | ? | 1 | 0 | ? | 0 | 1 | 0 |
|  | *Parapsicephalus purdoni* | 0.204 | ? | ? | ? | ? | ? | ? | ? | 0 | 0 | ? | ? | ? | 0 | ? | ? |
|  | *Campylognathoides liasicus* | 0.153 | 0.118 | ? | 0.245 | 0.746 | 0.056 | 0.108 | 0.688 | 0 | 0 | 1 | 0 | ? | 0 | 2 | 0 |
|  | *Campylognathoides zitteli* | 0.09 | 0 | 0.609 | 0.337 | 0.724 | 0.066 | 0.177 | 0.632 | 0 | 0 | 1 | 0 | ? | ? | 2 | 0 |
|  | *Sordes pilosus* | 0.178 | 0.254 | 0.221 | 0.202 | 0.584 | 0.026 | 0.38 | 0.373 | 0 | 0 | 1 | 0 | ? | 1 | 1 | 0 |
|  | *Darwinopterus modularis* | 0.277 | 1 | 0.422 | 0.374 | 0.573 | 0.054 | 0.259 | 0.487 | 0 | 0 | 1 | 0 | ? | 1 | 1 | 0 |
|  | *Wukongopterus lii* | 0.232 | ? | 0.469 | 0.29 | 0.587 | 0.056 | 0.223 | ? | 0 | 0 | 1 | 0 | ? | ? | 1 | 0 |
|  | *Pterorhynchus wellnhoferi* | 0.12 | 0.601 | 0.582 | 0.347 | 0.61 | 0.038 | 0.207 | 0.396 | 0 | 0 | 1 | 0 | ? | ? | 1 | 0 |
|  | *Batrachognathus volans* | ? | 0.113 | 0.682 | 0 | 0.772 | 0.048 | 0.02 | ? | 1 | 2 | 1 | 0 | ? | ? | 1 | 0 |
|  | *Jeholopterus ningchengensis* | 0.102 | 0.022 | 0.842 | 0.077 | 0.842 | 0.028 | 0 | 0.625 | 1 | 2 | 1 | 0 | ? | ? | 1 | 0 |
|  | *Anurognathus ammoni* | 0 | 0.148 | 0.526 | 0.024 | 0.579 | 0.03 | 0.178 | 0.213 | 1 | 2 | 1 | 0 | ? | ? | 1 | 0 |
|  | *Dendrorhynchoides curvidentatus* | ? | 0.073 | 0.876 | 0.099 | ? | ? | 0 | ? | 1 | 2 | 1 | 0 | ? | ? | ? | 0 |

| Feeding category | Taxon/Character | 135 | 136 | 137 | 138 | 139 | 140 | 141 | 142 | 143 | 144 | 145 | 146 | 147 | 149 | 150 | 152 | 155 | 156 |
| --- | --- | --- | --- | --- | --- | --- | --- | --- | --- | --- | --- | --- | --- | --- | --- | --- | --- | --- | --- |
| Insectivorous | *Eudimorphodon ranzii* | 0 | 1 | 0 | 1 | 1 | 0 | 0 | 0 | 1 | 1 | 0 | 0 | 1 | 0 | 4 | 0 | 1 | 0 |
|  | *Eudimorphodon rosenfeldi* | 0 | 1 | 0 | 1 | 0 | 0 | 0 | 0 | 1 | 1 | 0 | 0 | 1 | 0 | 4 | 0 | 1 | 0 |
|  | *Eudimorphodon cromptonellus* | 0 | ? | ? | 1 | 0 | 0 | 0 | 0 | ? | 1 | 0 | ? | 0 | 0 | 4 | ? | ? | ? |
|  | *Peteinosaurus zambellii* | 0 | 1 | 0 | 1 | 0 | 0 | 0 | 0 | 1 | ? | 0 | 0 | 0 | 0 | 2 | ? | 0 | 0 |
|  | *Caviramus schesaplanensis* | 0 | ? | ? | 1 | 0 | 0 | 0 | 0 | ? | ? | 0 | ? | 0 | 0 | 3 | ? | 0 | 1 |
|  | *Raeticodactylus filisurensis* | 0 | 1 | 2 | 1 | 1 | 0 | 0 | 0 | 1 | 0 | 0 | 0 | 1 | 0 | 3 | 0 | 1 | 0 |
|  | *Austriadactylus cristatus* | 0 | 0 | 1 | 0 | 1 | 1 | 0 | 0 | 0 | 1 | 0 | 0 | 0 | 0 | 0 | 1 | 0 | 0 |
|  | *Preondactylus buffarinii* | 0 | 1 | 0 | 1 | ? | 0 | 0 | 0 | 1 | 0 | 0 | 0 | 0 | 0 | 1 | 0 | 0 | 0 |
|  | *Dimorphodon macronyx* | 0 | 0 | ? | 0 | 1 | 0 | 0 | 1 | 1 | 0 | 0 | 0 | 0 | 1 | ? | 0 | 0 | 0 |
|  | *Parapsicephalus purdoni* | 0 | ? | ? | ? | ? | ? | 0 | 1 | ? | ? | ? | ? | 0 | ? | ? | ? | ? | 0 |
|  | *Campylognathoides liasicus* | 0 | 0 | ? | 0 | 0 | 0 | 0 | 1 | 1 | 1 | 0 | 0 | 0 | 1 | ? | 0 | 1 | 0 |
|  | *Campylognathoides zitteli* | 0 | 0 | ? | 0 | 0 | 0 | 0 | 1 | 1 | 1 | 0 | 0 | 0 | 1 | ? | 0 | 1 | 0 |
|  | *Sordes pilosus* | 0 | 0 | ? | 3 | 0 | 0 | 0 | 3 | 0 | 1 | 0 | 0 | 0 | 1 | ? | 0 | 0 | 0 |
|  | *Darwinopterus modularis* | 0 | 0 | ? | 3 | 0 | 0 | 0 | 3 | 0 | 1 | 0 | 1 | 0 | 1 | ? | 0 | 0 | 0 |
|  | *Wukongopterus lii* | 0 | 0 | ? | 3 | 0 | 0 | 0 | 3 | 0 | 1 | 0 | 1 | 0 | 1 | ? | 0 | 0 | 0 |
|  | *Pterorhynchus wellnhoferi* | 0 | 0 | ? | 3 | 0 | 0 | 0 | 3 | 0 | 1 | 0 | 1 | 0 | 1 | ? | 0 | 0 | 0 |
|  | *Batrachognathus volans* | 0 | 0 | ? | 3 | 0 | 0 | 0 | 3 | 0 | 1 | 0 | 0 | 0 | 1 | ? | 0 | 0 | 0 |
|  | *Jeholopterus ningchengensis* | 0 | 0 | ? | 3 | 0 | 0 | 0 | 3 | 0 | 1 | 0 | 0 | 0 | 1 | ? | 0 | 0 | 0 |
|  | *Anurognathus ammoni* | 0 | 0 | ? | 3 | 0 | 0 | 0 | 3 | 0 | 1 | 0 | 0 | 0 | 1 | ? | 0 | 0 | 0 |
|  | *Dendrorhynchoides curvidentatus* | 0 | 0 | ? | 3 | 0 | 0 | 0 | 3 | ? | 1 | 0 | 0 | 0 | 1 | ? | ? | 0 | 0 |

| Feeding category | Taxon/Character | 0 | 1 | 2 | 3 | 13 | 14 | 15 | 16 | 42 | 43 | 44 | 45 | 46 | 89 | 116 | 125 |
| --- | --- | --- | --- | --- | --- | --- | --- | --- | --- | --- | --- | --- | --- | --- | --- | --- | --- |
| Piscivorous, Jurassic Rhamphorhynchidae | *Dorygnathus banthensis* | 0.18 | 0.178 | 0.552 | 0.407 | 0.77 | 0.4 | 0.413 | 0.577 | 0 | 0 | 1 | 0 | ? | 0 | 1 | 0 |
|  | *Sericipterus wucaiwanensis* | 0.225 | 0.15 | ? | 0.323 | ? | 0.028 | ? | ? | 0 | 0 | 1 | 1 | 0 | 0 | ? | 0 |
|  | *Angustinaripterus longicephalus* | 0.219 | ? | 0.488 | 0.335 | 0.616 | 0.036 | 0.143 | ? | 0 | 0 | 1 | 1 | 0 | 0 | 1 | 0 |
|  | *Harpactognathus gentryii* | 0.186 | ? | ? | ? | ? | 0.028 | ? | ? | 2 | 0 | 1 | 1 | 0 | ? | 1 | ? |
|  | *Cacibupterx caribensis* | 0.139 | ? | ? | ? | ? | 0.04 | ? | ? | 0 | 0 | 1 | ? | ? | 0 | 1 | ? |
|  | *Rhamphorhynchus muensteri* | 0.184 | 0.209 | 0.298 | 0.375 | 0.698 | 0.036 | 0.563 | 0.691 | 0 | 0 | 1 | 0 | ? | 1 | 1 | 0 |
|  | *Qinglongopterus guoi* | ? | 0.069 | 0.728 | 0.36 | 0.779 | 0.028 | 0.429 | 0.626 | 0 | 0 | 1 | 0 | ? | ? | 1 | 0 |
|  | *Scaphognathus crassirostris* | 0.104 | 0.235 | 0.328 | 0.281 | 0.655 | 0.024 | 0.238 | 0.482 | 0 | 0 | 1 | 0 | ? | 0 | 1 | 0 |
| Piscivorous, Jurassic archaeopterodactyloids | *Gallodactylus canjuersensis* | 0.236 | 0.255 | 0.399 | 0.421 | ? | ? | 0.24 | ? | 0 | 0 | 0 | 0 | ? | 1 | 1 | 0 |
|  | *Cycnorhamphus suevicus* | 0.271 | 0.285 | 0.337 | 0.461 | ? | ? | 0.494 | ? | 2 | 0 | 0 | 0 | ? | 1 | 1 | 0 |
|  | *Ardeadactylus longicollum* | 0.375 | 0.411 | 0.427 | 0.547 | 0.567 | 0.054 | 0.561 | 0.388 | 0 | 0 | 1 | 0 | ? | 1 | 1 | 0 |
|  | *Pterodactylus kochi* | 0.417 | 0.423 | 0.36 | 0.502 | 0.568 | 0.076 | 0.528 | 0.457 | 0 | 0 | 1 | 0 | ? | 1 | 1 | 0 |
|  | *Pterodactylus antiquus* | 0.424 | 0.371 | 0.382 | 0.496 | ? | 0.082 | 0.559 | 0.405 | 0 | 0 | 1 | 0 | ? | 1 | 1 | 0 |
|  | *Germanodactylus cristatus* | 0.248 | 0.378 | 0.273 | 0.428 | 0.622 | 0.052 | 0.65 | 0.607 | 0 | 0 | 1 | 0 | ? | 1 | 1 | 0 |
|  | *Germanodactlus rhamphastinus* | 0.268 | 0.539 | 0.473 | 0.336 | 0.625 | 0.058 | 0.66 | 0.417 | 0 | 0 | 1 | 0 | ? | 1 | 1 | 0 |
| Piscivorous, Cretaceous dentate | *Anhanguera santanae* | 0.272 | 0.457 | 0.583 | 0.559 | 0.822 | 0.08 | 0.704 | 0.547 | 0 | 0 | 1 | 1 | 0 | 1 | 1 | 0 |
|  | *Anhanguera piscator* | 0.35 | 0.401 | 0.441 | 0.523 | 0.785 | 0.089 | 0.678 | 0.669 | 0 | 0 | 1 | 1 | 0 | 1 | 1 | 0 |
|  | *Anhanguera araripensis* | 0.374 | ? | ? | 0.584 | 0.795 | 0.096 | ? | ? | 0 | 0 | 1 | 1 | 0 | 1 | 1 | 0 |
|  | *Liaoningopterus gui* | 0.47 | ? | ? | ? | ? | 0.068 | 0.488 | ? | 0 | 0 | 1 | 1 | 0 | 1 | 1 | 0 |
|  | *Tropeognathus mesembrinus* | 0.319 | ? | 0.451 | 0.496 | 0.671 | 0.048 | 0.389 | 0.649 | 0 | 0 | 1 | 1 | 0 | 1 | 1 | 0 |
|  | *Ludodactylus sibbicki* | 0.266 | ? | 0.525 | 0.558 | 0.842 | 0.08 | 0.521 | 0.717 | 0 | ? | 1 | 1 | 0 | 1 | 1 | 0 |
|  | *Cearadactylus atrox* | 0.394 | ? | 0.443 | 0.467 | 0.484 | 0.058 | 0.39 | 0.345 | 0 | 0 | 1 | 1 | 0 | ? | 1 | 0 |
|  | *Guidraco venator* | 0.256 | ? | 1 | 0.598 | 0.771 | 0.088 | 0.761 | 0.769 | 0 | ? | 1 | ? | ? | 1 | 1 | 0 |
| Piscivorous, Cretaceous edentate | *Pteranodon longiceps* | 0.29 | ? | 0.487 | 0.768 | ? | ? | 0.919 | ? | 0 | 0 | 0 | 0 | ? | 1 | 1 | 0 |
|  | *Pteranodon sternbergi* | 0.416 | 0.698 | 0.567 | 0.799 | ? | ? | 1 | ? | 0 | 0 | 0 | 0 | ? | 1 | 1 | 0 |
|  | *Nyctosaurus gracilis* | 0.387 | 0.486 | 0.515 | 0.741 | ? | ? | 0.8 | ? | 0 | 0 | 1 | 0 | ? | 1 | 1 | 0 |
|  | *Muzquizopteryx coahuilensis* | 0.224 | ? | ? | ? | ? | ? | ? | ? | ? | ? | ? | ? | ? | 1 | 1 | ? |

| Feeding category | Taxon/Character | 135 | 136 | 137 | 138 | 139 | 140 | 141 | 142 | 143 | 144 | 145 | 146 | 147 | 149 | 150 | 152 | 155 | 156 |
| --- | --- | --- | --- | --- | --- | --- | --- | --- | --- | --- | --- | --- | --- | --- | --- | --- | --- | --- | --- |
| Piscivorous, Jurassic Rhamphorhynchidae | *Dorygnathus banthensis* | 0 | 0 | ? | 2 | 0 | 1 | 0 | 3 | 0 | 1 | 1 | 0 | 1 | 1 | ? | 0 | 1 | 0 |
|  | *Sericipterus wucaiwanensis* | 0 | 0 | ? | 2 | 2 | 1 | 1 | 3 | 0 | 1 | 1 | 1 | 1 | 1 | ? | 1 | ? | 0 |
|  | *Angustinaripterus longicephalus* | 0 | 0 | ? | 2 | 2 | 1 | 1 | 3 | 0 | 1 | 1 | 1 | 1 | 1 | ? | 1 | ? | 0 |
|  | *Harpactognathus gentryii* | 0 | 0 | ? | ? | ? | ? | 1 | 3 | 0 | ? | ? | ? | 1 | ? | ? | ? | ? | 0 |
|  | *Cacibupterx caribensis* | 0 | 0 | ? | ? | ? | ? | 0 | 3 | ? | ? | ? | ? | 1 | ? | ? | ? | ? | 0 |
|  | *Rhamphorhynchus muensteri* | 0 | 0 | ? | 2 | 2 | 1 | 0 | 3 | 0 | 1 | 1 | 0 | 2 | 1 | ? | 1 | 1 | 0 |
|  | *Qinglongopterus guoi* | 0 | 0 | ? | 2 | ? | 1 | 1 | 3 | 0 | 1 | 1 | 1 | 1 | 1 | ? | 1 | 1 | ? |
|  | *Scaphognathus crassirostris* | 0 | 0 | ? | 2 | 0 | 1 | 0 | 3 | 0 | 1 | 0 | 0 | 0 | 1 | ? | 0 | 0 | 0 |
| Piscivorous, Jurassic archaeopterodactyloids | *Gallodactylus canjuersensis* | ? | ? | ? | ? | ? | ? | ? | ? | ? | ? | ? | ? | ? | ? | ? | ? | ? | ? |
|  | *Cycnorhamphus suevicus* | 0 | 0 | ? | 2 | 0 | 1 | 0 | ? | 0 | 1 | 0 | 0 | ? | 1 | ? | 0 | ? | 0 |
|  | *Ardeadactylus longicollum* | 0 | 0 | ? | 3 | 0 | 0 | 0 | 3 | 0 | 1 | 0 | 0 | 1 | 1 | ? | 0 | 0 | 0 |
|  | *Pterodactylus kochi* | 0 | 0 | ? | 3 | 0 | 0 | 0 | 3 | 0 | 1 | 0 | 0 | 0 | 1 | ? | 0 | 0 | 0 |
|  | *Pterodactylus antiquus* | 0 | 0 | ? | 3 | 0 | 0 | 0 | 3 | 0 | 1 | 0 | 0 | 0 | 1 | ? | 0 | 0 | 0 |
|  | *Germanodactylus cristatus* | 0 | 0 | ? | 3 | 0 | 0 | 0 | 3 | 0 | 1 | 0 | 0 | 0 | 1 | ? | 1 | 1 | 0 |
|  | *Germanodactlus rhamphastinus* | 0 | 0 | ? | 3 | 0 | 0 | 0 | 3 | 0 | 1 | 0 | 0 | 0 | 1 | ? | 0 | 0 | 0 |
| Piscivorous, Cretaceous dentate | *Anhanguera santanae* | 0 | 1 | 2 | 5 | 1 | ? | 0 | 3 | 1 | 1 | 1 | 1 | 1 | 1 | ? | 0 | 0 | 1 |
|  | *Anhanguera piscator* | 0 | 1 | 2 | 5 | 1 | 1 | 0 | 3 | 1 | 1 | 1 | 1 | 1 | 1 | ? | 0 | 0 | 1 |
|  | *Anhanguera araripensis* | 0 | 1 | 2 | 5 | ? | ? | 0 | 3 | 1 | 1 | 1 | 1 | 1 | 1 | ? | 0 | ? | 1 |
|  | *Liaoningopterus gui* | 0 | 1 | 2 | 5 | 1 | 1 | 0 | 3 | 1 | 1 | 0 | 1 | 1 | 1 | ? | 0 | 0 | 1 |
|  | *Tropeognathus mesembrinus* | 0 | 1 | 2 | 5 | 1 | ? | 0 | 3 | 1 | 1 | 0 | 1 | 1 | 1 | ? | 0 | 0 | 1 |
|  | *Ludodactylus sibbicki* | 0 | 1 | 2 | 5 | 1 | 1 | 0 | 3 | 1 | 1 | 0 | 1 | 1 | 1 | ? | 0 | 0 | 1 |
|  | *Cearadactylus atrox* | 0 | 1 | 2 | 5 | 0 | 1 | 0 | 3 | 1 | 1 | 0 | 1 | 1 | 1 | ? | 0 | 0 | 1 |
|  | *Guidraco venator* | 0 | 1 | 2 | 5 | 0 | 1 | 0 | 3 | 1 | 1 | 0 | 1 | 1 | 1 | ? | 0 | 0 | 1 |
| Piscivorous, Cretaceous edentate | *Pteranodon longiceps* | 1 | ? | ? | ? | ? | ? | ? | ? | ? | ? | ? | ? | ? | ? | ? | ? | ? | 0 |
|  | *Pteranodon sternbergi* | 1 | ? | ? | ? | ? | ? | ? | ? | ? | ? | ? | ? | ? | ? | ? | ? | ? | ? |
|  | *Nyctosaurus gracilis* | 1 | ? | ? | ? | ? | ? | ? | ? | ? | ? | ? | ? | ? | ? | ? | ? | ? | 0 |
|  | *Muzquizopteryx coahuilensis* | 1 | ? | ? | ? | ? | ? | ? | ? | ? | ? | ? | ? | ? | ? | ? | ? | ? | 0 |

| Feeding category | Taxon/Character | 0 | 1 | 2 | 3 | 13 | 14 | 15 | 16 | 42 | 43 | 44 | 45 | 46 | 89 | 116 | 125 |
| --- | --- | --- | --- | --- | --- | --- | --- | --- | --- | --- | --- | --- | --- | --- | --- | --- | --- |
| Filter-feeding | *Boreopterus cuiae* | 0.391 | ? | 0.453 | 0.614 | 0.809 | 0.112 | 0.94 | 0.837 | 0 | ? | 1 | 0 | ? | 1 | 1 | 0 |
|  | *Zhenyuanopterus longiristris* | 0.604 | 0.491 | 0.557 | 0.611 | 1 | 0.172 | 0.801 | 0.974 | 0 | ? | 1 | 0 | ? | 1 | 1 | 0 |
|  | *Liaodactylus primus* | ? | ? | 0.322 | 0.542 | 0.662 | 0.152 | 0.398 | 0.55 | 0 | 0 | 1 | 0 | ? | 3 | 1 | 0 |
|  | *Gnathosaurus subulatus* | 1 | ? | ? | 0.555 | 0.686 | 0.14 | ? | ? | 2 | 0 | 1 | 1 | 1 | 1 | 1 | 0 |
|  | *Gnathosaurus macrurus* | ? | ? | ? | ? | ? | 0.12 | ? | ? | ? | 0 | ? | ? | 1 | ? | ? | 0 |
|  | *Plataleorhynchus streptophorodon* | ? | ? | ? | ? | ? | 0.124 | ? | ? | 2 | 0 | 1 | 1 | 0 | 1 | ? | ? |
|  | *Huanhepterus quingyangensis* | 0.42 | ? | ? | ? | ? | 0.1 | ? | ? | 0 | 0 | 1 | 1 | 0 | ? | 1 | 0 |
|  | *Moganopterus zhuiana* | 0.917 | ? | 0.566 | 1 | 0.338 | 0.064 | 0.407 | 0.231 | 0 | ? | 1 | 0 | ? | ? | ? | 0 |
|  | *Ctenochasma elegans* | 0.599 | 0.418 | 0.37 | 0.682 | 0.738 | 0.404 | 0.841 | 0.756 | 0 | 1 | 1 | 0 | ? | 1 | 1 | 0 |
|  | *Ctenochasma porocristata* | 0.888 | ? | ? | ? | ? | 0.408 | ? | ? | 0 | 1 | 1 | 0 | ? | 1 | 1 | 0 |
|  | *Pterodaustro guinazui* | 0.846 | 0.607 | 0.653 | 0.769 | 0.889 | 0.999 | 0.854 | 1 | 0 | 0 | 0 | 0 | ? | 1 | 1 | 0 |
|  | *Gegepterus changi* | 0.769 | 0.622 | 0.47 | 0.772 | 0.491 | 0.151 | ? | 0.372 | 0 | 0 | 0 | ? | ? | 1 | 1 | 0 |
|  | *Feilongus youngi* | 0.896 | ? | 0.327 | 0.554 | 0.315 | 0.074 | 0.592 | 0.256 | 0 | 0 | 1 | 0 | ? | 1 | 1 | 0 |
| Herbivorous | *Tupandactylus navigans* | 0.128 | ? | ? | 0.268 | ? | ? | ? | ? | 0 | 0 | 2 | 0 | ? | 1 | ? | ? |
|  | *Tupandactylus imperator* | 0.173 | ? | ? | 0.188 | ? | ? | 0.72 | ? | 0 | 0 | 2 | 0 | ? | 1 | 1 | 1 |
|  | *Tapejara wellnhoferi* | 0.074 | 0.163 | 0.304 | 0.234 | ? | ? | 0.608 | ? | 0 | 0 | 2 | 0 | ? | 1 | 1 | 1 |
|  | *Europejara olcadesorum* | ? | ? | ? | ? | ? | ? | 0.313 | ? | 0 | 0 | ? | ? | ? | ? | 1 | 0 |
|  | *"Huaxiapterus" benxiensis* | 0.158 | ? | 0.321 | 0.362 | ? | ? | 0.762 | ? | 0 | 0 | 2 | 0 | ? | 1 | 1 | 0 |
|  | *"Huaxiapterus" corollatus* | 0.186 | 0.252 | 0.458 | 0.33 | ? | ? | 0.832 | ? | 0 | 0 | 2 | 0 | ? | ? | 1 | 0 |
|  | *Eopteranodon lii* | 0.22 | 0.161 | 0.63 | 0.324 | ? | ? | 0.882 | ? | 0 | 0 | 2 | 0 | ? | 1 | ? | 0 |
|  | *Sinopterus gui* | 0.213 | 0.123 | 0.307 | 0.159 | ? | ? | 0.331 | ? | 0 | ? | 2 | 0 | ? | 1 | 1 | 0 |
|  | *Huaxiapterus jii* | 0.202 | 0.274 | 0.558 | 0.102 | ? | ? | 0.443 | ? | 0 | 0 | 2 | 0 | ? | 1 | 1 | 0 |
|  | *Sinopterus dongi* | 0.166 | 0.466 | 0.216 | 0.158 | ? | ? | 0.733 | ? | 0 | 0 | 2 | 0 | ? | 1 | 1 | 0 |
|  | *Nemicolopterus crypticus* | 0.165 | ? | 0.17 | 0.437 | ? | ? | 0.642 | ? | 0 | ? | 2 | 0 | ? | 1 | 1 | 0 |

| Feeding category | Taxon/Character | 135 | 136 | 137 | 138 | 139 | 140 | 141 | 142 | 143 | 144 | 145 | 146 | 147 | 149 | 150 | 152 | 155 | 156 |
| --- | --- | --- | --- | --- | --- | --- | --- | --- | --- | --- | --- | --- | --- | --- | --- | --- | --- | --- | --- |
| Filter-feeding | *Boreopterus cuiae* | 0 | 0 | ? | 3 | 0 | 1 | 0 | 3 | 0 | 1 | 0 | 0 | 1 | 1 | ? | 0 | 0 | 1 |
|  | *Zhenyuanopterus longiristris* | 0 | 0 | ? | 3 | 0 | 1 | 0 | 3 | 0 | 1 | 0 | 0 | 1 | 1 | ? | 0 | 0 | 1 |
|  | *Liaodactylus primus* | 0 | 0 | ? | 2 | 0 | 1 | 1 | 3 | 0 | 1 | 0 | 1 | 2 | 1 | ? | 0 | 0 | 0 |
|  | *Gnathosaurus subulatus* | 0 | 0 | ? | 2 | 0 | 1 | 1 | 3 | 0 | ? | 0 | 1 | 2 | 1 | ? | 0 | ? | 0 |
|  | *Gnathosaurus macrurus* | 0 | 0 | ? | ? | ? | ? | 1 | 3 | 0 | ? | ? | ? | 1 | ? | ? | ? | 0 | ? |
|  | *Plataleorhynchus streptophorodon* | 0 | ? | ? | ? | ? | ? | 1 | 3 | 0 | ? | ? | ? | 1 | ? | ? | 0 | 0 | 0 |
|  | *Huanhepterus quingyangensis* | 0 | 0 | ? | 2 | 0 | 1 | 0 | 3 | 0 | 1 | 0 | 1 | 1 | 1 | ? | 0 | 0 | 0 |
|  | *Moganopterus zhuiana* | 0 | 0 | ? | 2 | 0 | 1 | 0 | 3 | 0 | 1 | 0 | 0 | 0 | 1 | ? | 0 | 0 | 0 |
|  | *Ctenochasma elegans* | 0 | 0 | ? | 2 | 0 | 1 | 1 | 2 | 0 | 1 | 0 | 1 | 2 | 1 | ? | 0 | 0 | 0 |
|  | *Ctenochasma porocristata* | 0 | 0 | ? | 2 | 0 | 1 | 1 | 2 | ? | 1 | 0 | 1 | 2 | 1 | ? | 0 | 0 | 0 |
|  | *Pterodaustro guinazui* | 0 | 0 | ? | 2 | 0 | 1 | 0 | 0 | 0 | 2 | 0 | 1 | 0 | 1 | ? | 0 | 0 | 0 |
|  | *Gegepterus changi* | 0 | 0 | ? | 2 | 0 | 1 | 0 | 3 | ? | 1 | 0 | 1 | 2 | 1 | ? | ? | ? | 0 |
|  | *Feilongus youngi* | 0 | 0 | ? | 2 | 0 | 1 | 0 | 3 | 0 | 1 | 0 | 0 | 0 | 1 | ? | 0 | 0 | 0 |
| Herbivorous | *Tupandactylus navigans* | 1 | ? | ? | ? | ? | ? | ? | ? | ? | ? | ? | ? | ? | ? | ? | ? | ? | 0 |
|  | *Tupandactylus imperator* | 1 | ? | ? | ? | ? | ? | ? | ? | ? | ? | ? | ? | ? | ? | ? | ? | ? | 0 |
|  | *Tapejara wellnhoferi* | 1 | ? | ? | ? | ? | ? | ? | ? | ? | ? | ? | ? | ? | ? | ? | ? | ? | 0 |
|  | *Europejara olcadesorum* | 1 | ? | ? | ? | ? | ? | ? | ? | ? | ? | ? | ? | ? | ? | ? | ? | ? | 0 |
|  | *"Huaxiapterus" benxiensis* | 1 | ? | ? | ? | ? | ? | ? | ? | ? | ? | ? | ? | ? | ? | ? | ? | ? | 0 |
|  | *"Huaxiapterus" corollatus* | 1 | ? | ? | ? | ? | ? | ? | ? | ? | ? | ? | ? | ? | ? | ? | ? | ? | 0 |
|  | *Eopteranodon lii* | 1 | ? | ? | ? | ? | ? | ? | ? | ? | ? | ? | ? | ? | ? | ? | ? | ? | 0 |
|  | *Sinopterus gui* | 1 | ? | ? | ? | ? | ? | ? | ? | ? | ? | ? | ? | ? | ? | ? | ? | ? | 0 |
|  | *Huaxiapterus jii* | 1 | ? | ? | ? | ? | ? | ? | ? | ? | ? | ? | ? | ? | ? | ? | ? | ? | 0 |
|  | *Sinopterus dongi* | 1 | ? | ? | ? | ? | ? | ? | ? | ? | ? | ? | ? | ? | ? | ? | ? | ? | 0 |
|  | *Nemicolopterus crypticus* | 1 | ? | ? | ? | ? | ? | ? | ? | ? | ? | ? | ? | ? | ? | ? | ? | ? | 0 |

| Feeding category | Taxon/Character | 0 | 1 | 2 | 3 | 13 | 14 | 15 | 16 | 42 | 43 | 44 | 45 | 46 | 89 | 116 | 125 |
| --- | --- | --- | --- | --- | --- | --- | --- | --- | --- | --- | --- | --- | --- | --- | --- | --- | --- |
| Durophagous | *Dsungaripterus weii* | 0.239 | 0.497 | 0.337 | 0.431 | 0.419 | 0.044 | 0.575 | 0.606 | 0 | 0 | 0 | 0 | ? | 1 | 1 | 0 |
|  | *Domeykodactylus ceciliae* | ? | ? | ? | ? | ? | 0.064 | ? | ? | ? | 0 | ? | ? | ? | ? | 1 | 0 |
|  | *Noripterus pavus* | 0.186 | ? | 0.455 | 0.511 | 0.569 | 0.052 | 0.761 | 0.11 | 0 | 0 | 1 | 0 | ? | 1 | 1 | 0 |
|  | *Noripterus complicidens* | ? | ? | ? | ? | ? | ? | 0.028 | ? | ? | 0 | ? | ? | ? | ? | 1 | 0 |
| Omnivorous, dentate | *Tupuxuara leonardii* | 0.426 | 0.708 | 0.217 | 0.257 | ? | ? | 0.895 | ? | 0 | 0 | 1 | 0 | ? | 1 | 1 | 0 |
|  | *Thalassodromeus sethi* | 0.15 | ? | 0.523 | 0.431 | ? | ? | ? | ? | 0 | 0 | 1 | 0 | ? | 1 | 1 | 0 |
|  | *Jidapterus edentus* | 0.136 | 0.409 | 0.533 | 0.574 | ? | ? | 0.812 | ? | 0 | 0 | 1 | 0 | ? | 1 | 1 | 0 |
|  | *Shenzhoupterus chaoyangensis* | 0.136 | ? | 0 | 0.574 | ? | ? | 0.812 | ? | 0 | ? | 1 | 0 | ? | ? | 1 | 0 |
| Omnivorous, edentate | *Lonchodectes compressirostris* | 0.486 | ? | ? | ? | ? | ? | ? | ? | 0 | 0 | 1 | 0 | ? | ? | 1 | ? |
| Carnivorous, dentate | *Haopterus gracilis* | 0.2 | 0.374 | 0.396 | 0.465 | 0.647 | 0.052 | 0.66 | 0.678 | 0 | 0 | 1 | 0 | ? | 1 | 1 | 0 |
|  | *Hongshanopterus lacustris* | ? | ? | ? | 0.541 | 0.593 | 0.072 | ? | ? | 0 | 0 | 1 | 0 | ? | 1 | 1 | ? |
|  | *Nurhachius ignaciobritoi* | 0.376 | 0.407 | 0.545 | 0.328 | 0.401 | 0.046 | 0.392 | 0.131 | 0 | 0 | 1 | 0 | ? | 1 | 1 | 0 |
|  | *Liaoxipterus brachyognathus* | ? | ? | ? | ? | ? | 0.044 | 0.369 | 0.174 | ? | 1 | ? | ? | ? | ? | ? | 0 |
|  | *Istiodactylus sinensis* | 0.33 | 0.604 | 0.546 | 0.218 | 0.24 | 0.06 | 0.107 | 0.006 | 2 | 1 | 1 | 0 | ? | 1 | 1 | 0 |
|  | *Istiodactylus latidens* | 0.43 | 0.6 | 0.278 | 0.178 | 0 | 0.052 | 0.179 | 0 | 2 | 1 | 1 | 0 | ? | 1 | 1 | 0 |
|  | *Longchengopterus zhaoi* | 0.274 | ? | 0.469 | 0.495 | 0.391 | 0.048 | 0.453 | 0.317 | 0 | 0 | 1 | 0 | ? | ? | ? | 0 |
| Carnivorous, edentate | *Zhejiangopterus linhaiensis* | 0.347 | ? | 0.5 | 0.488 | ? | ? | 0.806 | ? | 0 | ? | 1 | 0 | ? | 1 | 1 | 0 |
|  | *Quetzalcoatlus northropi* | 0.526 | ? | ? | ? | ? | ? | 0.74 | ? | 0 | 0 | 1 | 0 | ? | 1 | 1 | 0 |

| Feeding category | Taxon/Character | 135 | 136 | 137 | 138 | 139 | 140 | 141 | 142 | 143 | 144 | 145 | 146 | 147 | 149 | 150 | 152 | 155 | 156 |
| --- | --- | --- | --- | --- | --- | --- | --- | --- | --- | --- | --- | --- | --- | --- | --- | --- | --- | --- | --- |
| Durophagous | *Dsungaripterus weii* | 0 | 0 | ? | 1 | 0 | 0 | 0 | 3 | 0 | 1 | 0 | 1 | 0 | 1 | ? | 1 | 1 | 0 |
|  | *Domeykodactylus ceciliae* | 0 | 0 | ? | 5 | ? | ? | 0 | 3 | 0 | ? | ? | ? | 0 | ? | ? | ? | 1 | ? |
|  | *Noripterus pavus* | 0 | 0 | ? | 4 | 0 | 0 | 0 | 3 | 0 | 1 | 0 | 1 | 0 | 1 | ? | 1 | 1 | 0 |
|  | *Noripterus complicidens* | 0 | 0 | ? | 4 | 0 | ? | 0 | 3 | 0 | ? | ? | 1 | ? | 1 | ? | ? | ? | ? |
| Omnivorous, dentate | *Tupuxuara leonardii* | 1 | ? | ? | ? | ? | ? | ? | ? | ? | ? | ? | ? | ? | ? | ? | ? | ? | 0 |
|  | *Thalassodromeus sethi* | 1 | ? | ? | ? | ? | ? | ? | ? | ? | ? | ? | ? | ? | ? | ? | ? | ? | 0 |
|  | *Jidapterus edentus* | 1 | ? | ? | ? | ? | ? | ? | ? | ? | ? | ? | ? | ? | ? | ? | ? | ? | 0 |
|  | *Shenzhoupterus chaoyangensis* | 1 | ? | ? | ? | ? | ? | ? | ? | ? | ? | ? | ? | ? | ? | ? | ? | ? | 0 |
| Omnivorous, edentate | *Lonchodectes compressirostris* | 0 | 0 | ? | 5 | ? | ? | 0 | 3 | 0 | ? | ? | ? | 0 | ? | ? | 0 | 0 | 0 |
| Carnivorous, dentate | *Haopterus gracilis* | 0 | 0 | ? | 3 | 0 | 0 | 0 | 3 | 0 | 1 | 0 | 0 | 0 | 1 | ? | 0 | 0 | 0 |
|  | *Hongshanopterus lacustris* | 0 | 0 | ? | 0 | 0 | 0 | 0 | 3 | 0 | ? | 0 | 0 | 0 | 1 | ? | 0 | ? | 0 |
|  | *Nurhachius ignaciobritoi* | 0 | 0 | ? | 4 | 0 | 0 | 0 | 3 | 0 | 1 | 0 | 1 | 0 | 1 | ? | 0 | 0 | 1 |
|  | *Liaoxipterus brachyognathus* | 0 | 0 | ? | 4 | 0 | 0 | 0 | 1 | 0 | ? | 0 | 1 | 0 | 1 | ? | ? | 0 | ? |
|  | *Istiodactylus sinensis* | 0 | 0 | ? | 4 | 3 | 0 | 0 | 1 | 0 | 1 | 0 | 1 | 0 | 1 | ? | 0 | 0 | 1 |
|  | *Istiodactylus latidens* | 0 | 0 | ? | 4 | 3 | 0 | 0 | 1 | 0 | 1 | 0 | 1 | 0 | 1 | ? | 0 | 0 | 1 |
|  | *Longchengopterus zhaoi* | 0 | 0 | ? | 4 | 0 | 0 | 0 | 3 | 0 | 1 | 0 | 1 | 0 | 1 | ? | 0 | 0 | 1 |
| Carnivorous, edentate | *Zhejiangopterus linhaiensis* | 1 | ? | ? | ? | ? | ? | ? | ? | ? | ? | ? | ? | ? | ? | ? | ? | ? | 0 |
|  | *Quetzalcoatlus northropi* | 1 | ? | ? | ? | ? | ? | ? | ? | ? | ? | ? | ? | ? | ? | ? | ? | ? | 0 |

Table S3. Results from pairwise PERMANOVA test for statistical significance of eco-morphological diversity of pterosaurs. Sequential Bonferroni-corrected P-value is given for each comparison. Results of all significant comparisons are marked with asterisk/asterisks (*P<0.05; **P<0.005; ***P<0.0005); results of non-significant comparisons are shown without asterisk.

|  | Insectivorous  (n = 20) | Jurassic piscivorous Rham. (n = 8) | Jurassic piscivorous, Arch. (n = 7) | Cretaceous piscivorous dentate (n = 8) | Cretaceous piscivorous edentate  (n = 4) | Filter-feeding (n = 13) | Herbivorous  (n = 11) | Durophagous  (n = 4) | Omnivorous  (n = 5) |
| --- | --- | --- | --- | --- | --- | --- | --- | --- | --- |
| Jurassic piscivorous rhamphorhynchids | 0.0001*** |  |  |  |  |  |  |  |  |
| Jurassic piscivorous archaeopterodactyloids | 0.0053* | 0.001** |  |  |  |  |  |  |  |
| Cretaceous piscivorous dentate | 0.0001*** | 0.0003*** | 0.0006** |  |  |  |  |  |  |
| Cretaceous piscivorous edentate | 0.0003*** | 0.0019** | 0.0051** | 0.0026** |  |  |  |  |  |
| Filter-feeding | 0.0001*** | 0.0018** | 0.0111* | 0.0001*** | 0.0006** |  |  |  |  |
| Herbivorous | 0.0001*** | 0.0001*** | 0.0001*** | 0.0001*** | 0.0028** | 0.0001*** |  |  |  |
| Durophagous | 0.0044** | 0.0161* | 0.1516 | 0.002** | 0.0296* | 0.0132* | 0.0007** |  |  |
| Omnivorous | 0.0001*** | 0.0019** | 0.0071** | 0.0005** | 0.3308 | 0.0004*** | 0.0025** | 0.0158* |  |
| Carnivorous dentate  (n = 7) | 0.0019** | 0.0002*** | 0.0102* | 0.0002*** | 0.003** | 0.0002*** | 0.0001*** | 0.0776 | 0.0015** |

**Appendix****. Taxon/Character Data Matrix for Phylogenetic Analysis**

The following text includes the morphological matrix and search parameters used in the phylogenetic analysis. Readers can copy and save the appendix as a separate text file that is executable in TNT ([24,25]-Goloboff et al. 2008a, b).

nstates cont;

mxram 600;

xread

224 113

&[continuous]

Euparkeria_capensis 0.185 0.041 0.648 0.037 0.000 0.000 0.679 0.454 ? ? 0.417 0.614 0.138 0.665 0.068 0.089 0.274 0.044 0.019 0.379 0.086 0.297 0.795 0.628 0.000 0.046 ? 0.042 0.086 0.695 ? ? ? ? 0.000 0.632 0.016 0.994 0.657

Ornithosuchus_longidens 0.094 0.098 0.566 0.044 0.396 ? 1.000 0.426 ? ? 0.539 0.453 ? 0.676 0.044 0.667 ? 0.096 0.026 0.051 0.094 0.191 0.531 1.000 0.135 0.000 ? 0.001 0.107 0.235 ? ? ? ? 0.023 0.669 0.000 0.994 0.833

Herrerasaurus_ischigualastensis 0.134 ? 0.797 0.011 0.071 0.187 0.584 0.573 ? ? 0.373 0.851 ? 0.726 0.076 0.704 0.297 0.063 ? 0.023 0.114 0.109 ? 0.788 0.061 0.051 ? 0.021 0.052 0.487 0.000 ? ? ? 0.078 1.000 0.064 1.000 0.933

Scleromochlus_taylori ? 0.130 0.634 0.161 0.082 ? 0.497 0.938 ? ? 0.991 1.000 0.116 0.524 0.044 0.073 0.265 0.046 0.000 0.701 0.128 0.219 1.000 0.526 0.350 0.075 ? 0.000 0.000 1.000 0.002 ? ? ? 0.238 0.754 0.208 0.994 0.830

Eudimorphodon_ranzii 0.166 0.189 0.475 0.280 0.399 0.416 0.079 0.000 ? ? 0.754 ? ? 0.814 0.120 0.043 0.704 0.010 0.076 0.731 ? ? 0.315 0.470 0.327 0.560 0.292 0.170 0.488 0.572 ? ? ? ? ? 0.246 ? ? ?

Eudimorphodon_rosenfeldi 0.077 0.164 0.345 0.280 0.505 0.574 0.208 0.386 ? ? 0.996 0.434 ? 0.663 0.066 0.255 0.762 0.012 0.077 0.777 0.837 0.978 0.250 0.449 0.387 0.461 0.248 0.114 0.426 0.465 0.407 0.410 0.682 0.618 0.132 0.118 0.511 0.918 0.625

Eudimorphodon_cromptonellus ? ? ? ? ? ? ? ? ? ? ? ? ? ? 0.048 ? ? ? ? ? ? 0.412 0.596 0.604 ? 0.243 ? 0.116 0.421 0.485 0.269 0.593 0.827 ? ? 0.339 0.175 0.678 1.000

Peteinosaurus_zambellii ? ? ? ? ? ? ? ? ? ? ? ? ? ? 0.148 ? ? ? ? ? ? ? ? ? ? ? ? ? ? 0.581 ? 0.341 ? 0.575 ? ? ? 0.864 0.490

Caviramus_schesaplanensis ? ? ? ? ? ? ? ? ? ? ? ? ? ? 0.068 0.117 ? ? ? ? ? ? ? ? ? ? ? ? ? ? ? ? ? ? ? ? ? ? ?

Raeticodactylus_filisurensis 0.130 0.143 0.513 0.286 0.273 0.278 0.445 0.479 ? ? 0.480 0.594 ? 0.644 0.078 0.229 0.505 0.028 0.110 0.856 ? ? ? ? 0.562 0.418 0.127 ? ? ? 0.382 0.431 0.727 ? ? 0.041 0.558 0.432 0.621

Austriadactylus_cristatus 0.206 0.140 0.374 0.167 0.467 0.270 0.289 0.306 ? ? 0.479 0.302 0.206 0.680 0.100 0.114 0.593 0.015 0.050 ? 0.460 0.494 0.336 0.529 0.385 0.482 ? 0.107 ? ? 0.334 0.487 0.742 0.623 ? ? ? ? ?

Preondactylus_buffarinii 0.163 0.445 0.497 0.112 0.673 0.520 0.499 0.341 ? ? 0.472 ? ? 0.795 0.070 0.037 0.520 0.024 0.048 0.445 1.000 0.513 ? 0.510 0.780 0.478 0.172 0.110 0.433 0.649 0.302 0.598 0.846 0.542 0.194 0.305 0.451 0.610 0.584

Dimorphodon_macronyx 0.177 0.288 0.534 0.203 1.000 0.144 0.494 0.080 ? ? 0.203 ? ? 0.732 0.124 0.116 0.658 0.018 0.094 0.684 0.245 0.790 0.322 0.412 0.379 0.416 0.102 0.108 0.548 0.756 0.312 0.546 0.933 0.742 0.222 0.235 0.529 0.430 0.406

Parapsicephalus_purdoni 0.204 ? ? ? ? 0.260 ? 0.531 ? ? 0.378 ? 0.154 ? ? ? ? ? ? ? ? ? ? ? ? ? ? ? ? ? ? ? ? ? ? ? ? ? ?

Campylognathoides_liasicus 0.153 0.118 0.443 0.245 0.649 0.250 0.136 0.455 ? ? 0.720 0.421 0.004 0.746 0.056 0.108 0.688 0.038 0.074 1.000 0.330 0.545 0.230 0.378 0.265 0.370 0.272 0.116 0.660 0.471 0.515 0.527 0.634 0.514 0.618 0.101 0.347 0.561 0.713

Campylognathoides_zitteli 0.090 0.000 0.609 0.337 0.466 0.389 0.216 0.489 ? ? 0.648 ? ? 0.724 0.066 0.177 0.632 0.033 0.026 0.708 0.229 1.000 0.425 0.500 0.162 0.202 0.195 0.101 0.344 0.559 0.828 0.507 0.517 0.387 0.653 ? 0.412 0.942 0.694

Sericipterus_wucaiwanensis 0.225 0.150 ? 0.323 ? ? ? ? ? ? ? 0.488 ? ? 0.028 ? ? 0.057 0.084 0.776 ? ? 0.136 0.714 0.210 ? ? ? ? ? 0.351 0.391 ? 0.673 ? ? ? ? ?

Angustinaripterus_longicephalus 0.219 ? 0.488 0.335 0.465 1.000 0.928 1.000 ? ? 0.506 ? ? 0.616 0.036 0.143 ? ? ? ? ? ? ? ? ? ? ? ? ? ? ? ? ? ? ? ? ? ? ?

Harpactognathus_gentryii 0.186 ? ? ? ? ? ? ? ? ? ? ? ? ? 0.028 ? ? ? ? ? ? ? ? ? ? ? ? ? ? ? ? ? ? ? ? ? ? ? ?

Cacibupteryx_caribensis 0.139 ? ? ? ? 0.246 ? 0.729 ? ? 0.310 ? 0.084 ? 0.040 ? ? ? ? ? ? ? ? ? ? ? ? ? ? ? ? ? ? ? ? ? ? ? ?

Rhamphorhynchus_muensteri 0.184 0.209 0.298 0.375 0.243 0.411 0.000 0.690 ? ? 0.625 0.324 0.031 0.698 0.036 0.563 0.691 0.032 0.067 0.753 0.262 0.701 0.199 0.494 0.243 0.742 0.136 0.150 0.611 0.549 0.715 0.410 0.554 0.622 0.582 0.172 0.530 0.406 0.825

Qinglongopterus_guoi ? 0.069 0.728 0.360 0.543 ? 0.602 ? ? ? ? ? ? 0.779 0.028 0.429 0.626 0.014 0.064 0.538 0.307 0.314 0.098 0.715 0.230 0.694 0.044 0.133 0.311 0.625 0.498 0.472 0.632 0.366 ? 0.043 0.360 0.586 0.636

Nesodactylus_hesperius ? ? ? ? ? ? ? ? ? ? ? ? ? ? ? ? ? 0.018 0.076 0.864 0.112 0.631 0.100 0.359 0.274 0.815 ? 0.155 0.453 0.784 0.834 ? ? ? ? ? ? ? ?

Dorygnathus_banthensis 0.180 0.178 0.552 0.407 0.665 0.534 0.401 0.310 ? ? 0.444 0.287 0.229 0.770 0.040 0.413 0.577 0.034 0.099 0.841 0.265 0.635 0.296 0.634 0.321 0.722 0.090 0.131 0.573 0.474 0.340 0.654 0.889 0.733 0.537 0.157 0.450 0.501 0.603

Scaphognathus_crassirostris 0.104 0.235 0.328 0.281 0.345 0.169 0.347 0.303 ? ? 0.487 0.537 ? 0.655 0.024 0.238 0.482 0.013 0.099 0.973 0.142 0.463 0.266 0.594 0.350 0.796 0.085 0.129 0.558 0.640 0.330 0.547 0.741 0.724 0.604 0.242 0.268 0.245 0.503

Sordes_pilosus 0.178 0.254 0.221 0.202 0.402 0.237 0.487 0.471 ? ? 0.530 0.642 0.040 0.584 0.026 0.380 0.373 0.000 0.085 0.905 0.231 0.397 0.183 0.624 0.540 0.740 0.110 0.096 0.593 0.631 0.292 0.539 0.730 0.521 0.696 0.121 0.496 0.276 0.487

Darwinopterus_modularis 0.277 1.000 0.422 0.374 ? ? ? ? 0.440 0.387 0.525 0.285 ? 0.573 0.054 0.259 0.487 0.068 0.090 0.505 0.496 0.738 0.149 0.687 0.417 0.578 0.438 0.164 0.543 0.798 0.325 0.600 0.915 0.836 0.659 0.210 0.443 ? 0.462

Wukongopterus_lii 0.232 ? 0.469 0.290 ? ? ? ? 0.529 ? ? ? ? 0.587 0.056 0.223 ? 0.069 ? ? ? 0.570 0.291 ? ? 0.739 0.005 0.161 ? 0.632 0.324 0.580 0.928 1.000 0.715 0.194 0.602 0.235 0.478

Pterorhynchus_wellnhoferi 0.120 0.601 0.582 0.347 ? ? ? ? 0.563 0.521 0.550 ? ? 0.610 0.038 0.207 0.396 0.036 0.190 0.410 0.371 0.756 ? 0.443 0.888 0.843 0.199 0.146 0.693 ? 0.303 0.699 0.836 0.561 0.637 0.134 0.284 0.225 ?

Changchengopterus_pani ? ? ? ? ? ? ? ? ? ? ? ? ? ? ? ? ? 0.010 0.029 0.288 0.089 0.338 0.240 0.788 0.188 0.537 0.521 0.165 0.541 ? 0.428 0.526 0.693 ? ? 0.136 0.305 ? ?

Batrachognathus_volans ? 0.113 0.682 0.000 ? ? ? ? 0.076 0.000 0.687 0.549 0.303 0.772 0.048 0.020 ? 0.010 0.056 0.575 ? ? 0.146 0.569 0.640 0.712 ? 0.069 ? 0.779 ? ? ? ? ? 0.000 0.675 0.160 0.616

Jeholopterus_ningchengensis 0.102 0.022 0.842 0.077 ? ? ? ? 0.185 0.007 0.472 ? 0.215 0.842 0.028 0.000 0.625 0.008 0.056 0.412 0.007 0.014 0.394 0.565 0.640 0.401 0.061 0.069 0.268 0.760 0.411 0.362 0.337 0.000 0.831 0.055 0.269 0.354 0.770

Anurognathus_ammoni 0.000 0.148 0.526 0.024 ? ? ? ? 0.078 0.036 0.554 0.668 0.240 0.579 0.030 0.178 0.213 0.025 0.119 0.279 0.029 0.016 0.239 0.588 0.689 0.548 0.041 0.054 0.491 0.596 0.470 0.275 0.171 ? 0.855 0.125 0.541 0.375 0.806

Dendrorhynchoides_curvidentatus ? 0.073 0.876 0.099 ? ? ? ? ? ? ? ? 0.226 ? ? 0.000 ? ? ? 0.418 0.085 0.012 0.220 0.675 0.751 0.423 0.000 0.062 0.337 0.790 0.452 0.298 0.332 ? ? 0.071 0.438 0.427 0.772

Kryptodrakon_progenitor ? ? ? ? ? ? ? ? ? ? ? ? ? ? ? ? ? ? ? ? ? ? ? ? ? ? ? 0.313 ? 0.752 ? ? ? ? ? ? ? ? ?

Gnathosaurus_subulatus 1.000 ? ? 0.555 ? ? ? ? 0.231 0.488 0.732 0.118 0.355 0.686 0.140 ? ? ? ? ? ? ? ? ? ? ? ? ? ? ? ? ? ? ? ? ? ? ? ?

Gnathosaurus_macrurus ? ? ? ? ? ? ? ? ? ? ? ? ? ? 0.120 ? ? 0.483 ? ? ? ? ? ? ? ? ? ? ? ? ? ? ? ? ? ? ? ? ?

Plataleorhynchus_streptophorodon ? ? ? ? ? ? ? ? ? ? ? ? ? ? 0.124 ? ? ? ? ? ? ? ? ? ? ? ? ? ? ? ? ? ? ? ? ? ? ? ?

Huanhepterus_quingyangensis 0.420 ? ? ? ? ? ? ? ? ? ? ? ? ? 0.100 ? ? 0.486 0.635 0.397 ? ? ? 0.617 0.603 0.747 ? 0.260 0.504 0.190 0.411 ? ? ? ? 0.226 ? ? ?

Moganopterus_zhuiana 0.917 ? 0.566 1.000 ? ? ? ? 0.130 1.000 0.405 ? ? 0.338 0.064 0.407 0.231 0.418 ? ? ? ? ? ? ? ? ? ? ? ? ? ? ? ? ? ? ? ? ?

Elanodactylus_prolatus ? ? ? ? ? ? ? ? ? ? ? ? ? ? ? ? ? 0.176 0.268 0.314 ? ? 0.289 0.561 0.584 0.246 ? 0.251 0.432 0.197 0.388 0.596 0.740 0.472 0.563 ? ? ? ?

Kepodactylus_insperatus ? ? ? ? ? ? ? ? ? ? ? ? ? ? ? ? ? 0.114 ? ? ? ? ? ? ? ? ? ? ? 0.000 ? ? ? ? ? 0.172 ? ? ?

Ctenochasma_elegans 0.599 0.418 0.370 0.682 ? ? ? ? 0.000 0.266 0.915 0.297 0.410 0.738 0.404 0.841 0.756 0.214 0.189 ? 0.018 0.040 0.247 0.497 0.561 0.364 0.598 0.320 0.971 0.067 0.388 0.388 0.420 0.442 0.927 0.171 0.523 0.202 0.593

Ctenochasma_porocristata 0.888 ? ? ? ? ? ? ? ? ? ? ? ? ? 0.408 ? ? ? ? ? ? ? ? ? ? ? ? ? ? ? ? ? ? ? ? ? ? ? ?

Pterodaustro_guinazui 0.846 0.607 0.653 0.769 ? ? ? ? 0.021 0.331 0.591 0.057 ? 0.889 0.999 0.854 1.000 0.225 0.214 0.213 0.068 0.187 0.149 0.487 0.537 0.550 ? 0.322 ? 0.356 0.447 0.440 0.454 0.383 ? 0.170 0.622 0.441 0.907

Eosipterus_yangi ? ? ? ? ? ? ? ? ? ? ? ? ? ? ? ? ? 0.245 ? ? ? ? ? 0.479 ? 0.295 ? 0.259 0.502 0.132 0.322 0.429 0.527 0.478 ? 0.018 0.751 ? 0.647

Beipiaopterus_chenianus ? ? ? ? ? ? ? ? ? ? ? ? ? ? ? ? ? 0.199 0.378 0.027 0.007 0.035 0.191 0.564 1.000 0.470 0.241 0.316 0.562 0.253 0.252 1.000 1.000 0.834 ? 0.059 1.000 0.264 0.525

Gegepterus_changae 0.769 0.622 0.470 0.772 ? ? ? ? 0.085 0.404 0.555 0.196 ? 0.491 0.151 ? 0.372 0.178 0.207 0.041 ? ? ? ? ? ? ? ? ? ? ? 0.450 ? ? ? ? ? ? ?

Boreopterus_cuiae 0.391 ? 0.453 0.614 ? ? ? ? 0.138 0.351 0.472 ? ? 0.809 0.112 0.940 0.837 0.079 ? ? ? 0.140 ? ? ? 0.511 0.443 0.370 ? 0.294 0.484 0.365 0.417 0.413 ? 0.305 0.140 ? 0.109

Feilongus_youngi 0.896 ? 0.327 0.554 ? ? ? ? 0.197 0.495 ? 0.075 ? 0.315 0.074 0.592 0.256 ? ? ? ? ? ? ? ? ? ? ? ? ? ? ? ? ? ? ? ? ? ?

Gallodactylus_canjuersensis 0.236 0.255 0.399 0.421 ? ? ? ? 0.362 0.268 0.812 0.349 ? ? ? 0.240 ? ? ? 0.225 ? ? 0.140 ? ? ? ? ? ? ? ? 0.337 0.311 ? 0.762 ? 0.457 0.284 0.324

Cycnorhamphus_suevicus 0.271 0.285 0.337 0.461 ? ? ? ? 0.218 0.248 1.000 0.429 ? ? ? 0.494 ? 0.122 0.151 0.284 0.011 0.086 0.167 0.553 0.380 0.429 0.922 0.523 0.571 0.312 0.596 0.287 0.306 0.290 0.662 0.391 0.625 0.162 0.230

Ardeadactylus_longicollum 0.375 0.411 0.427 0.547 ? ? ? ? 0.254 0.340 0.631 0.303 ? 0.567 0.054 0.561 0.388 0.258 0.298 0.492 ? ? 0.207 0.484 0.584 0.416 0.727 0.458 0.370 0.246 0.507 0.313 0.193 0.206 0.614 0.384 0.427 0.083 0.428

Pterodactylus_kochi 0.417 0.423 0.360 0.502 ? ? ? ? 0.143 0.261 0.772 0.295 ? 0.568 0.076 0.528 0.457 0.286 0.278 0.191 0.030 0.035 0.278 0.573 0.488 0.437 0.590 0.299 0.749 0.142 0.361 0.417 0.534 0.376 0.784 0.265 0.476 0.260 0.593

Pterodactylus_antiquus 0.424 0.371 0.382 0.496 ? ? ? ? 0.286 0.278 0.664 0.192 ? ? 0.082 0.559 0.405 0.261 0.294 0.356 0.002 0.058 0.359 0.655 0.397 0.499 0.547 0.303 0.520 0.266 0.378 0.406 0.542 0.471 0.984 0.285 0.476 0.245 0.555

Normannognathus_wellnhoferi 0.301 ? ? ? ? ? ? ? ? ? ? ? ? ? ? ? ? ? ? ? ? ? ? ? ? ? ? ? ? ? ? ? ? ? ? ? ? ? ?

Germanodactylus_cristatus 0.248 0.378 0.273 0.428 ? ? ? ? 0.318 0.175 0.511 0.202 ? 0.622 0.052 0.565 0.607 0.154 0.133 0.189 0.021 0.074 0.262 0.556 0.658 0.401 0.647 0.336 0.706 0.309 0.382 0.411 0.523 0.488 0.933 0.246 0.516 0.267 0.505

Germanodactylus_rhamphastinus 0.268 0.539 0.473 0.336 ? ? ? ? 0.395 0.247 0.451 0.090 ? 0.625 0.058 0.660 0.417 0.113 0.205 0.103 ? ? 0.255 0.634 0.538 0.563 ? 0.333 0.681 0.039 0.396 0.299 0.472 0.476 1.000 0.301 0.548 0.368 0.469

Haopterus_gracilis 0.200 0.374 0.396 0.465 ? ? ? ? 0.318 0.764 ? ? ? 0.647 0.052 0.660 0.678 0.026 0.113 0.063 ? ? 0.023 0.649 0.548 0.553 0.390 0.366 ? ? 0.588 0.277 0.352 0.138 ? ? ? ? ?

Anhanguera_santanae 0.272 0.457 0.583 0.559 ? ? ? ? 0.273 0.217 0.292 0.170 ? 0.822 0.080 0.704 0.547 0.072 0.145 0.248 ? ? 0.000 0.146 ? ? ? ? 0.552 0.309 ? ? ? ? 0.531 ? ? ? ?

Anhanguera_piscator 0.350 0.401 0.441 0.523 ? ? ? ? 0.230 0.222 0.992 0.150 ? 0.785 0.089 0.678 0.669 0.025 0.104 0.085 0.057 0.179 0.016 0.125 0.501 0.638 ? 0.305 0.671 0.376 ? ? ? ? ? 0.216 0.329 ? 0.214

Anhanguera_blittersdorffi 0.403 ? 0.442 0.555 ? ? ? ? 0.244 0.279 0.397 0.154 0.164 0.804 0.098 ? 0.673 ? ? ? ? ? ? ? ? ? ? ? ? ? ? ? ? ? ? ? ? ? ?

Anhanguera_araripensis 0.374 ? ? 0.584 ? ? ? ? 0.185 0.228 0.264 0.075 0.182 0.795 0.096 ? ? ? 0.145 ? ? ? ? ? ? ? ? ? ? 0.098 ? ? ? ? ? ? ? ? ?

Liaoningopterus_gui 0.470 ? ? ? ? ? ? ? ? ? ? ? ? ? 0.068 0.488 ? 0.080 ? ? ? ? ? ? ? ? ? ? ? ? ? ? ? ? ? ? ? ? ?

Tropeognathus_mesembrinus 0.319 ? 0.451 0.496 ? ? ? ? 0.341 0.302 0.269 0.084 0.127 0.671 0.048 0.389 0.649 ? ? ? ? ? ? ? ? ? ? ? ? ? ? ? ? ? ? ? ? ? ?

Coloborhynchus_clavirostris ? ? ? ? ? ? ? ? ? ? ? ? ? ? ? ? ? ? ? ? ? ? ? ? ? ? ? ? ? ? ? ? ? ? ? ? ? ? ?

Ornithocheirus_simus ? ? ? ? ? ? ? ? ? ? ? ? ? ? ? ? ? ? ? ? ? ? ? ? ? ? ? ? ? ? ? ? ? ? ? ? ? ? ?

Brasileodactylus_araripensis ? ? ? ? ? ? ? ? ? ? ? ? ? ? ? ? ? ? ? ? ? ? ? ? ? ? ? ? ? ? ? ? ? ? ? ? ? ? ?

Ludodactylus_sibbicki 0.266 ? 0.525 0.558 ? ? ? ? 0.249 0.205 0.393 0.092 ? 0.842 0.080 0.521 0.717 ? ? ? ? ? ? ? ? ? ? ? ? ? ? ? ? ? ? ? ? ? ?

Guidraco_venator 0.256 ? 1.000 0.598 ? ? ? ? 0.172 0.261 0.368 0.079 ? 0.771 0.088 0.761 0.769 0.018 ? ? ? ? ? ? ? ? ? ? ? ? ? ? ? ? ? ? ? ? ?

Zhenyuanopterus_longirostris 0.604 0.491 0.557 0.611 ? ? ? ? 0.207 0.355 0.595 0.067 ? 1.000 0.172 0.801 0.974 0.111 0.193 ? 0.035 0.029 0.295 0.202 0.593 0.376 0.487 0.337 0.689 0.038 0.479 0.243 0.289 0.316 ? 0.277 0.099 0.368 0.000

Cearadactylus_atrox 0.394 ? 0.443 0.467 ? ? ? ? 0.276 ? ? ? ? 0.484 0.058 0.390 0.345 ? ? ? ? ? ? ? ? ? ? ? ? ? ? ? ? ? ? ? ? ? ?

Hongshanopterus_lacustris ? ? ? 0.541 ? ? ? ? 0.213 ? ? 0.245 0.315 0.593 0.072 ? ? 0.021 ? ? ? ? ? ? ? ? ? ? ? ? ? ? ? ? ? ? ? ? ?

Lonchodectes_compressirostris 0.486 ? ? ? ? ? ? ? ? ? ? ? ? ? ? ? ? ? ? ? ? ? ? ? ? ? ? ? ? ? ? ? ? ? ? ? ? ? ?

Nurhachius_ignaciobritoi 0.376 0.407 0.545 0.328 ? ? ? ? 1.000 0.940 ? ? 0.631 0.401 0.046 0.392 0.131 0.092 0.124 0.341 ? ? 0.045 0.092 0.437 0.752 0.392 0.340 0.444 0.106 0.536 0.281 ? ? ? 0.333 0.362 ? 0.056

Liaoxipterus_brachyognathus ? ? ? ? ? ? ? ? ? ? ? ? ? ? 0.044 0.369 0.174 ? ? ? ? ? ? ? ? ? ? ? ? ? ? ? ? ? ? ? ? ? ?

Istiodactylus_sinensis 0.330 0.604 0.546 0.218 ? ? ? ? 0.715 0.424 0.593 0.153 ? 0.240 0.060 0.107 0.006 0.071 0.121 0.202 ? ? 0.078 0.149 0.726 0.843 ? 0.391 0.527 0.270 0.574 0.362 0.417 ? ? 0.431 0.256 ? ?

Istiodactylus_latidens 0.430 0.600 0.278 0.178 ? ? ? ? 0.422 0.322 0.500 0.120 0.456 0.000 0.052 0.179 0.000 ? 0.140 0.126 ? ? 0.028 0.097 0.478 0.826 0.209 ? 0.410 ? ? ? ? ? ? 0.210 ? ? ?

Longchengopterus_zhaoi 0.274 ? 0.469 0.495 ? ? ? ? 0.308 0.313 0.690 0.187 1.000 0.391 0.048 0.453 0.317 0.082 ? ? ? ? 0.025 0.134 ? 0.858 0.406 0.354 0.465 0.321 0.598 0.310 0.342 ? ? 0.376 ? ? ?

Pteranodon_longiceps 0.290 ? 0.487 0.768 ? ? ? ? 0.136 0.139 0.235 0.115 0.396 ? ? 0.919 ? 0.071 ? ? ? ? 0.086 0.389 ? 0.505 0.281 0.715 0.356 0.263 0.698 0.301 ? ? ? 0.273 0.521 0.188 0.472

Pteranodon_sternbergi 0.416 0.698 0.567 0.799 ? ? ? ? 0.073 0.127 0.203 0.033 ? ? ? 1.000 ? 0.085 0.163 0.027 ? 0.000 0.096 ? 0.465 0.576 0.147 0.736 ? 0.242 0.830 0.186 0.247 ? ? 0.256 0.512 ? ?

Nyctosaurus_gracilis 0.387 0.486 0.515 0.741 ? ? ? ? 0.198 0.215 0.482 0.254 0.137 ? ? 0.800 ? 0.078 0.141 0.114 0.000 0.039 0.079 0.413 0.351 0.923 1.000 1.000 0.488 0.189 1.000 0.274 0.163 0.180 0.565 0.213 0.445 0.161 0.373

Nyctosaurus_lamegoi ? ? ? ? ? ? ? ? ? ? ? ? ? ? ? ? ? ? ? ? ? ? ? ? ? ? ? ? ? ? ? ? ? ? ? ? ? ? ?

Muzquizopteryx_coahuilensis 0.224 ? ? ? ? ? ? ? ? 0.207 0.912 ? ? ? ? ? ? ? ? ? ? ? ? 0.507 ? 0.510 0.728 ? ? ? ? ? ? ? ? 0.254 0.484 ? 0.207

Tupandactylus_navigans 0.128 ? ? 0.268 ? ? ? ? 0.484 0.137 0.238 0.365 0.106 ? ? ? ? ? ? ? ? ? ? ? ? ? ? ? ? ? ? ? ? ? ? ? ? ? ?

Tupandactylus_imperator 0.173 ? ? 0.188 ? ? ? ? 0.770 0.206 0.243 0.000 ? ? ? 0.720 ? ? ? ? ? ? ? ? ? ? ? ? ? ? ? ? ? ? ? ? ? ? ?

Bakonydraco_galaczi ? ? ? ? ? ? ? ? ? ? ? ? ? ? ? 0.704 ? ? ? ? ? ? ? ? ? ? ? ? ? ? ? ? ? ? ? ? ? ? ?

Tapejara_wellnhoferi 0.074 0.163 0.304 0.234 ? ? ? ? 0.487 0.119 0.329 0.499 0.000 ? ? 0.608 ? 0.115 0.089 0.000 0.031 0.050 0.242 0.528 0.157 0.504 0.564 0.407 0.819 0.065 0.543 0.309 0.350 0.232 0.668 0.379 0.365 0.388 0.408

Europejara_olcadesorum ? ? ? ? ? ? ? ? ? ? ? ? ? ? ? 0.313 ? ? ? ? ? ? ? ? ? ? ? ? ? ? ? ? ? ? ? ? ? ? ?

Huaxiapterus_benxiensis 0.158 ? 0.321 0.362 ? ? ? ? 0.478 0.242 ? 0.360 ? ? ? 0.762 ? 0.126 ? ? ? ? ? 0.611 ? 1.000 0.584 0.703 0.769 0.011 0.803 0.220 0.263 ? ? 0.877 0.465 ? 0.264

Huaxiapterus_corollatus 0.186 0.252 0.458 0.330 ? ? ? ? 0.456 0.019 ? ? ? ? ? 0.832 ? 0.042 0.090 ? ? ? 0.217 0.547 0.433 0.591 0.638 0.632 0.542 0.235 0.615 0.128 0.128 0.016 ? 0.514 0.585 0.151 0.202

Eopteranodon_lii 0.220 0.161 0.630 0.324 ? ? ? ? 0.447 0.145 ? ? ? ? ? 0.882 ? 0.103 0.118 0.091 ? ? ? 0.662 0.227 0.606 0.644 0.492 0.560 0.256 0.568 0.252 0.229 0.153 0.683 0.413 ? ? ?

Sinopterus_gui 0.213 0.123 0.307 0.159 ? ? ? ? 0.409 0.206 ? ? ? ? ? 0.331 ? 0.079 0.041 0.702 ? ? 0.248 0.388 0.109 0.575 0.342 ? ? ? 0.434 ? ? ? 0.760 0.367 0.615 0.363 0.327

Huaxiapterus_jii 0.202 0.274 0.558 0.102 ? ? ? ? 0.555 0.242 ? ? ? ? ? 0.443 ? 0.160 0.178 0.173 ? ? ? 0.595 0.468 0.593 0.597 0.538 0.643 0.276 0.574 0.264 0.273 0.091 ? 0.475 0.495 0.234 0.296

Sinopterus_dongi 0.166 0.466 0.216 0.158 ? ? ? ? 0.773 0.240 0.310 ? ? ? ? 0.733 ? 0.096 0.159 0.151 ? ? 0.164 0.568 0.503 0.568 0.562 0.507 0.776 0.092 0.558 0.242 0.233 0.055 0.680 0.445 0.499 0.061 0.221

Nemicolopterus_crypticus 0.165 ? 0.170 0.437 ? ? ? ? 0.177 0.122 0.163 0.326 ? ? ? 0.642 ? 0.063 ? ? ? 0.034 0.321 0.462 ? 0.439 ? ? 0.734 ? 0.411 0.397 0.421 0.230 ? 0.316 0.525 0.279 0.315

Bennettazhia_oregonensis ? ? ? ? ? ? ? ? ? ? ? ? ? ? ? ? ? ? ? 0.063 ? ? ? ? 0.449 ? ? ? ? ? ? ? ? ? ? ? ? ? ?

Dsungaripterus_weii 0.239 0.497 0.337 0.431 ? ? ? ? 0.271 0.137 0.533 0.345 0.032 0.419 0.044 0.575 0.606 0.021 0.157 0.125 0.037 0.093 0.343 0.558 0.357 0.709 ? 0.600 0.749 0.305 0.676 0.249 0.497 0.365 ? 0.605 0.635 ? 0.113

Domeykodactylus_ceciliae ? ? ? ? ? ? ? ? ? ? ? ? ? ? 0.064 ? ? ? ? ? ? ? ? ? ? ? ? ? ? ? ? ? ? ? ? ? ? ? ?

Noripterus_parvus 0.186 ? 0.455 0.511 ? ? ? ? 0.296 0.129 0.468 0.295 ? 0.569 0.052 0.761 0.110 0.031 0.072 0.499 ? ? ? 0.569 0.179 0.529 0.646 0.623 0.591 0.303 0.574 0.330 0.369 0.291 ? 0.472 0.587 0.173 ?

Noripterus_complicidens ? ? ? ? ? ? ? ? ? ? ? ? ? ? 0.028 ? ? 0.146 0.143 0.682 0.037 0.199 ? 0.667 0.400 0.488 ? 0.607 0.314 0.302 0.645 0.196 ? ? 0.264 0.385 0.867 0.075 0.203

Tupuxuara_longicristatus 0.139 ? ? ? ? ? ? ? ? ? ? ? ? ? ? ? ? ? ? ? ? ? ? ? ? ? ? ? ? ? ? ? ? ? ? ? ? ? ?

Tupuxuara_leonardii 0.426 0.708 0.217 0.257 ? ? ? ? 0.398 0.210 0.123 0.028 0.166 ? ? 0.895 ? 0.053 0.148 0.091 ? ? 0.230 0.410 0.364 0.386 0.611 0.508 0.774 0.248 0.615 0.101 0.121 ? ? 0.479 0.434 0.226 0.255

Thalassodromeus_sethi 0.150 ? 0.523 0.431 ? ? ? ? 0.513 0.150 0.007 0.085 ? ? ? ? ? ? ? ? ? ? ? ? ? ? ? ? ? ? ? ? ? ? ? ? ? ? ?

Chaoyangopterus_zhangi 0.226 ? ? ? ? ? ? ? ? ? ? ? ? ? ? ? ? 0.182 0.233 0.121 ? ? 0.196 0.530 0.488 0.488 ? 0.640 0.512 0.121 0.610 0.145 0.101 0.056 0.348 0.605 0.624 0.286 0.297

Jidapterus_edentus 0.136 0.409 0.533 0.574 ? ? ? ? 0.423 0.107 ? ? ? ? ? 0.812 ? 0.107 0.187 0.212 ? ? 0.198 0.401 0.437 0.544 0.751 0.621 0.474 0.296 0.599 0.190 0.143 0.035 ? 0.472 0.567 0.320 0.297

Eoazhdarcho_liaoxiensis ? ? ? ? ? ? ? ? ? ? ? ? ? ? ? 0.617 ? 0.167 0.176 0.153 ? ? 0.197 0.000 0.512 0.476 0.499 0.478 0.464 0.226 0.555 0.259 0.230 0.093 ? 0.310 0.748 ? ?

Shenzhoupterus_chaoyangensis 0.136 ? 0.000 0.574 ? ? ? ? 0.423 0.107 0.000 ? ? ? ? 0.812 ? 0.163 ? ? ? ? 0.130 ? ? 0.695 ? 0.695 ? 0.222 0.627 0.165 0.172 0.061 ? 0.683 0.454 ? 0.384

Zhejiangopterus_linhaiensis 0.347 ? 0.500 0.488 ? ? ? ? 0.354 0.207 0.400 0.159 ? ? ? 0.806 ? 0.544 0.507 0.340 ? ? 0.236 0.514 0.564 0.633 0.764 0.678 1.000 0.093 0.544 0.260 ? ? ? 0.600 0.644 0.000 0.266

Azhdarcho_lancicollis 0.423 ? ? ? ? ? ? ? ? ? ? ? ? ? ? ? ? 0.284 ? 0.143 ? ? ? ? ? ? ? ? ? ? ? ? ? ? ? ? ? ? ?

Arambourgiania_philadelphiae ? ? ? ? ? ? ? ? ? ? ? ? ? ? ? ? ? 1.000 ? ? ? ? ? ? ? ? ? ? ? ? ? ? ? ? ? ? ? ? ?

Quetzalcoatlus_northropi 0.526 ? ? ? ? ? ? ? ? 0.302 ? ? 0.181 ? ? 0.740 ? 0.789 1.000 0.002 ? ? 0.199 0.297 ? 0.542 0.673 0.753 0.521 0.285 0.629 0.000 0.000 ? ? ? 0.304 0.010 ?

Liaodactylus_primus ? ? 0.322 0.542 ? ? ? ? 0.256 ? ? ? ? 0.662 0.152 0.398 0.55 ? ? ? ? ? ? ? ? ? ? ? ? ? ? ? ? ? ? ? ? ? ?

&[numeric]

Euparkeria_capensis 1 0 - 0 0 1 0 - 0 0 0 0 0 0 0 0 0 0 0 0 0 0 0 - - - - - - - 0 0 0 0 0 0 0 1 0 0 - - 0 0 0 0 - 0 0 0 0 0 0 0 - 0 0 0 0 0 0 0 0 0 0 0 0 0 0 0 - - 0 0 0 0 0 1 0 0 0 0 - 0 0 0 0 0 0 0 0 1 0 0 - 0 0 0 - 0 1 0 0 0 0 0 0 0 0 0 0 0 0 0 1 0 0 0 0 0 0 0 0 0 0 0 0 0 0 0 0 0 0 0 0 0 0 0 0 0 0 0 0 0 0 0 0 0 0 0 0 0 0 0 0 0 - 0 - 0 - - - - 0 0 0 0 0 ? ? ? 0 0 0 - 0 0 0 0 0 0 0 1 0

Ornithosuchus_longidens 1 0 - 0 ? 1 0 - ? 0 0 0 0 0 0 2 0 0 1 0 1 ? 0 - - - - - - - 0 0 0 0 0 0 0 0 ? 0 - - 1 0 1 0 - 0 0 0 0 0 0 0 - 0 0 0 0 0 0 0 0 ? ? ? 0 ? ? ? ? ? 0 ? ? 0 0 1 0 0 0 0 - 0 0 0 0 ? ? ? ? 1 0 0 - 0 0 0 - 0 0 0 0 0 0 0 0 0 0 0 0 0 0 0 1 0 0 0 ? 0 0 0 0 0 0 0 0 0 [01] 0 0 0 0 0 0 0 0 0 0 0 0 1 0 0 0 0 0 0 0 0 0 0 1 0 0 0 - 0 - ? - - - - 0 0 0 ? ? 0 ? ? 0 0 0 - 0 0 0 0 0 1 ? 0 ?

Herrerasaurus_ischigualastensis 1 0 - 0 0 1 0 - 0 0 0 0 1 0 0 0 0 0 0 0 0 0 0 - - - - - - - 0 0 0 2 0 0 0 0 0 0 - - 1 0 0 0 - 0 0 0 0 0 0 0 - 0 0 0 0 0 0 0 0 0 0 0 0 0 ? ? ? ? 0 ? ? 0 0 1 0 0 0 0 - 0 0 0 0 0 0 ? ? 2 0 0 - 0 0 0 - 0 0 0 0 0 0 0 0 0 0 0 0 0 0 0 1 0 0 0 0 0 0 0 0 0 0 0 ? 0 0 0 0 0 0 0 0 0 0 0 ? ? ? ? ? ? ? ? 0 0 0 1 0 0 1 0 1 0 - 0 - 0 - - - - 0 0 1 1 0 0 0 0 0 0 0 - 0 0 0 0 0 0 0 3 0

Scleromochlus_taylori 1 1 - 0 0 ? 0 - ? ? 0 1 0 0 0 0 0 ? 0 0 1 0 0 - - - - - - - 0 1 0 0 0 0 0 0 ? 0 - - 1 ? ? 0 - 0 ? 0 2 0 ? ? - 0 ? ? ? 0 0 1 ? 0 0 0 0 ? ? ? ? ? 0 0 ? 0 ? 1 0 0 0 0 - 0 0 ? 0 0 0 ? ? 1 2 0 - 0 0 0 ? ? ? 0 0 0 ? ? ? ? ? 0 ? ? ? 0 ? ? ? 0 0 ? ? ? 1 0 0 0 0 ? ? 0 0 0 0 0 0 0 0 0 0 0 ? ? ? ? ? ? ? ? 0 1 ? ? 1 0 0 0 ? ? ? ? - - - - 0 1 ? ? ? ? ? ? 0 0 0 - 1 ? 0 0 ? 0 0 3 -

Eudimorphodon_ranzii 1 1 - 0 0 1 0 - 0 1 0 1 0 0 0 0 0 1 1 0 2 0 0 - - - - - - - 0 1 0 3 0 0 0 0 0 0 - - 1 0 0 0 - 0 0 0 2 1 0 0 - 0 0 0 0 0 0 1 0 ? ? ? 0 ? 0 0 - - 0 1 ? 0 0 2 0 0 0 1 0 0 0 0 0 0 1 0 0 1 1 0 - 0 0 1 0 1 1 0 0 0 1 1 0 0 1 0 0 4 2 0 1 0 1 0 ? 1 0 ? ? ? 0 0 0 ? 0 0 ? ? ? 0 ? 0 0 0 0 2 0 1 0 1 0 1 0 0 1 1 0 0 1 0 1 0 - ? ? 0 ? ? 1 0 0 0 0 1 0 ? ? ? ? ? ? 0 1 ? 0 ? 0 0 ? ? ?

Eudimorphodon_rosenfeldi 1 1 - 0 ? 1 0 - 0 1 0 1 0 0 0 0 0 1 1 0 2 0 0 - - - - - - - 0 1 0 3 0 0 0 0 0 0 - - 1 ? 0 0 - 0 0 0 2 1 0 0 - 0 0 0 0 ? 0 1 0 0 0 1 0 0 ? ? ? ? ? ? ? 0 0 1 0 0 0 1 0 0 0 0 0 0 1 ? ? 1 1 0 - 0 0 1 0 1 0 0 0 0 1 1 0 0 1 0 0 4 2 0 1 0 1 0 ? 1 0 ? 0 0 0 0 0 0 ? 0 ? 0 0 0 0 ? 0 0 0 1 0 1 0 0 0 1 ? 0 1 1 0 ? 1 0 1 0 - 0 0 0 ? 0 0 0 0 0 0 1 0 0 0 0 0 0 0 ? 1 ? ? ? 0 ? 0 2 0

Eudimorphodon_cromptonellus ? ? ? ? ? ? ? ? 0 ? ? 1 0 0 ? ? ? ? ? ? ? ? ? ? ? ? ? ? ? ? 0 1 ? 2 0 0 ? 0 0 ? ? ? ? ? 0 ? ? 0 ? ? ? ? 0 ? - 0 ? ? ? 0 ? ? ? ? ? ? ? ? ? ? ? ? ? ? ? ? 0 ? ? 0 ? ? ? ? ? 0 0 ? ? ? ? ? ? ? ? ? 0 ? ? 1 0 0 0 0 ? 1 0 ? [01] 0 0 4 2 ? 1 ? ? ? ? ? 0 ? ? ? 0 ? ? ? ? ? 0 ? 0 0 ? 0 0 ? 0 1 0 1 ? 0 0 1 ? ? 1 1 0 0 1 0 1 ? ? ? ? 0 ? ? ? ? 0 ? 0 1 0 ? ? 0 ? ? ? ? 1 0 0 0 0 0 ? 2 0

Peteinosaurus_zambellii ? ? ? ? ? ? ? - ? ? ? ? ? ? ? ? ? ? ? ? ? ? ? ? ? ? ? ? ? ? ? ? ? ? ? ? ? ? ? ? ? ? ? ? ? ? ? ? ? ? ? ? 0 ? ? ? ? ? ? ? ? ? ? ? ? ? ? ? ? ? ? ? ? ? ? 0 0 ? ? 0 0 0 - 0 ? ? 0 0 0 ? ? ? ? 0 - 0 0 1 0 1 0 0 0 0 1 ? 0 0 0 0 0 2 2 ? ? 0 0 0 ? ? ? ? ? ? ? ? ? ? ? ? ? ? ? ? ? ? 0 ? ? ? ? ? ? ? ? ? ? ? ? ? ? ? ? ? ? ? ? ? ? ? ? ? ? ? ? 0 0 1 0 0 0 0 0 0 0 ? ? ? ? ? ? ? ? ? 0

Caviramus_schesaplanensis ? ? ? ? ? ? 0 - ? ? ? 1 ? ? ? ? ? ? ? ? ? ? ? ? ? ? ? ? ? ? ? ? ? ? ? ? ? ? ? ? ? ? ? ? ? ? ? ? ? ? ? ? ? ? ? ? ? ? ? ? ? ? ? ? ? ? ? ? ? ? ? ? ? ? ? ? 0 1 0 0 0 1 1 1 1 1 1 0 0 ? ? 0 2 0 - 0 0 ? ? 1 0 0 0 0 ? ? 0 ? [01] 0 0 3 2 ? ? 0 1 0 ? ? ? ? ? ? ? ? ? ? ? ? ? ? ? ? ? ? ? ? ? ? ? ? ? ? ? ? ? ? ? ? ? ? ? ? ? ? ? ? ? ? ? ? ? ? ? ? ? ? ? ? ? ? ? ? ? ? ? ? ? ? ? ? ? ? ?

Raeticodactylus_filisurensis 1 1 - 0 ? 1 0 - 0 1 0 1 1 0 0 0 0 ? 0 0 2 0 1 0 1 0 1 0 0 0 0 1 0 2 0 0 0 0 0 0 - - ? 0 0 0 - 0 0 0 2 0 0 0 - 0 0 0 0 0 0 1 0 ? ? ? 0 ? ? ? ? ? 0 1 ? ? 0 1 1 0 0 1 1 1 1 1 1 0 0 ? ? 0 2 0 - 0 0 1 2 1 1 0 0 0 1 0 0 0 1 0 0 3 2 0 1 0 1 0 ? 1 0 ? 0 0 0 0 ? ? ? 0 0 ? ? ? ? ? ? 0 ? ? ? ? ? ? ? ? 0 ? 1 1 ? ? 1 0 1 0 - 0 ? ? ? ? 0 ? 0 0 0 ? ? 0 ? 0 ? ? ? ? 1 0 0 0 ? 0 ? ? 0

Austriadactylus_cristatus 1 1 - 0 0 1 0 - 0 1 ? 1 0 0 0 0 0 ? 0 0 1 ? 1 0 1 0 2 0 0 0 0 1 0 1 0 0 1 0 0 0 - - 1 0 0 0 - 0 0 ? 1 0 0 0 - 0 0 0 0 0 ? ? 0 ? ? 1 0 ? ? ? ? ? 0 ? ? ? ? 2 0 0 0 0 - 0 0 0 0 0 0 ? ? 1 2 0 - 0 0 1 [01] 1 1 0 0 0 1 0 0 0 0 0 0 1 1 0 1 0 0 0 ? ? ? ? 0 0 0 0 0 0 0 0 ? 0 0 0 0 0 0 ? 0 1 ? ? ? 0 0 1 ? ? 1 1 ? ? ? 0 1 ? 0 ? ? ? ? ? ? 0 0 0 0 1 ? ? ? 0 0 ? 0 ? 1 ? ? ? ? ? ? ? ?

Preondactylus_buffarinii 1 1 - 0 ? 1 0 - 0 1 0 1 0 0 0 ? 0 ? ? 0 1 0 0 - - - - - - - 0 1 0 1 0 0 1 0 0 0 - - ? ? 0 ? ? ? ? ? 1 0 0 0 - 0 0 0 0 ? ? 1 ? ? ? ? ? ? ? ? ? ? ? ? ? ? 0 1 0 0 0 0 - 0 0 0 0 0 0 ? ? 1 1 0 - ? 0 1 0 1 ? 0 0 0 1 0 0 0 0 0 0 1 1 0 1 0 0 0 0 ? ? ? ? 0 ? 0 0 ? ? ? 0 ? 0 0 0 ? 0 ? 0 1 ? ? ? ? ? ? ? ? 1 1 ? ? ? 0 1 0 - ? ? ? ? ? 0 0 0 0 0 1 0 0 ? 0 0 0 0 0 1 ? 0 0 ? 0 0 2 0

Dimorphodon_macronyx 0 1 - 0 ? 1 0 - 0 1 1 1 0 0 0 2 0 3 0 0 2 1 0 - - - - - - - 0 1 1 1 0 0 1 0 0 0 - - 1 1 0 0 - 0 0 0 1 0 0 0 - 0 0 0 0 0 0 1 ? ? ? 1 0 ? ? ? ? ? 0 1 ? ? 0 1 1 0 0 0 - 0 0 0 0 0 0 0 0 1 3 1 0 1 0 0 - 0 1 0 0 1 1 0 0 0 0 0 1 - - 0 1 0 0 0 0 ? 1 ? ? ? 0 0 0 ? ? 0 0 0 1 0 ? 0 0 0 1 2 ? ? ? 0 0 ? 0 0 1 1 0 0 1 0 2 0 - ? ? 0 0 0 2 0 0 0 0 1 0 0 0 0 1 1 0 1 1 0 0 0 0 0 0 2 0

Parapsicephalus_purdoni 0 ? ? 0 0 ? ? ? 0 1 1 1 0 0 0 2 0 3 0 0 2 1 0 - - - - - - - 0 1 1 2 0 0 1 0 0 0 - - 1 0 0 0 - 0 0 0 1 0 0 0 - 0 0 0 0 0 0 1 0 0 0 1 0 0 0 0 - - 0 1 0 0 1 ? ? ? ? ? ? ? ? ? ? ? ? ? ? ? ? ? ? ? 0 ? ? ? ? ? 0 1 ? ? ? ? [01] 0 ? ? ? ? ? ? ? 0 ? ? ? ? ? ? ? ? ? ? ? ? ? ? ? ? ? ? ? ? ? ? ? ? ? ? ? ? ? ? ? ? ? ? ? ? ? ? ? ? ? ? ? ? ? ? ? ? ? ? ? ? ? ? ? ? ? ? ? ? ? ? ? ? ? ? ?

Campylognathoides_liasicus 1 1 - 0 0 1 0 - 0 1 1 1 0 0 0 0 0 2 1 0 2 1 0 - - - - - - - 0 2 0 2 0 0 0 0 0 0 - - 1 0 0 0 - 0 0 0 2 0 0 0 - 0 0 1 0 0 0 0 0 0 0 1 0 0 0 0 - - 0 1 ? 0 1 2 1 0 0 0 - 0 0 0 0 1 - 0 0 1 3 0 - 1 0 0 - 0 0 0 0 1 1 1 0 0 0 0 1 - - 0 0 0 1 0 0 1 0 ? 0 0 0 0 0 0 0 0 0 0 1 0 0 0 0 0 0 2 1 1 1 0 0 1 0 1 1 1 ? ? 1 0 1 0 - 0 0 1 ? 0 2 0 0 0 0 1 0 0 0 1 1 1 0 1 1 0 1 1 0 0 0 2 1

Campylognathoides_zitteli 1 1 - 0 0 1 0 - 0 1 1 1 0 0 0 0 0 ? ? 0 2 1 0 - - - - - - - 0 2 0 2 0 0 0 0 0 0 - - 1 0 0 0 - 0 0 ? ? 0 0 0 - 0 0 1 0 0 ? 0 ? ? ? ? 0 ? 0 ? ? ? ? ? ? 0 1 2 1 0 0 0 - 0 0 0 0 1 - 0 0 1 3 0 - 1 0 0 - 0 0 0 0 1 1 1 0 0 0 0 1 - - 0 0 0 1 0 ? ? ? ? 0 0 0 0 0 ? ? 0 0 0 1 0 ? 0 0 0 0 2 ? 1 1 0 0 1 ? 1 1 1 ? ? 1 0 1 0 - 0 0 1 ? ? 2 0 0 0 0 1 0 ? 0 1 1 1 0 1 1 0 1 1 ? 0 0 2 1

Sericipterus_wucaiwanensis 1 3 1 0 0 1 1 0 1 1 1 1 1 0 1 ? ? ? ? 0 ? 1 1 0 0 1 ? 0 0 1 0 1 ? 2 0 0 0 0 ? 0 - - ? ? ? 0 - 0 ? 0 2 0 0 1 - ? 0 0 ? ? 1 1 ? 0 ? ? 0 0 ? 0 - - ? ? ? 1 1 ? ? ? ? ? ? 1 0 0 0 1 - 0 0 ? 3 ? ? 1 0 0 - 2 2 1 1 3 0 1 1 1 1 1 1 - - 1 0 ? ? 0 ? 1 0 0 0 0 0 0 ? ? ? ? 0 ? ? ? ? 0 0 ? 0 2 ? ? ? ? ? ? 0 0 1 1 ? 0 1 0 3 0 - 0 0 ? ? ? ? ? ? ? ? ? ? ? 0 2 1 2 0 ? ? ? ? ? ? ? ? ? ?

Angustinaripterus_longicephalus 1 3 1 0 0 1 1 0 1 1 1 1 1 0 1 0 0 2 1 0 2 ? 1 0 0 1 ? 0 0 1 0 1 0 2 0 0 0 0 0 0 - - 1 ? 0 ? ? 0 0 0 2 1 0 1 - 0 0 0 0 0 1 1 ? ? ? ? ? ? ? ? ? ? 0 1 ? ? 1 1 1 0 0 0 - 1 0 0 0 1 - ? ? 1 3 0 - 1 0 0 - 2 2 1 1 3 0 1 1 1 1 1 1 - - 1 0 0 ? 0 ? ? ? ? ? ? ? ? ? ? ? ? ? ? ? ? ? ? ? ? ? ? ? ? ? ? ? ? ? ? ? ? ? ? ? ? ? ? ? ? ? ? ? ? ? ? ? ? ? ? ? ? ? ? ? ? ? ? ? ? ? ? ? ? ? ? ?

Harpactognathus_gentryii 1 3 0 2 0 1 1 0 1 1 ? 1 1 0 ? ? ? ? ? 0 ? ? 1 0 0 1 ? 0 0 1 0 0 ? 2 0 0 ? 0 ? ? ? ? ? ? ? ? ? ? ? ? ? ? ? ? - ? ? ? ? ? ? ? ? ? ? ? ? ? 0 0 - - 0 1 ? ? 1 ? ? ? ? ? ? ? ? ? ? ? ? ? ? ? ? ? ? ? 0 0 - ? ? ? 1 3 0 ? ? ? [01] 1 ? ? ? 1 0 ? ? 0 ? ? ? ? ? ? ? ? ? ? ? ? ? ? ? ? ? ? ? ? ? ? ? ? ? ? ? ? ? ? ? ? ? ? ? ? ? ? ? ? ? ? ? ? ? ? ? ? ? ? ? ? ? ? ? ? ? ? ? ? ? ? ? ? ? ? ?

Cacibupteryx_caribensis 1 ? ? 0 0 1 ? ? 1 1 1 0 1 0 1 0 0 2 0 0 ? 1 0 - - - - - - - 0 0 0 2 0 0 0 0 0 0 - - 0 0 0 0 - 0 0 0 1 0 0 1 - 0 0 0 0 0 0 ? 0 0 0 1 0 0 0 0 - - 0 1 0 ? 1 ? ? ? ? ? ? ? ? ? ? ? ? ? ? ? ? ? ? ? 0 0 - ? ? ? 0 3 ? ? ? ? [01] 0 ? ? ? ? 0 0 ? 0 ? ? ? ? ? ? ? ? ? ? ? ? ? ? ? ? ? ? ? ? ? ? ? ? ? ? ? ? ? ? ? ? ? ? ? ? ? ? ? ? ? 0 ? ? ? ? ? ? ? ? ? ? ? ? ? ? ? ? ? ? ? ? ? ? ? ? ?

Rhamphorhynchus_muensteri 1 3 0 0 0 1 0 - 1 1 1 0 1 0 1 0 0 2 1 0 2 1 0 - - - - - - - 0 0 0 2 0 0 0 0 0 0 - - 0 0 0 0 - 0 0 1 2 2 0 1 - 0 0 0 0 0 - 1 0 0 0 1 0 0 0 0 - - 0 1 0 1 1 0 2 0 0 0 - 1 0 0 0 1 - 0 0 1 3 0 - 1 0 0 - 2 2 1 0 3 0 1 1 0 2 0 1 - - 1 0 0 1 0 0 1 1 0 0 0 0 0 0 0 0 0 0 0 1 0 0 0 0 0 0 2 1 1 0 0 0 [12] 0 0 1 1 0 0 1 0 6 0 - 0 1 0 0 0 2 0 0 1 0 1 0 1 0 1 1 2 0 1 1 0 1 1 0 0 1 2 [12]

Qinglongopterus_guoi ? 3 ? 0 0 1 0 - 1 ? ? 1 1 0 1 0 0 2 ? ? 2 ? ? ? ? ? ? ? ? ? ? 0 ? 2 0 0 0 0 ? ? ? ? ? ? ? ? ? 0 ? ? 2 0 0 ? - 0 0 ? 0 0 ? ? ? 0 0 1 ? ? ? ? ? ? 0 ? ? ? 1 1 1 0 0 ? ? 1 ? 0 0 1 - ? ? 1 3 0 - 1 0 0 - 2 ? 1 1 3 0 1 1 1 1 1 1 - - 1 0 0 1 ? ? 1 0 ? ? ? 0 0 0 ? 0 0 ? 0 1 0 0 0 0 0 0 2 ? ? ? 0 0 ? ? ? 1 1 ? ? 1 0 3 0 - ? 0 0 ? ? 3 ? 0 1 0 1 ? 1 0 0 1 ? 0 1 1 ? 1 1 0 0 1 2 1

Nesodactylus_hesperius ? ? ? ? ? ? ? ? ? ? ? ? ? ? ? ? ? ? ? ? ? ? ? ? ? ? ? ? ? ? ? ? ? ? ? ? ? ? ? ? ? ? ? ? ? ? ? ? ? ? 1 ? 0 ? ? ? ? ? ? ? ? ? ? ? ? ? ? ? ? ? ? ? ? ? ? ? ? ? ? ? ? ? ? ? ? ? ? ? ? ? ? ? ? ? ? ? ? ? ? ? ? ? ? ? ? ? ? ? ? ? ? ? ? ? ? ? ? ? ? ? 1 ? 0 0 0 0 ? ? ? ? ? 0 1 0 ? 0 0 ? 0 2 ? 1 1 0 0 2 0 ? 1 1 ? ? 1 0 6 0 - 0 1 0 0 ? ? 0 0 1 0 1 0 ? 0 1 ? ? ? 0 ? ? 1 ? ? ? ? ? ?

Dorygnathus_banthensis 1 1 - 0 0 1 0 - 1 1 1 1 1 0 1 0 0 2 1 0 2 1 0 - - - - - - - 0 0 0 2 0 0 0 0 0 0 - - 1 0 0 0 - 0 0 0 2 0 0 1 - 0 0 0 0 0 0 1 0 0 0 1 0 0 0 0 - - 0 1 0 1 1 1 1 0 0 1 0 1 0 0 0 1 - 0 0 1 3 0 - 1 0 0 - 2 0 1 0 3 0 1 1 0 1 0 1 - - 0 0 0 1 0 0 1 0 ? 0 0 0 0 0 0 0 0 0 0 1 0 0 0 0 0 0 2 1 1 0 0 0 3 0 1 1 1 0 0 1 0 3 0 - 0 1 0 0 0 3 0 0 1 0 1 0 1 0 0 1 2 0 1 1 0 1 1 0 0 1 2 2

Scaphognathus_crassirostris 1 1 - 0 0 1 0 - 0 1 1 1 1 0 0 0 0 2 1 0 2 1 0 - - - - - - - 0 1 0 2 0 0 0 0 0 0 - - 1 0 0 0 - 0 0 0 2 0 0 1 - 0 0 0 0 0 0 1 0 ? ? ? 0 ? ? ? ? ? 0 1 0 0 1 1 1 0 0 0 - 1 0 0 0 1 - ? ? 1 3 0 - 1 0 0 - 2 0 1 0 3 0 1 0 0 0 0 1 - - 0 0 0 0 0 0 ? ? 0 0 0 0 0 0 0 0 0 0 0 1 0 0 0 0 0 0 2 1 1 ? 0 0 2 0 0 1 1 ? ? 1 0 3 0 - 0 1 0 0 0 2 0 0 1 0 1 0 1 0 0 1 2 ? 1 1 0 1 1 0 0 1 2 2

Sordes_pilosus 1 1 - 0 0 1 0 - 0 1 1 1 0 0 0 0 0 2 1 0 2 1 0 - - - - - - - 0 1 0 2 0 0 1 0 0 0 - - 1 0 0 0 - 0 0 1 2 1 0 1 - 0 0 0 1 0 - 1 0 0 0 1 0 0 0 0 - - 0 1 0 0 1 1 0 0 0 0 - 1 0 0 0 1 - 0 0 1 3 0 - 1 0 0 - 3 0 0 0 3 0 1 0 0 0 0 1 - - 0 0 0 0 0 0 1 1 ? 0 0 0 0 0 0 0 0 0 0 1 0 ? 0 0 0 0 2 ? ? ? 0 0 ? 0 1 1 1 ? ? 1 0 0 0 - 0 0 0 0 ? [45] 0 0 1 0 1 0 1 0 0 1 2 0 1 1 0 1 1 0 0 1 2 2

Darwinopterus_modularis 1 1 - 0 0 1 0 - 0 1 1 - - 1 0 0 0 4 1 0 - 1 1 1 1 2 2 0 0 0 0 - - - 0 0 1 0 0 0 - - ? 0 0 0 - 0 0 1 2 1 0 1 0 0 0 0 1 0 - 1 0 ? ? ? 0 ? 0 1 0 0 0 1 ? ? 1 1 0 0 0 0 - 1 0 0 0 1 - ? ? 1 0 0 - 1 0 0 - 3 0 0 0 3 0 1 0 1 0 1 1 - - 0 0 0 0 0 1 ? ? ? 1 0 1 0 0 ? 0 0 ? 0 1 0 0 0 0 0 0 2 ? 1 ? 0 0 2 0 ? 1 1 ? ? ? 0 0 0 - ? ? ? ? 0 4 ? 0 1 0 ? ? 0 ? 0 1 ? 0 1 1 ? 1 1 0 0 1 2 2

Wukongopterus_lii 1 1 - 0 0 1 0 - 0 1 ? - - 1 ? ? ? ? ? 0 - ? ? ? ? ? ? ? ? ? 0 - - - ? ? ? ? ? ? ? ? ? ? ? ? ? ? ? ? 2 ? ? ? 0 ? ? ? ? ? ? 1 ? ? ? ? ? ? ? ? ? ? 0 1 ? ? 1 1 0 0 0 0 - 1 0 0 0 1 - ? ? 1 0 0 - 1 0 0 - 3 0 0 0 3 0 1 0 1 0 1 1 - - 0 0 0 0 0 ? ? 0 ? 1 0 ? 0 0 1 0 0 0 0 1 0 0 0 0 0 0 2 ? ? ? 0 0 2 ? 0 1 1 ? ? 1 0 0 ? ? ? ? 0 ? ? 4 ? 0 1 0 1 0 0 ? ? ? ? ? ? 1 ? 1 1 ? 0 1 2 2

Pterorhynchus_wellnhoferi 1 1 - 0 0 1 0 - 0 1 1 - - 1 0 0 0 4 1 0 - ? 1 1 0 1 1 0 0 0 0 - - - 0 0 1 0 0 0 - - ? 0 0 0 - 0 0 ? 2 2 0 1 0 0 0 0 1 0 ? 1 0 ? ? ? 0 ? ? ? ? ? ? ? ? ? 1 1 0 0 0 0 - 1 0 0 0 1 - ? ? 1 0 0 - 1 0 0 - 3 0 0 0 3 0 1 0 1 0 0 1 - - 0 0 0 0 0 1 ? 0 ? ? ? 0 0 ? ? ? ? 0 0 1 0 0 ? 0 ? ? ? ? ? ? ? ? ? ? ? 1 1 ? ? 1 0 0 0 - ? ? ? ? ? 0 0 0 1 0 1 0 ? ? 0 1 2 0 ? 1 ? ? ? 0 ? ? 2 ?

Changchengopterus_pani ? ? ? ? ? ? ? ? ? ? ? ? ? ? ? ? ? ? ? ? ? ? ? ? ? ? ? ? ? ? ? ? ? ? ? ? ? ? ? ? ? ? ? ? ? ? ? ? ? ? ? ? ? ? ? ? ? ? ? ? ? ? ? ? ? ? ? ? ? ? ? ? ? ? ? ? ? ? ? ? ? ? ? ? ? ? ? ? ? ? ? ? ? ? ? ? ? ? ? ? ? ? ? ? ? ? ? ? ? ? ? ? ? ? ? ? ? ? ? ? 0 ? 1 0 0 0 0 1 ? ? ? 0 0 0 ? 0 0 ? 2 2 ? ? ? 0 0 ? 0 ? 1 1 ? ? ? 0 0 0 - ? ? ? ? ? 4 0 0 1 0 1 ? ? 0 0 ? ? ? ? 1 ? ? ? ? ? ? 2 1

Batrachognathus_volans 0 1 - 1 2 1 0 - 0 0 ? - - 1 0 0 0 0 1 1 - 1 0 - - - - - - - 0 - - - 1 - - - ? 0 - - 1 ? 0 0 - 0 0 ? 0 0 1 ? 0 0 0 1 1 0 ? ? ? ? ? ? 0 ? - - - - 1 0 1 0 1 1 0 1 0 0 - 0 0 2 0 1 - 0 0 2 2 0 - 1 0 0 - 3 0 0 0 3 0 1 0 0 0 0 1 - - 0 0 0 0 0 ? ? ? ? ? ? 0 0 1 ? ? 0 0 ? ? ? ? 0 0 0 0 2 0 1 0 0 0 2 0 0 1 1 ? ? 1 0 4 0 - 0 ? ? ? ? ? ? 0 1 0 1 0 1 ? ? ? ? ? ? 1 ? 1 ? ? 0 0 ? ?

Jeholopterus_ningchengensis 0 1 - 1 2 1 0 - 0 0 ? - - 1 0 0 0 0 1 1 - 1 0 - - - - - - - 0 - - - 1 - - - ? 0 - - 1 0 0 0 - 0 0 ? 0 0 1 ? 0 0 0 1 ? 0 ? ? ? ? ? ? 0 ? - - - - 1 0 1 ? 1 1 0 1 0 0 - ? 0 2 0 ? ? ? ? 2 2 ? ? ? 0 0 - 3 0 0 0 3 0 1 0 0 0 0 1 - - 0 0 0 0 0 ? ? ? ? 1 0 ? ? 1 1 0 0 ? 1 0 0 0 0 0 0 0 2 ? ? ? 0 0 ? ? 0 1 1 0 ? 1 0 4 0 - 0 0 ? ? ? 5 0 0 1 0 1 0 1 0 0 1 ? ? 1 1 ? 1 1 ? 0 0 2 0

Anurognathus_ammoni 0 1 - 1 2 1 0 - 0 0 ? - - 1 0 0 0 0 1 1 - 1 0 - - - - - - - 0 - - - 1 - - - 0 0 - - 1 0 0 0 - 0 0 ? 0 0 1 ? 0 0 0 1 1 0 ? ? ? ? ? ? 0 0 - - - - 1 0 1 0 1 1 0 1 0 0 - 0 0 2 0 1 - 0 0 2 2 0 - 1 0 0 - 3 0 0 0 3 0 1 0 0 0 0 1 - - 0 0 0 0 0 1 ? ? ? ? ? ? ? 1 ? 0 0 0 1 0 0 0 0 0 0 0 2 ? ? ? ? ? ? ? 0 1 1 ? ? 1 0 4 0 - ? ? 0 ? ? 5 0 0 1 0 1 0 1 ? ? 1 1 0 ? 1 0 1 1 0 0 0 2 0

Dendrorhynchoides_curvidentatus 0 1 - 1 2 1 0 - 0 0 ? ? ? ? ? ? ? ? ? 1 ? ? 0 - - - - - - - 0 ? ? ? ? ? ? ? ? ? ? ? ? ? ? ? ? ? ? ? ? 0 1 ? ? ? ? ? ? ? ? ? ? 0 0 1 ? ? - - - - 1 0 1 0 ? 1 0 1 0 0 - 0 0 2 0 ? ? ? ? ? ? 0 - ? 0 0 - 3 0 0 0 3 ? 1 0 0 0 0 1 - - ? ? ? 0 0 ? ? ? ? 1 0 ? ? 1 ? ? 0 ? 1 0 0 0 0 0 0 0 2 ? ? ? 0 0 ? ? 0 1 1 0 ? 1 0 0 0 - ? ? 0 ? ? 5 0 0 1 0 1 ? 1 ? 0 ? 1 ? ? 1 ? ? ? 0 ? 0 2 1

Kryptodrakon_progenitor ? ? ? ? ? ? ? ? ? ? ? ? ? ? ? ? ? ? ? ? ? ? ? ? ? ? ? ? ? ? ? ? ? ? ? ? ? ? ? ? ? ? ? ? ? ? ? ? ? ? ? ? ? ? ? ? ? ? ? ? ? ? ? ? ? ? ? ? ? ? ? ? ? ? ? ? ? ? ? ? ? ? ? ? ? ? ? ? ? ? ? ? ? ? ? ? ? ? ? ? ? ? ? ? ? ? ? ? ? ? ? ? ? ? ? ? ? ? ? ? ? ? ? ? ? ? ? ? ? ? ? ? ? ? ? ? ? ? ? 2 ? ? ? ? ? ? 0 ? ? 1 ? ? ? 0 ? ? ? ? ? 0 0 0 ? 1 0 ? 0 0 0 ? 0 0 ? ? ? ? ? ? ? ? ? ? ? ? ?

Gnathosaurus_subulatus 1 1 - 2 0 1 1 1 0 1 1 - - 1 0 0 0 4 1 0 - 1 1 1 0 3 1 0 0 0 0 - - - 0 0 1 0 0 0 - - 1 0 0 0 - 1 1 1 3 2 0 1 0 0 0 0 1 0 - 1 1 1 1 0 0 ? ? 1 1 0 0 1 1 ? 1 1 0 2 1 0 - 1 0 0 0 1 - ? ? ? ? 0 - 1 0 0 - 2 0 1 1 3 0 ? 0 1 2 0 1 - - 0 0 0 ? 0 ? ? ? ? ? ? ? ? ? ? ? ? ? ? ? ? ? ? ? ? ? ? ? ? ? ? ? ? ? ? ? ? ? ? ? ? ? ? ? ? ? ? ? ? ? ? ? ? ? ? ? ? ? ? ? ? ? ? ? ? ? ? ? ? ? ? ?

Gnathosaurus_macrurus ? ? ? ? 0 ? ? 1 ? ? ? ? ? ? ? ? ? ? ? ? ? ? ? ? ? ? ? ? ? ? ? ? ? ? ? ? ? ? ? ? ? ? ? ? ? ? ? ? ? ? ? ? ? ? ? ? ? ? ? ? ? ? ? ? ? ? ? ? ? ? 1 ? ? ? ? ? ? 1 0 2 1 0 - 1 0 ? 0 1 - 1 0 ? ? ? ? ? 0 0 - ? ? ? 1 3 0 ? ? ? 1 0 ? ? ? ? ? 0 0 ? ? 0 1 ? 2 2 1 0 1 ? ? ? ? ? ? ? ? ? ? ? ? ? ? ? ? ? ? ? ? ? ? ? ? ? ? ? ? ? ? ? ? ? ? ? ? ? ? ? ? ? ? ? ? ? ? ? ? ? ? ? ? ? ? ? ? ? ?

Plataleorhynchus_streptophorodon 1 1 - 2 0 1 1 0 ? 1 ? - - 1 ? ? ? ? ? ? - ? ? ? ? ? ? ? ? ? ? ? ? ? ? ? ? ? ? ? ? ? ? ? ? ? ? ? ? 1 ? ? ? ? ? ? ? ? ? ? - ? ? ? ? ? ? ? 1 0 - - 0 ? ? ? ? 1 ? ? ? ? ? ? ? ? ? ? ? ? ? ? ? ? ? ? 0 ? ? ? ? ? 1 3 0 ? ? ? 1 0 ? ? ? 0 0 0 0 0 ? ? ? ? ? ? ? ? ? ? ? ? ? ? ? ? ? ? ? ? ? ? ? ? ? ? ? ? ? ? ? ? ? ? ? ? ? ? ? ? ? ? ? ? ? ? ? ? ? ? ? ? ? ? ? ? ? ? ? ? ? ? ? ? ? ? ?

Huanhepterus_quingyangensis 1 1 - 0 0 1 1 0 ? ? ? ? ? ? ? ? ? ? ? ? ? ? 1 1 0 3 ? 0 0 0 ? ? ? ? ? ? ? ? ? ? ? ? ? ? ? ? ? ? ? ? ? ? ? ? ? ? ? ? ? ? ? ? ? ? ? ? ? ? ? ? ? ? ? ? ? ? 1 1 0 0 1 0 - 1 0 0 0 ? ? ? ? ? ? 0 - ? 0 0 - 2 0 1 0 3 0 1 0 1 1 0 1 - - 0 0 0 0 0 ? ? ? ? 2 2 ? 0 1 1 0 0 0 ? 0 ? ? ? 0 ? ? 2 ? ? ? ? ? 2 ? ? 1 ? ? ? 1 0 5 ? ? ? ? ? ? ? ? ? 0 ? 1 ? ? ? ? ? ? ? ? ? 1 ? 1 ? ? 0 ? ? ?

Moganopterus_zhuiana 1 1 - 0 ? 1 0 - 1 1 1 - - 1 0 0 0 4 1 0 - 1 1 1 0 1 0 0 0 ? 0 - - - 0 0 1 1 0 0 - - 0 ? 0 1 3 1 1 ? 3 2 0 1 ? 0 ? 0 ? 0 ? ? 1 ? 1 0 0 ? ? ? ? ? ? ? ? ? ? 1 1 0 0 0 - 1 0 0 0 1 - ? ? 0 0 0 - 1 0 0 - 2 0 1 0 3 0 1 0 0 0 0 1 - - 0 0 0 0 0 ? ? ? ? 2 2 1 ? ? ? ? ? ? ? ? ? ? ? ? ? ? ? ? ? ? ? ? ? ? ? ? ? ? ? ? ? ? ? ? ? ? ? ? ? ? ? ? ? ? ? ? ? ? ? ? ? ? ? ? ? ? ? ? ? ? ? ?

Elanodactylus_prolatus ? ? ? ? ? ? ? ? ? ? ? ? ? ? ? ? ? ? ? ? ? ? ? ? ? ? ? ? ? ? ? ? ? ? ? ? ? ? ? ? ? ? ? ? ? ? ? ? ? ? ? ? ? ? ? ? ? ? ? ? ? ? ? ? ? ? ? ? ? ? ? ? ? ? ? ? ? ? ? ? ? ? ? ? ? ? ? ? ? ? ? ? ? ? ? ? ? ? ? ? ? ? ? ? ? ? ? ? ? ? ? ? ? ? ? ? ? ? ? ? ? ? 2 2 1 0 1 1 0 0 0 ? ? ? 0 0 0 0 0 2 ? 1 0 0 0 1 ? 1 1 1 ? 1 1 0 5 0 - 0 0 0 ? 0 4 0 0 1 1 0 0 0 ? 0 1 3 0 1 ? 0 1 1 ? 0 1 3 -

Kepodactylus_insperatus ? ? ? ? ? ? ? ? ? ? ? ? ? ? ? ? ? ? ? ? ? ? ? ? ? ? ? ? ? ? ? ? ? ? ? ? ? ? ? ? ? ? ? ? ? ? ? ? ? ? ? ? ? ? ? ? ? ? ? ? ? ? ? ? ? ? ? ? ? ? ? ? ? ? ? ? ? ? ? ? ? ? ? ? ? ? ? ? ? ? ? ? ? ? ? ? ? ? ? ? ? ? ? ? ? ? ? ? ? ? ? ? ? ? ? ? ? ? ? 0 1 0 1 2 1 0 1 ? ? ? ? ? ? ? ? ? ? ? ? ? ? ? ? ? ? ? 0 1 1 1 ? 0 1 0 5 0 - ? ? 0 ? ? ? ? ? ? 1 0 0 ? ? 0 ? ? ? ? 1 ? ? ? ? ? ? ? ?

Ctenochasma_elegans 2 1 - 0 1 1 0 - 0 1 1 - - 1 0 0 0 4 1 0 - 1 0 - - - - - - - 0 - - - 0 0 1 2 0 0 - - 1 1 0 0 - 1 1 1 3 2 0 1 0 0 0 0 1 0 - 1 1 ? ? 0 0 ? 1 ? ? ? 0 ? ? ? 1 1 0 0 0 0 - 1 0 0 0 1 - 1 0 1 2 0 - 1 0 0 - 2 0 1 1 2 0 1 0 1 2 0 1 - - 0 0 0 0 0 ? 0 ? ? 1 0 1 0 0 1 0 0 0 ? 0 0 0 0 0 0 0 2 ? ? ? 0 0 2 0 0 1 1 1 ? 1 0 5 0 - 0 0 0 ? ? 4 ? ? 1 1 0 0 ? 0 ? 1 3 0 1 1 0 1 1 0 0 1 3 -

Ctenochasma_porocristata 2 1 - 0 1 1 0 - 0 1 ? - - 1 ? ? ? ? ? 0 - 1 1 1 0 3 ? 0 0 0 0 ? ? ? ? ? ? ? ? ? ? ? ? ? 0 ? ? ? ? 1 ? ? ? ? 0 ? ? ? ? 0 - 1 ? ? ? ? ? ? ? 0 - - 0 ? ? ? ? 1 ? ? ? ? ? ? ? ? 0 ? ? ? ? ? ? ? ? ? 0 ? ? 2 0 1 1 2 ? 1 0 1 2 0 1 - - 0 0 ? 0 0 ? ? ? ? ? ? ? ? ? ? ? ? ? ? ? ? ? ? ? ? ? ? ? ? ? ? ? ? ? ? ? ? ? ? ? ? ? ? ? ? ? ? ? ? ? ? ? ? ? ? ? ? ? ? ? ? ? ? ? ? ? ? ? ? ? ? ?

Pterodaustro_guinazui 2 1 - 0 0 0 0 - 0 1 1 - - 1 0 0 0 4 1 0 - 1 0 - - - - - - - 0 - - - 0 0 1 2 0 0 - - 1 1 0 0 - 1 1 1 3 1 0 1 ? 0 0 0 0 0 - 1 1 ? ? 0 0 ? 1 0 - - 0 ? ? ? 1 0 0 0 0 0 - 1 0 0 0 1 - 1 0 0 2 0 - 1 0 0 - 2 0 1 0 0 0 2 0 1 0 0 1 - - 0 0 0 0 0 ? 0 1 0 1 0 1 0 ? 1 0 ? ? 0 0 0 0 0 0 0 0 2 1 1 1 0 0 2 0 0 ? 1 1 ? 1 0 5 ? ? 0 0 0 0 0 4 ? ? ? 1 0 0 0 0 0 ? 3 ? 1 1 0 1 1 1 ? 1 3 -

Eosipterus_yangi ? ? ? ? ? ? ? ? ? ? ? ? ? ? ? ? ? ? ? ? ? ? ? ? ? ? ? ? ? ? ? ? ? ? ? ? ? ? ? ? ? ? ? ? ? ? ? ? ? ? ? ? ? ? ? ? ? ? ? ? ? ? ? ? ? ? ? ? ? ? ? ? ? ? ? ? ? ? ? ? ? ? ? ? ? ? ? ? ? ? ? ? ? ? ? ? ? ? ? ? ? ? ? ? ? ? ? ? ? ? ? ? ? ? ? ? ? ? ? ? ? ? ? ? ? 0 ? ? ? ? ? ? ? ? ? 0 ? ? ? ? ? ? ? ? ? ? ? ? ? ? ? ? 1 ? ? ? ? ? 0 0 0 ? ? ? 0 ? 1 0 ? 0 ? 0 ? 3 ? ? 1 ? 1 ? 0 ? 1 3 -

Beipiaopterus_chenianus ? ? ? ? ? ? ? ? ? ? ? ? ? ? ? ? ? ? ? ? ? ? ? ? ? ? ? ? ? ? ? ? ? ? ? ? ? ? ? ? ? ? ? ? ? ? ? ? ? ? ? ? ? ? ? ? ? ? ? ? ? ? ? ? ? ? ? ? ? ? ? ? ? ? ? ? ? ? ? ? ? ? ? ? ? ? ? ? ? ? ? ? ? ? ? ? ? ? ? ? ? ? ? ? ? ? ? ? ? ? ? ? ? ? ? ? ? ? ? ? ? ? 1 0 1 0 ? 1 0 0 ? 1 0 0 ? 0 0 0 2 2 ? ? ? 0 0 ? 0 ? 1 1 ? ? 1 0 5 ? ? ? 0 ? ? ? 4 ? ? ? 1 0 0 0 0 ? ? ? ? ? 1 ? ? ? ? ? 1 3 -

Gegepterus_changae 2 ? ? 0 0 0 ? ? 0 1 1 - - 1 0 0 0 ? 1 0 - 1 1 ? ? 3 0 0 0 0 0 - - - 0 0 1 2 0 0 - - 1 1 0 0 - 1 ? 1 3 1 0 1 0 0 0 0 0 0 - 1 1 ? ? ? 0 ? 1 0 - - 0 ? ? ? 1 0 ? ? ? ? ? 0 0 ? 0 1 - ? ? 0 2 0 - 1 0 0 - 2 0 1 0 3 ? 1 0 1 2 0 1 - - ? 0 ? ? 0 0 0 1 ? 1 0 1 0 0 ? ? ? ? ? ? ? ? ? ? ? 2 2 ? ? ? 0 0 2 ? ? ? ? ? ? ? ? ? ? ? ? ? ? ? ? ? ? [01] ? ? ? 0 ? 0 0 ? 3 ? ? 1 ? ? ? 0 ? ? ? ?

Boreopterus_cuiae 1 1 - 0 ? 1 0 - 0 1 ? - - 1 1 1 0 ? 1 0 - 1 0 - - - - - - - 0 - - - ? ? ? ? ? 1 0 2 1 1 ? 1 0 0 0 1 2 2 0 1 ? 0 0 0 1 0 - 1 0 ? ? ? ? ? ? ? ? ? ? ? ? ? 1 1 1 0 0 0 - 1 0 0 0 1 - ? ? 1 0 0 - 1 0 0 - 3 0 1 0 3 0 1 0 0 1 0 1 - - 0 0 0 0 1 ? ? ? ? 0 1 1 0 1 ? ? ? ? 1 0 ? ? ? ? ? ? ? ? ? ? ? ? ? ? 1 0 ? ? ? ? ? ? ? ? ? ? ? ? ? ? ? 1 1 ? ? ? 1 ? 0 ? ? ? ? 1 ? 1 1 ? 1 1 3 -

Feilongus_youngi 1 1 - 0 0 1 0 - 1 1 1 - - 1 0 ? 0 4 1 0 - 1 1 1 0 1 0 0 0 0 0 - - - 0 0 1 0 0 0 - - 0 1 0 1 0 1 1 1 3 ? 0 1 0 0 0 0 1 0 - 1 1 ? 1 0 0 ? 1 0 - - 0 ? ? 0 1 1 1 0 0 0 - 1 0 0 0 1 - ? ? 0 0 0 - 1 0 0 - 2 0 1 0 3 0 1 0 0 0 0 1 - - 0 0 0 0 0 ? ? ? ? ? ? ? ? ? ? ? ? ? ? ? ? ? ? ? ? ? ? ? ? ? ? ? ? ? ? ? ? ? ? ? ? ? ? ? ? ? ? ? ? ? ? ? ? ? ? ? ? ? ? ? ? ? ? ? ? ? ? ? ? ? ? ?

Gallodactylus_canjuersensis 2 1 - 0 0 0 0 - 0 1 1 - - 1 0 0 0 ? 1 0 - 1 0 - - - - - - - 0 - - - 0 0 0 0 0 0 - - 1 ? 0 1 1 ? 1 1 ? 2 0 1 0 0 0 ? 1 0 - 1 ? ? ? ? ? ? 1 ? ? ? ? ? ? ? 1 1 2 0 0 0 - ? 0 0 0 1 - ? ? 1 1 0 - 1 ? ? ? ? ? ? ? ? ? ? ? ? ? 0 ? ? ? ? ? ? ? ? ? ? ? ? ? ? ? ? ? ? ? ? ? ? ? ? ? 0 0 ? 0 2 ? ? ? 0 0 ? ? ? ? ? ? ? ? ? ? ? ? ? ? ? ? ? 4 ? ? 1 ? 0 ? 0 ? ? 1 3 0 ? 1 ? 1 1 0 0 1 ? ?

Cycnorhamphus_suevicus 2 1 - 2 0 0 0 - 0 1 1 - - 1 0 0 0 4 1 0 - 1 0 - - - - - - - 0 - - - 0 0 0 ? 0 0 - - 1 ? 0 1 1 1 1 1 3 2 0 1 0 0 0 ? 1 0 - 1 1 ? ? ? ? ? 1 0 - - ? ? ? ? 1 1 0 2 0 0 - 1 0 0 0 1 - ? ? 1 1 0 - 1 0 0 - 2 0 1 0 ? 0 1 0 0 ? 0 1 - - 0 ? ? ? 0 0 0 0 0 0 0 0 0 1 ? 0 ? ? ? 0 0 0 0 0 ? 0 2 ? 1 1 0 0 2 0 0 1 1 ? ? 1 0 5 0 - 0 0 0 ? ? 4 ? 0 1 1 0 0 0 0 0 1 3 0 1 1 0 1 1 0 0 1 ? ?

Ardeadactylus_longicollum 2 1 - 0 0 1 0 - 0 1 1 - - 1 0 0 0 4 1 0 - 1 0 - - - - - - - 0 - - - 0 0 1 ? 0 0 - - 1 1 0 0 - 1 1 1 3 2 0 1 0 0 0 0 1 0 - 1 1 ? ? 0 0 ? 1 0 - - 0 ? ? ? 1 1 0 0 0 0 - 1 ? 0 0 1 - 1 0 1 3 0 - ? 0 0 - 3 0 0 0 3 0 1 0 0 1 0 1 - - 0 0 0 0 0 0 0 0 0 1 0 0 0 1 ? 0 0 0 ? 0 0 0 0 0 0 0 2 ? ? 0 0 0 1 1 0 0 1 1 ? 1 0 5 0 - 0 0 0 ? ? 4 ? 0 1 1 0 0 ? 0 ? 1 3 0 1 1 0 1 1 0 0 1 ? ?

Pterodactylus_kochi 1 1 - 0 0 1 0 - 0 1 1 - - 1 0 0 0 4 1 0 - 1 0 - - - - - - - 0 - - - 0 0 1 1 0 0 - - 1 1 0 0 - 1 1 1 3 2 0 1 0 0 0 0 1 0 - 1 1 ? ? ? 0 ? 1 0 - - 0 ? ? ? 1 1 0 0 0 0 - 1 0 0 0 1 - ? ? 1 3 0 - 1 0 0 - 3 0 0 0 3 0 1 0 0 0 0 1 - - 0 0 0 0 0 0 0 0 ? 1 0 0 0 1 1 0 0 0 1 0 0 0 0 0 0 0 2 ? ? 0 0 0 2 0 0 1 1 0 0 1 0 5 0 - 0 0 0 0 0 4 ? 0 1 1 0 0 1 0 0 1 3 0 1 1 0 1 1 0 0 1 3 -

Pterodactylus_antiquus 1 1 - 0 0 1 0 - 0 1 1 - - 1 0 0 0 4 1 0 - 1 0 - - - - - - - 0 - - - 0 0 1 1 0 0 - - 1 1 0 0 - 1 1 1 3 2 0 1 0 0 0 0 1 0 - 1 1 ? ? ? 0 ? 1 0 - - ? ? ? ? 1 1 0 0 0 0 - 1 0 0 0 1 - ? ? 1 3 0 - 1 0 0 - 3 0 0 0 3 0 1 0 0 0 0 1 - - 0 0 0 0 0 0 0 0 ? 1 0 0 0 1 1 0 0 0 1 0 0 0 0 0 0 0 2 0 1 0 0 0 2 0 0 1 1 ? ? 1 0 5 0 - 0 0 0 ? 0 4 0 0 1 1 0 0 1 0 0 1 3 0 1 1 0 1 1 0 0 1 3 -

Normannognathus_wellnhoferi 1 1 - 0 0 0 0 - ? 1 ? ? ? ? ? ? ? ? ? ? ? ? 1 1 2 ? ? 0 0 0 ? ? ? ? ? ? ? ? ? ? ? ? ? ? ? ? ? ? ? ? ? ? ? ? ? ? ? ? ? ? ? ? ? ? ? ? ? ? 1 0 - - ? ? ? ? ? ? 0 0 0 0 - 1 ? ? 0 ? ? 1 0 ? ? ? ? ? 0 0 - 3 0 0 0 3 0 1 0 0 ? 0 1 - - 0 1 0 0 0 ? ? ? ? ? ? ? ? ? ? ? ? ? ? ? ? ? ? ? ? ? ? ? ? ? ? ? ? ? ? ? ? ? ? ? ? ? ? ? ? ? ? ? ? ? ? ? ? ? ? ? ? ? ? ? ? ? ? ? ? ? ? ? ? ? ? ?

Germanodactylus_cristatus 1 3 0 0 0 1 0 - 0 1 1 - - 1 0 0 0 4 1 0 - 1 1 1 0 3 2 0 0 0 0 - - - 0 0 1 0 0 0 - - 1 1 0 0 - 1 0 1 3 2 0 1 0 0 0 0 1 0 - 1 1 ? ? ? 0 ? 1 ? ? ? ? ? ? ? 1 1 2 0 0 0 - 1 0 0 0 1 - ? ? 1 3 0 - 1 0 0 - 3 0 0 0 3 0 1 0 0 0 0 1 - - 1 1 0 1 0 0 ? ? ? ? ? 0 0 1 1 0 0 0 ? ? 0 0 0 0 0 0 2 ? ? ? 0 0 2 0 0 1 1 0 ? 1 0 5 ? ? 0 0 0 ? ? 4 ? ? 1 1 0 0 1 0 ? 1 3 0 1 1 0 1 1 0 0 1 ? ?

Germanodactylus_rhamphastinus 1 3 0 0 0 1 0 - 0 1 1 - - 1 0 0 0 4 1 0 - 1 1 1 0 3 2 0 0 0 0 - - - 0 0 1 0 0 0 - - 1 1 0 0 - 1 0 1 3 2 0 1 0 0 0 0 1 0 - 1 1 ? ? ? 0 ? 1 ? ? ? ? ? ? ? 1 1 0 0 0 0 - ? 0 0 0 1 - ? ? 1 3 0 - 1 0 0 - 3 0 0 0 3 0 1 0 0 0 0 1 - - 0 1 0 0 0 0 ? ? ? 1 0 0 0 1 ? ? 0 ? ? ? ? 0 0 0 0 ? 2 0 ? ? 0 0 2 ? ? 1 1 ? ? 1 ? ? ? ? 0 ? 0 0 ? 4 ? ? 1 ? 0 0 1 0 ? 1 3 0 1 1 0 1 1 0 0 1 ? ?

Haopterus_gracilis 1 1 - 0 0 1 0 - 0 1 ? - - 1 ? 0 0 ? ? 0 - ? 0 - - - - - - - 0 - - - 0 1 0 0 0 0 - - ? 0 0 0 - 0 ? 1 2 3 0 ? ? ? ? ? ? 0 - 1 ? ? ? ? 0 ? 0 ? ? ? ? ? ? ? 1 1 1 0 0 0 - 1 ? 0 0 1 - ? ? 1 0 0 - ? 0 0 - 3 0 0 0 3 0 1 0 0 0 0 1 - - 0 0 0 0 0 ? ? ? ? ? ? 0 0 1 ? 0 0 ? ? ? ? 1 0 0 0 0 2 0 1 1 ? ? 2 ? 0 0 1 ? ? 1 0 7 0 - ? ? ? ? ? 4 ? 0 1 ? 0 0 1 0 0 ? ? ? ? ? ? ? ? ? ? 1 3 -

Anhanguera_santanae 1 1 - 0 0 1 1 0 0 1 0 - - 1 1 1 0 2 1 0 - 1 1 1 0 4 0 ? 1 1 0 - - - 0 1 1 0 1 1 0 2 1 1 1 1 0 0 0 1 2 3 0 1 1 0 1 0 1 0 - 1 0 1 1 0 0 1 0 1 0 0 0 1 1 1 1 1 0 0 ? 0 - 1 0 0 0 1 - 1 0 1 0 ? ? 1 0 1 2 5 1 ? 0 3 1 1 1 1 1 0 1 - - 0 0 1 0 1 1 0 1 1 0 1 1 0 1 0 1 0 0 1 0 ? 1 1 1 0 0 2 ? ? ? 1 1 ? 0 1 0 0 ? ? 2 0 7 1 0 0 0 0 1 1 4 0 1 ? 1 ? ? ? ? 0 1 2 1 ? ? 0 1 1 ? ? ? ? ?

Anhanguera_piscator 1 1 - 0 0 1 1 0 0 1 0 - - 1 1 1 0 2 1 0 - 1 1 1 0 4 0 0 1 1 0 - - - 0 1 1 0 1 1 0 2 1 1 1 1 0 0 0 1 2 3 0 1 1 0 1 0 1 0 - 1 0 1 1 0 0 1 0 ? ? ? ? 1 ? 1 1 1 0 0 1 0 - 1 0 0 0 1 - ? ? 1 0 1 0 1 0 1 2 5 1 1 0 3 1 1 1 1 1 0 1 - - 0 0 1 0 1 1 0 1 1 0 1 1 0 1 0 1 0 0 1 0 1 ? 1 1 0 0 2 0 0 1 1 1 2 0 1 0 0 0 1 2 0 7 1 0 0 ? 0 1 1 4 ? 1 ? 1 0 1 1 1 0 1 2 1 ? 1 0 1 1 0 1 ? 3 -

Anhanguera_blittersdorffi 1 1 - 0 0 1 1 0 0 1 0 - - 1 1 1 0 2 1 0 - 1 1 1 0 4 0 0 1 1 0 - - - 1 - - - - 1 0 2 1 1 1 1 0 0 0 1 2 3 0 1 1 0 1 0 1 0 - 1 0 1 1 0 0 1 0 1 0 0 0 1 1 1 1 1 0 0 1 0 - 1 0 0 0 1 - 1 0 1 ? 1 0 ? 0 1 2 5 1 1 0 3 1 1 1 1 1 0 1 - - 0 0 1 0 1 ? ? ? ? ? ? ? ? ? ? ? ? ? ? ? ? ? ? ? ? ? ? ? ? ? ? ? ? ? ? ? ? ? ? ? ? ? ? ? ? ? ? ? ? ? ? ? ? ? ? ? ? ? ? ? ? ? ? ? ? ? ? ? ? ? ? ?

Anhanguera_araripensis 1 1 - 0 0 1 1 0 0 1 0 - - 1 1 1 0 2 1 0 - 1 1 1 0 4 0 0 1 1 0 - - - 1 - - - - 1 0 2 1 1 1 1 0 0 0 1 2 3 0 1 1 0 1 0 1 0 - 1 0 1 1 0 0 1 0 1 0 0 0 1 1 1 1 ? ? 0 ? ? ? 1 0 ? 0 1 - ? ? 1 0 ? ? 1 0 1 2 5 ? ? 0 3 1 1 1 1 1 0 1 - - 0 0 1 ? 1 ? ? ? ? ? ? ? ? ? ? ? ? ? ? ? ? ? ? ? ? ? ? ? ? ? ? ? ? 0 1 0 0 0 1 2 0 7 1 0 ? 0 0 1 1 4 ? 1 ? 1 ? ? ? ? ? ? ? ? ? ? ? ? ? ? ? ? ? ?

Liaoningopterus_gui 1 1 - 0 0 1 1 0 0 1 0 - - 1 1 1 [02] 2 1 0 - ? 1 1 0 4 0 0 1 1 0 - - - ? ? ? ? ? ? ? ? ? ? 1 ? ? 0 0 1 2 3 0 1 ? 0 1 0 1 0 - 1 ? ? ? ? ? ? ? 1 0 0 ? ? ? 1 1 1 0 0 ? 0 - ? 0 0 0 1 - ? ? 1 0 ? ? ? 0 1 2 5 1 1 0 3 1 1 0 1 1 0 1 - - 0 0 1 0 1 ? 0 1 ? ? ? 1 0 1 ? ? ? ? ? ? ? ? ? ? ? ? ? ? ? ? ? ? ? ? ? ? ? ? ? ? ? ? ? ? ? ? ? ? ? ? ? ? ? ? ? ? ? ? ? ? ? ? ? ? ? ? ? ? ? ? ? ?

Tropeognathus_mesembrinus 1 1 - 0 0 1 1 0 0 1 0 - - 1 1 1 0 2 1 0 - ? 1 0 0 4 0 0 1 1 0 - - - 1 - - - - 1 0 2 1 1 1 1 0 0 0 1 2 3 0 1 1 0 1 0 1 0 - 1 0 1 1 0 0 1 0 1 0 1 0 1 1 1 1 1 0 0 1 0 - 1 0 0 0 1 - 1 0 1 3 1 0 ? 0 1 2 5 1 ? 0 3 1 1 0 1 1 0 1 - - 0 0 0 0 1 ? ? ? ? ? ? ? ? ? ? ? ? ? ? ? ? ? ? ? ? ? ? ? ? ? ? ? ? ? ? ? ? ? ? ? ? ? ? ? ? ? ? ? ? ? ? ? ? ? ? ? ? ? ? ? ? ? ? ? ? ? ? ? ? ? ? ?

Coloborhynchus_clavirostris ? 0 - 0 1 1 1 2 ? ? ? ? ? ? ? ? ? ? ? ? ? ? 1 0 0 4 ? 0 1 1 ? ? ? ? ? ? ? ? ? ? ? ? ? ? ? ? ? ? ? ? ? ? ? ? ? ? ? ? ? ? ? ? ? ? ? ? ? ? ? 1 0 1 ? ? ? ? 1 ? ? ? ? ? ? ? ? ? ? ? ? ? ? ? ? ? ? ? 0 ? 2 5 ? ? 0 3 1 ? ? ? 1 0 ? ? ? 0 ? 1 ? 1 ? ? ? ? ? ? ? ? ? ? ? ? ? ? ? ? ? ? ? ? ? ? ? ? ? ? ? ? ? ? ? ? ? ? ? ? ? ? ? ? ? ? ? ? ? ? ? ? ? ? ? ? ? ? ? ? ? ? ? ? ? ? ? ? ? ? ?

Ornithocheirus_simus ? 0 - 0 0 1 1 0 ? ? ? ? ? ? ? ? ? ? ? ? ? ? 1 0 0 4 ? 0 1 1 ? ? ? ? ? ? ? ? ? ? ? ? ? ? ? ? ? ? ? ? ? ? ? ? ? ? ? ? ? ? ? ? ? ? ? ? ? ? ? 1 0 1 ? ? ? ? 1 ? ? ? ? ? ? ? ? ? ? ? ? ? ? ? ? ? ? ? 0 ? 2 5 ? ? 0 ? ? ? ? ? 0 0 ? ? ? 0 ? ? ? 1 ? ? ? ? ? ? ? ? ? ? ? ? ? ? ? ? ? ? ? ? ? ? ? ? ? ? ? ? ? ? ? ? ? ? ? ? ? ? ? ? ? ? ? ? ? ? ? ? ? ? ? ? ? ? ? ? ? ? ? ? ? ? ? ? ? ? ?

Brasileodactylus_araripensis ? ? ? ? 0 ? ? 0 ? ? ? ? ? ? ? ? ? ? ? ? ? ? ? ? ? ? ? ? ? ? ? ? ? ? ? ? ? ? ? ? ? ? ? ? ? ? ? ? ? ? ? ? ? ? ? ? ? ? ? ? ? ? ? ? ? ? ? ? ? ? ? ? ? ? ? ? 1 1 0 0 1 0 - 1 0 0 0 1 - 1 0 ? ? 0 - ? 0 1 2 5 0 ? 0 3 ? ? ? 1 1 0 1 - - 0 ? 1 0 1 ? ? ? ? ? ? ? ? ? ? ? ? ? ? ? ? ? ? ? ? ? ? ? ? ? ? ? ? ? ? ? ? ? ? ? ? ? ? ? ? ? ? ? ? ? ? ? ? ? ? ? ? ? ? ? ? ? ? ? ? ? ? ? ? ? ? ?

Ludodactylus_sibbicki 1 1 - 0 ? 1 1 0 0 1 0 - - 1 1 1 0 2 1 0 - 1 0 - - - - - - - 0 - - - 0 1 1 0 1 1 1 1 1 1 1 1 2 0 0 1 2 2 0 1 1 0 1 0 0 0 - 1 0 1 1 0 0 ? 0 1 0 1 0 1 ? 1 1 1 0 0 1 0 - 1 0 0 0 1 - 1 0 1 0 0 0 1 0 1 2 5 1 1 0 3 1 1 0 1 1 0 1 - - 0 0 1 0 1 ? ? ? ? ? ? ? ? ? ? ? ? ? ? ? ? ? ? ? ? ? ? ? ? ? ? ? ? ? ? ? ? ? ? ? ? ? ? ? ? ? ? ? ? ? ? ? ? ? ? ? ? ? ? ? ? ? ? ? ? ? ? ? ? ? ? ?

Guidraco_venator 1 1 - 0 ? 1 ? ? 0 1 0 - - 1 1 1 0 2 1 0 - 1 0 - - - - - - - 0 - - - ? ? ? ? ? 1 2 1 1 1 0 1 2 0 0 1 2 2 0 1 0 0 0 0 0 0 - 1 0 ? ? ? 0 ? ? ? ? ? 0 ? ? 1 1 1 1 0 ? 0 - 1 0 0 0 1 - ? ? 1 0 0 - 1 0 1 2 5 0 1 0 3 1 1 0 1 1 0 1 - - 0 0 0 0 1 ? 0 1 ? ? ? 1 0 1 ? ? ? ? ? ? ? ? ? ? ? ? ? ? ? ? ? ? ? ? ? ? ? ? ? ? ? ? ? ? ? ? ? ? ? ? ? ? ? ? ? ? ? ? ? ? ? ? ? ? ? ? ? ? ? ? ? ?

Zhenyuanopterus_longirostris 1 1 - 0 ? 1 0 - 0 1 1 - - 1 1 1 0 4 1 0 - 1 1 1 2 3 1 0 ? ? 0 - - - 1 1 1 0 0 1 0 2 1 1 0 1 0 0 0 1 2 2 0 1 0 0 0 0 1 0 - 1 0 ? ? ? 0 ? ? ? ? ? ? ? ? ? 1 1 ? 0 0 0 - 1 0 0 0 1 - ? ? 1 0 0 - 1 0 0 - 3 0 1 0 3 0 1 0 0 1 0 1 - - 0 0 0 0 1 ? ? 1 ? 0 1 1 0 1 ? 1 1 1 1 0 ? 1 1 1 1 0 2 ? ? ? ? ? 2 ? ? 0 0 ? ? 2 0 7 1 0 ? ? ? ? ? 4 ? ? 1 1 0 ? 1 ? ? ? ? ? ? 1 ? 1 1 ? 1 1 3 -

Cearadactylus_atrox 1 1 - 0 0 1 1 0 0 1 ? - - 1 ? ? 0 2 1 0 - ? 0 - - - - - - - 0 - - - ? ? ? ? ? ? ? ? ? ? ? ? ? ? 0 ? 3 3 0 1 ? 0 1 0 1 ? ? 1 ? ? ? ? ? ? ? ? ? ? ? ? ? ? 1 1 0 0 1 1 0 1 0 0 0 1 - ? ? 1 0 0 - 1 0 1 2 5 0 1 0 3 1 1 0 1 1 0 1 - - 0 0 0 0 1 ? ? ? ? ? ? ? ? ? ? ? ? ? ? ? ? ? ? ? ? ? ? ? ? ? ? ? ? ? ? ? ? ? ? ? ? ? ? ? ? ? ? ? ? ? ? ? ? ? ? ? ? ? ? ? ? ? ? ? ? ? ? ? ? ? ? ?

Hongshanopterus_lacustris ? 2 - 0 0 1 0 - 0 ? ? - - 1 1 ? 0 ? 1 0 - ? ? ? ? ? ? ? ? ? 0 - - ? 0 1 0 0 0 ? ? ? ? ? ? ? ? 0 ? 1 ? [23] 0 ? ? ? 0 0 1 ? - 1 0 1 1 0 ? ? 0 1 0 0 0 1 1 1 1 ? ? ? ? ? ? ? ? ? ? ? ? ? ? ? ? ? ? ? 0 0 - 0 0 0 0 3 0 ? 0 0 0 0 1 - - 0 0 0 - 0 1 0 1 ? 0 1 1 0 1 ? ? ? ? ? ? ? ? ? ? ? ? ? ? ? ? ? ? ? ? ? ? ? ? ? ? ? ? ? ? ? ? ? ? ? ? ? ? ? ? ? ? ? ? ? ? ? ? ? ? ? ? ? ? ? ? ? ?

Lonchodectes_compressirostris 1 ? ? 0 0 1 0 - 0 ? ? ? ? ? ? ? ? ? ? ? ? ? 0 - - - - - - - ? ? ? ? ? ? ? ? ? ? ? ? ? ? ? ? ? ? ? ? ? ? 0 ? ? ? ? ? ? ? ? ? ? ? ? ? ? ? 0 1 0 1 ? ? ? ? 1 1 ? ? ? ? ? ? ? ? ? ? ? ? 0 ? ? 0 - ? 0 0 - 5 ? ? 0 3 0 ? ? ? 0 0 ? ? ? 0 0 0 0 0 ? ? ? ? ? ? ? ? ? ? ? ? ? ? ? ? ? ? ? ? ? ? ? ? ? ? ? ? ? ? ? ? ? ? ? ? ? ? ? ? ? ? ? ? ? ? ? ? ? ? ? ? ? ? ? ? ? ? ? ? ? ? ? ? ? ? ?

Nurhachius_ignaciobritoi 1 1 - 0 0 1 0 - 0 1 0 - - 1 0 1 0 ? ? 0 - 1 0 - - - - - - - 0 - - - 0 1 0 0 0 0 - - 1 1 1 ? ? 0 ? 1 3 3 0 ? ? 0 0 1 1 ? - 1 ? ? ? ? ? ? ? 1 1 1 0 ? ? ? 1 1 1 0 0 0 - 1 0 0 0 1 - ? ? 1 0 0 - 1 0 0 - 4 0 0 0 3 0 1 0 1 0 0 1 - - 0 0 0 0 1 ? 0 1 ? 0 1 1 0 1 ? ? 1 ? ? ? ? ? ? 1 ? 0 2 1 ? ? 1 0 2 0 ? 0 0 0 ? ? 0 7 1 0 1 0 ? 1 ? 4 ? 1 ? 1 0 0 1 1 ? ? 3 ? ? 1 ? ? ? 0 ? ? ? ?

Liaoxipterus_brachyognathus ? ? ? ? 1 ? ? - ? ? ? ? ? ? ? ? ? ? ? ? ? ? ? ? ? ? ? ? ? ? ? ? ? ? ? ? ? ? ? ? ? ? ? ? ? ? ? ? ? ? ? ? ? ? ? ? ? ? ? ? ? ? ? ? ? ? ? ? ? ? ? ? ? ? ? ? ? 1 ? 2 0 0 - 1 0 ? 0 1 - ? ? 1 2 ? ? ? 0 0 - 4 0 0 0 1 0 ? 0 1 0 0 1 - - ? ? 0 0 ? ? ? ? ? ? ? ? ? ? ? ? ? ? ? ? ? ? ? ? ? ? ? ? ? ? ? ? ? ? ? ? ? ? ? ? ? ? ? ? ? ? ? ? ? ? ? ? ? ? ? ? ? ? ? ? ? ? ? ? ? ? ? ? ? ? ? ?

Istiodactylus_sinensis 1 1 - 2 1 1 0 - 0 1 0 - - 1 0 0 1 2 1 0 - 1 0 - - - - - - - 0 - - - 1 - - - - 0 - - 1 ? 1 0 - 0 0 1 3 4 0 1 1 0 0 1 2 1 - 1 0 ? ? ? 0 ? ? 1 1 1 0 ? ? 0 1 1 1 ? 0 0 - 1 0 0 0 1 - ? ? 1 2 0 - 1 0 0 - 4 3 0 0 1 0 1 0 1 0 0 1 - - 0 0 0 0 1 1 ? ? ? 0 1 ? 0 1 ? ? 1 ? ? ? ? ? ? 1 ? 0 2 ? ? ? 0 0 ? ? 0 0 0 ? ? ? 0 7 ? ? ? 0 0 ? ? ? 0 1 ? ? 0 0 ? ? 0 1 3 1 ? 1 ? 1 ? 0 ? ? ? ?

Istiodactylus_latidens 1 1 - 2 1 1 0 - 0 1 0 - - 1 0 0 1 2 1 0 - ? 0 - - - - - - - 0 - - - 1 - - - - 0 - - 1 1 1 0 ? 0 0 1 3 4 0 1 ? 0 0 1 2 1 - 1 0 ? ? ? ? 1 0 ? ? ? ? ? ? 0 1 1 1 2 0 0 - 1 0 0 0 1 - ? ? 1 2 0 - 1 0 0 - 4 3 0 0 1 0 1 0 1 0 0 1 - - 0 0 0 0 1 ? ? ? ? 0 ? 1 0 ? ? 1 1 1 ? ? ? 1 1 1 1 0 2 1 0 1 1 0 ? 0 ? 0 0 0 1 2 0 7 1 0 1 0 0 1 1 4 ? ? ? 1 0 0 ? 1 ? 1 3 1 ? 1 1 1 1 0 1 ? ? ?

Longchengopterus_zhaoi 1 1 - 0 0 1 0 - 0 1 ? - - 1 0 1 0 ? 1 0 ? 1 0 - - - - - - - 0 - - - 0 1 0 ? 0 0 - - ? ? 1 0 - 0 0 ? 3 3 0 1 ? 0 0 1 1 0 ? ? 0 ? ? ? 0 ? ? 1 1 1 0 ? ? 0 ? 1 1 0 0 0 - 1 0 ? 0 1 - 1 0 1 2 ? ? ? 0 0 - 4 0 0 0 3 0 1 0 1 0 0 1 - - 0 0 0 0 1 1 ? 1 ? 0 1 1 0 1 ? ? ? ? ? ? ? ? 1 1 ? 0 2 ? ? ? 1 0 ? 0 ? 0 0 ? ? ? ? ? 1 0 ? 0 ? ? ? 4 ? [12] ? ? ? 0 ? 1 ? ? ? ? ? 1 ? 1 ? ? 1 ? ? ?

Pteranodon_longiceps 2 3 0 0 0 0 0 - 0 - 0 - - 1 1 1 0 4 1 0 - 1 0 - - - - - - - 0 - - - 0 1 0 ? 0 1 2 0 0 1 0 1 2 0 0 1 2 4 0 1 1 0 1 0 1 0 - 1 0 1 1 0 0 1 0 0 - - 0 1 1 1 1 1 2 0 0 0 - 1 1 0 0 1 - 0 0 1 0 0 - 1 1 - - - - - - - - - - - - - - - - - - - - 0 1 0 1 1 0 1 1 0 1 0 1 1 1 1 0 1 1 1 0 1 0 2 0 0 1 1 0 1 1 0 0 0 1 0 2 0 7 1 0 0 0 0 1 1 4 0 2 1 1 0 0 1 1 0 1 1 0 1 1 1 1 1 0 1 1 3 -

Pteranodon_sternbergi 2 3 0 0 0 0 0 - 0 - 0 - - 1 1 1 0 4 1 0 - 1 0 - - - - - - - 0 - - - 0 1 0 0 ? 1 2 0 0 1 0 1 2 0 0 1 2 3 0 1 1 0 1 0 1 0 - 1 0 ? ? 0 0 ? ? ? ? ? ? ? ? ? 1 1 2 0 0 0 - 1 1 0 0 1 - ? ? 1 0 ? ? ? 1 - - - - - - - - - - - - - - - - - - - - ? ? ? ? ? ? ? ? 0 ? ? ? ? ? ? ? 1 ? ? ? ? ? 2 0 ? ? 1 0 1 ? ? 0 ? 1 ? ? ? ? ? ? ? 0 ? 1 ? 2 0 ? ? 1 0 0 ? ? 0 ? ? ? ? 1 ? ? ? 0 1 ? ? ?

Nyctosaurus_gracilis 1 3 0 0 0 1 0 - 0 - 0 - - 1 1 0 0 2 1 0 - 1 0 - - - - - - - 0 - - - 1 - - - - 1 2 0 1 1 0 1 1 0 0 1 2 3 0 1 ? 0 0 0 1 0 - 1 0 1 1 0 0 ? 0 0 - - 0 1 1 1 1 1 2 0 0 0 - 1 1 0 0 1 - 0 0 1 0 0 - 1 1 - - - - - - - - - - - - - - - - - - - - 0 1 0 0 1 0 0 1 0 1 0 1 1 0 1 0 0 1 1 0 0 0 2 0 0 0 0 0 1 1 0 0 1 0 1 1 1 6 1 0 0 0 0 1 1 1 ? 2 1 1 0 0 ? 1 0 1 2 0 1 1 1 1 1 0 1 1 ? ?

Nyctosaurus_lamegoi ? ? ? ? ? ? ? ? ? ? ? ? ? ? ? ? ? ? ? ? ? ? ? ? ? ? ? ? ? ? ? ? ? ? ? ? ? ? ? ? ? ? ? ? ? ? ? ? ? ? ? ? ? ? ? ? ? ? ? ? ? ? ? ? ? ? ? ? ? ? ? ? ? ? ? ? ? ? ? ? ? ? ? ? ? ? ? ? ? ? ? ? ? ? ? ? ? ? ? ? ? ? ? ? ? ? ? ? ? ? ? ? ? ? ? ? ? ? ? ? ? ? ? ? ? ? ? ? ? ? ? ? ? ? ? ? ? ? ? ? ? ? ? ? ? ? ? ? 0 1 ? ? ? 1 6 ? ? ? ? ? ? ? ? ? ? ? ? ? ? ? ? ? ? ? ? ? ? ? ? ? ? ? ? ? ?

Muzquizopteryx_coahuilensis ? ? ? ? ? ? ? ? 0 - ? - - 1 1 0 0 2 1 0 - 1 0 - - - - - - - 0 - - - 1 - - - 0 0 - - 1 1 0 1 1 0 0 1 2 2 0 1 0 0 0 0 1 0 - 1 0 ? ? ? 0 ? ? ? ? ? ? ? ? ? 1 ? ? ? ? ? ? ? ? ? ? 1 - ? ? 0 3 ? ? 1 1 - - - - - - - - - - - - - - - - - - - - 0 1 ? ? ? 0 0 ? 0 1 ? ? 1 ? 1 0 0 1 1 0 0 ? ? ? 0 0 ? ? 1 ? ? 0 1 ? ? 1 1 6 1 0 ? ? ? ? ? 1 0 2 ? 1 ? ? ? ? ? ? ? ? 1 1 ? 1 1 ? 1 1 3 -

Tupandactylus_navigans 0 3 0 0 0 2 0 - 0 - 1 - - 1 0 1 2 4 1 0 - 1 1 1 1 0 1 1 1 0 0 - - - 0 0 1 1 0 ? ? ? 0 1 0 ? 0 0 0 1 2 3 0 1 0 0 0 1 1 0 - 1 0 ? ? 0 ? ? 0 ? ? ? ? ? ? ? ? ? ? ? ? ? ? ? ? ? ? ? ? ? ? ? ? ? ? ? 1 - - - - - - - - - - - - - - - - - - - - 0 ? ? ? ? ? ? ? ? ? ? ? ? ? ? ? ? ? ? ? ? ? ? ? ? ? ? ? ? ? ? ? ? ? ? ? ? ? ? ? ? ? ? ? ? ? ? ? ? ? ? ? ? ? ? ? ? ? ? ? ? ? ? ? ? ? ? ?

Tupandactylus_imperator 0 3 0 0 0 2 0 - 0 - ? - - 1 ? 1 2 3 1 0 - 1 1 1 1 0 1 1 1 0 0 - - - 1 - - - - 1 1 2 0 ? 0 1 3 0 0 1 2 3 0 1 0 0 0 1 1 0 - 1 0 ? ? ? 1 ? 0 ? ? ? ? ? ? ? 1 2 1 0 0 1 0 1 0 0 1 1 - 0 1 1 ? 1 1 ? 1 - - - - - - - - - - - - - - - - - - - - 0 ? ? ? ? ? ? ? ? ? ? ? ? ? ? ? ? ? ? ? ? ? ? ? ? ? ? ? ? ? ? ? ? ? ? ? ? ? ? ? ? ? ? ? ? ? ? ? ? ? ? ? ? ? ? ? ? ? ? ? ? ? ? ? ? ? ? ?

Bakonydraco_galaczi ? ? ? ? 0 ? ? - ? ? ? ? ? ? ? ? ? ? ? ? ? ? ? ? ? ? ? ? ? ? ? ? ? ? ? ? ? ? ? ? ? ? ? ? ? ? ? ? ? ? ? ? ? ? ? ? ? ? ? ? ? ? 0 ? ? ? ? ? ? ? ? ? ? ? ? 1 1 2 2 0 0 1 1 1 0 0 0 1 - 0 1 1 0 1 0 1 1 - - - - - - - - - - - - - - - - - - - - ? ? ? ? ? ? ? ? ? ? ? ? ? ? ? ? ? ? ? ? ? ? ? ? ? ? ? ? ? ? ? ? ? ? ? ? ? ? ? ? ? ? ? ? ? ? ? ? ? ? ? ? ? ? ? ? ? ? ? ? ? ? ? ? ? ? ? ?

Tapejara_wellnhoferi 0 3 0 0 0 2 0 - 0 - 1 - - 1 0 1 2 4 1 0 - 1 1 1 1 0 1 0 1 1 0 - - - 0 0 1 1 0 1 1 2 0 1 0 1 3 0 0 1 2 3 0 1 0 0 0 1 1 0 - 1 0 1 1 0 1 1 1 0 - - 0 1 1 0 1 2 2 0 0 1 1 1 0 0 1 1 - 0 1 1 0 1 1 1 1 - - - - - - - - - - - - - - - - - - - - 0 ? 0 1 1 0 0 1 0 1 0 1 0 0 ? ? 0 1 0 0 1 2 2 1 1 1 0 0 [12] 1 1 0 1 1 0 1 0 8 1 1 1 1 1 0 0 4 1 ? ? 1 0 0 0 1 0 1 1 1 ? 1 1 1 1 1 0 1 3 -

Europejara_olcadesorum ? ? ? 0 0 ? ? - ? ? ? - - 1 ? ? ? 4 1 ? - ? ? ? ? ? ? ? ? ? 0 - - - ? ? ? ? ? ? ? ? ? 1 ? ? ? 0 0 ? 2 ? 0 ? 0 0 ? ? ? 0 ? 1 ? ? ? ? ? ? 1 ? ? ? 0 1 ? 0 1 2 2 0 0 1 1 1 0 0 0 1 - 0 1 1 0 1 1 1 1 - - - - - - - - - - - - - - - - - - - - 0 ? ? ? ? ? ? ? ? ? ? ? ? ? ? ? ? ? ? ? ? ? ? ? ? ? ? ? ? ? ? ? ? ? ? ? ? ? ? ? ? ? ? ? ? ? ? ? ? ? ? ? ? ? ? ? ? ? ? ? ? ? ? ? ? ? ? ?

Huaxiapterus_benxiensis 0 3 0 0 0 2 0 - 0 - ? - - 1 0 ? ? ? ? 0 - 1 1 1 2 6 1 0 1 1 0 - - - 0 - 1 0 ? 1 1 2 0 ? 0 1 3 ? 0 1 3 3 0 1 0 0 0 1 1 0 - 1 0 ? ? ? ? ? ? ? ? ? ? ? ? ? 1 2 2 0 0 0 - 1 0 0 0 1 - 0 1 1 2 1 0 ? 1 - - - - - - - - - - - - - - - - - - - - 0 ? 0 1 ? ? ? 1 0 1 ? ? 0 ? ? ? ? ? ? ? ? ? ? ? ? ? ? ? ? ? ? 0 1 ? ? ? 0 8 ? ? ? ? ? ? 0 ? ? 2 1 1 0 ? 1 1 0 ? ? ? ? 1 ? ? ? ? ? ? 3 -

Huaxiapterus_corollatus 0 3 0 0 0 2 0 - 0 - ? - - 1 ? ? 2 ? ? 0 - 1 1 1 2 6 1 0 1 1 0 - - - ? ? ? ? ? ? ? ? 0 ? ? 1 0 ? ? ? ? ? ? ? 0 ? ? ? ? ? ? 1 ? ? ? ? 1 ? ? ? ? ? ? ? ? ? 1 2 2 0 0 0 - 1 0 0 0 1 - ? ? ? 2 1 0 1 1 - - - - - - - - - - - - - - - - - - - - 0 ? 0 1 ? ? ? 1 0 1 ? ? 0 ? ? ? ? ? 0 0 ? 2 2 ? ? ? 0 0 ? ? ? 0 1 ? ? ? 0 8 1 ? 1 1 1 ? ? 4 ? 2 1 1 0 ? 1 1 0 ? ? ? 1 1 1 ? 1 ? ? 1 3 -

Eopteranodon_lii 0 3 0 0 0 2 0 - 0 - ? - - 1 0 ? ? ? ? 0 - ? 1 1 0 4 1 0 1 1 0 - - - ? ? ? ? ? ? ? ? ? ? ? ? 3 ? ? 1 ? ? 0 ? 0 ? 0 1 1 ? - 1 0 ? ? ? ? ? ? ? - - ? ? ? 1 ? 2 2 0 0 0 - 1 0 ? 0 1 - 0 0 1 ? ? ? 1 1 - - - - - - - - - - - - - - - - - - - - 0 ? ? ? ? 0 0 1 0 1 ? ? ? ? ? ? ? ? ? 0 ? 2 2 ? ? ? 0 0 2 ? ? 0 1 1 ? 1 0 8 1 ? ? 1 ? ? ? 4 ? ? ? ? ? 0 ? ? 0 1 2 0 1 1 ? 1 1 1 0 ? ? ?

Sinopterus_gui 0 3 0 0 ? 2 0 - 0 - 1 - - 1 0 ? ? ? 1 0 - 1 1 1 0 1 1 0 1 1 0 - - - ? ? ? ? ? ? ? ? 0 1 ? ? 0 ? ? 1 3 3 0 1 0 ? 0 1 1 ? - 1 0 ? ? ? ? ? ? ? ? ? 0 ? ? ? 1 2 2 0 0 0 - 1 0 0 0 1 - ? ? 1 ? 1 0 1 1 - - - - - - - - - - - - - - - - - - - - 0 ? ? ? ? 0 0 1 0 1 ? ? 0 0 ? ? ? 1 ? 0 ? 2 2 ? 1 ? 0 0 ? ? ? 0 1 1 ? 1 0 8 1 1 ? 1 1 ? ? 4 ? ? ? ? ? ? ? ? ? 1 2 0 1 1 ? ? ? ? ? 1 ? ?

Huaxiapterus_jii 0 3 0 0 0 2 0 - 0 - ? - - 1 0 ? ? 4 1 0 - ? 1 1 0 4 1 0 ? 1 0 - - - 0 0 ? 1 0 ? ? ? ? ? ? ? 0 ? ? 1 3 ? 0 1 ? ? 0 1 1 ? - 1 ? ? ? ? ? ? ? 0 - - 0 ? ? ? 1 2 2 0 0 0 - 1 0 0 0 1 - 0 0 1 2 1 0 1 1 - - - - - - - - - - - - - - - - - - - - 0 ? ? ? ? ? ? ? 0 1 ? ? 0 ? 1 ? ? ? ? ? ? 2 2 ? ? ? 0 0 2 1 ? 0 1 ? ? 1 0 8 1 1 ? ? ? ? ? 4 1 1 1 1 0 ? 1 1 0 ? ? ? 1 1 ? 1 1 1 0 ? ? ?

Sinopterus_dongi 0 3 0 0 0 2 0 - 0 - 1 - - 1 0 0 2 ? 1 0 - 1 1 1 0 4 1 0 1 1 0 - - - 0 0 1 1 0 1 1 2 0 1 ? 1 3 0 ? 1 3 3 ? 1 0 ? 0 1 1 ? - 1 ? ? ? ? 1 ? ? ? ? ? ? ? ? ? 1 2 2 0 0 0 - 1 0 0 0 1 - ? ? 1 2 1 0 1 1 - - - - - - - - - - - - - - - - - - - - 0 ? ? ? ? 0 0 1 0 1 ? ? 0 ? 1 0 ? 1 ? 0 ? 2 2 ? 1 1 0 0 2 1 ? 0 1 ? ? 1 0 8 1 1 1 1 1 0 0 4 1 2 1 1 0 0 1 1 0 ? ? ? 1 1 ? ? ? 1 ? 1 3 -

Nemicolopterus_crypticus 0 ? ? 0 ? 2 0 - 0 - 1 - - 1 0 0 0 4 1 0 - 1 1 ? 0 1 1 0 ? 1 0 - - - 0 1 1 0 0 1 1 2 0 1 0 1 0 0 0 1 3 3 0 1 0 0 0 1 1 0 - 0 0 ? ? ? ? ? ? ? ? ? 0 ? ? ? 1 2 ? 0 0 0 - 1 0 0 0 1 - ? ? 1 2 1 0 ? 1 - - - - - - - - - - - - - - - - - - - - 0 ? ? ? ? 0 0 ? ? 1 ? ? 0 0 1 0 0 ? 0 0 0 2 2 ? ? ? ? ? ? ? 1 0 1 ? ? ? 0 9 1 ? ? ? ? ? ? ? ? ? ? ? ? ? ? ? ? 1 1 0 ? 1 ? 1 1 1 0 1 3 -

Bennettazhia_oregonensis ? ? ? ? ? ? ? ? ? ? ? ? ? ? ? ? ? ? ? ? ? ? ? ? ? ? ? ? ? ? ? ? ? ? ? ? ? ? ? ? ? ? ? ? ? ? ? ? ? ? ? ? ? ? ? ? ? ? ? ? ? ? ? ? ? ? ? ? ? ? ? ? ? ? ? ? ? ? ? ? ? ? ? ? ? ? ? ? ? ? ? ? ? ? ? ? ? ? ? ? ? ? ? ? ? ? ? ? ? ? ? ? ? ? ? ? ? ? ? ? ? ? ? ? ? ? ? ? ? 1 0 ? ? ? ? ? ? ? ? ? ? ? ? ? ? ? 1 1 0 0 1 0 1 0 8 1 1 ? ? ? ? ? ? ? ? ? ? ? ? ? ? ? ? ? ? ? ? ? ? ? ? ? ? ? ?

Dsungaripterus_weii 1 3 0 0 0 0 0 - 0 1 1 - - 1 0 0 1 2 1 0 - 1 1 1 2 3 3 0 1 0 1 - - - 1 - - - - 1 1 2 0 0 0 1 2 2 0 1 2 2 0 1 0 1 0 1 1 0 - 1 0 1 1 0 1 1 0 1 0 0 0 1 1 1 1 0 2 0 0 0 - 1 0 0 0 1 - 1 0 1 0 0 - 1 0 0 - 1 0 0 0 3 0 1 0 1 0 1 1 - - 1 2 0 1 0 1 0 1 1 0 0 1 0 1 0 1 1 1 1 0 0 1 0 0 1 1 2 1 1 1 0 0 1 ? ? 0 ? 0 ? 1 0 8 1 1 1 1 1 ? 0 ? 1 0 ? 1 0 0 1 1 2 1 3 0 ? 0 0 1 2 1 0 ? 3 -

Domeykodactylus_ceciliae ? ? ? ? 0 ? ? ? ? ? ? ? ? ? ? ? ? ? ? 0 ? ? 1 ? 2 ? ? ? 1 0 ? ? ? ? ? ? ? ? ? ? ? ? ? ? ? ? ? ? ? ? ? ? ? ? ? ? ? ? ? ? ? ? ? ? ? ? ? ? ? ? ? ? ? ? ? ? 1 1 ? 0 ? ? ? 1 0 ? 0 1 - ? ? ? ? ? ? ? 0 0 - 5 ? ? 0 3 0 ? ? ? 0 1 ? ? ? ? ? 0 1 ? ? ? ? ? ? ? ? ? ? ? ? ? ? ? ? ? ? ? ? ? ? ? ? ? ? ? ? ? ? ? ? ? ? ? ? ? ? ? ? ? ? ? ? ? ? ? ? ? ? ? ? ? ? ? ? ? ? ? ? ? ? ? ? ? ? ? ?

Noripterus_parvus 1 3 0 0 0 1 0 - 0 1 1 - - 1 0 0 1 2 0 0 - 1 1 1 1 3 3 0 1 0 1 - - - 1 - - - - 1 1 2 0 0 0 1 2 2 0 1 2 2 0 1 ? 1 0 1 1 0 - 1 0 1 1 0 1 ? 0 1 0 0 0 1 ? 1 1 1 2 0 0 0 - 1 0 0 0 1 - 1 0 1 0 0 - 1 0 0 - 4 0 0 0 3 0 1 0 1 0 1 1 - - 1 1 0 1 0 1 0 1 0 0 0 1 0 1 0 1 1 1 ? ? ? ? 0 0 ? 1 2 ? ? ? 0 0 ? 0 0 0 1 0 0 1 0 8 1 1 1 1 1 0 0 4 1 ? 1 1 0 0 ? 0 2 1 ? ? ? 0 0 1 2 1 0 ? ? ?

Noripterus_complicidens ? ? ? ? 0 ? ? - ? ? ? ? ? ? ? ? ? ? ? ? ? ? ? ? ? ? ? ? ? ? ? ? ? ? ? ? ? ? ? ? ? ? ? ? ? ? ? ? ? ? ? ? ? ? ? ? ? ? ? ? ? ? ? ? ? ? ? ? ? ? ? ? ? ? ? ? 1 ? ? 0 0 0 - 1 0 0 0 ? ? 1 0 ? ? 0 - ? 0 0 - 4 0 ? 0 3 0 ? ? 1 ? 1 1 - - ? ? ? ? ? ? 0 1 1 0 0 1 0 1 ? ? 1 ? 1 0 0 ? ? ? ? 1 2 ? ? ? 0 0 ? 0 1 0 1 1 0 1 0 8 1 1 1 1 1 0 0 ? ? 0 1 1 0 0 ? 0 2 1 1 0 1 0 0 1 2 1 0 ? 3 -

Tupuxuara_longicristatus ? ? ? 0 0 ? 0 - 0 - ? - - 1 0 ? ? ? ? 0 - 1 1 0 0 5 ? 0 1 2 ? - - - ? ? ? ? ? ? ? ? ? ? ? ? ? ? ? 1 ? ? ? ? 0 ? 0 ? ? ? - ? ? ? ? ? ? ? 1 1 1 1 0 ? ? ? ? ? ? ? ? ? ? ? ? ? ? ? ? ? ? ? ? ? ? ? 1 - - - - - - - - - - - - - - - - - - - - 0 ? ? ? ? ? ? ? 0 ? ? ? ? ? ? ? ? ? ? ? ? ? ? ? ? ? ? 0 ? ? ? ? ? ? ? ? ? ? ? ? ? ? ? ? ? ? ? 2 1 ? 0 ? ? 1 ? ? ? ? ? ? ? ? ? ? ? ? ? ?

Tupuxuara_leonardii 1 3 0 0 0 1 0 - 0 - 1 - - 1 0 1 2 4 1 0 - 1 1 0 0 5 3 0 1 2 0 - - - 1 - - - - 1 1 0 0 0 0 1 2 2 0 1 2 3 0 1 0 0 0 1 1 0 - 1 0 1 1 0 1 1 1 1 1 1 0 1 1 1 1 1 2 0 0 0 - 1 0 0 0 1 - 0 0 1 0 0 - 1 1 - - - - - - - - - - - - - - - - - - - - 0 1 0 1 1 0 0 1 0 1 ? ? 1 1 ? ? ? ? 0 0 1 1 2 1 ? 1 0 0 1 1 0 ? ? 1 ? 1 0 8 1 1 1 1 1 0 0 4 ? ? ? 1 0 0 1 1 0 ? ? ? ? 1 1 1 2 1 0 1 ? ?

Thalassodromeus_sethi 1 3 0 0 0 1 0 - 0 - 1 - - 1 0 1 2 4 1 0 - 1 1 0 0 5 3 0 1 2 0 - - - 0 1 0 0 0 1 1 0 0 0 0 1 2 2 0 1 2 3 0 1 0 0 0 1 1 0 - 1 0 ? 1 ? 1 1 0 1 1 1 0 1 ? 1 1 1 ? 0 0 0 - 1 0 0 0 1 - 1 2 1 0 0 - 1 1 - - - - - - - - - - - - - - - - - - - - 0 ? ? ? ? ? ? ? ? ? ? ? ? ? ? ? ? ? ? ? ? ? ? ? ? ? ? ? ? ? ? ? ? ? ? ? ? ? ? ? ? ? ? ? ? ? ? ? ? ? ? ? ? ? ? ? ? ? ? ? ? ? ? ? ? ? ? ?

Chaoyangopterus_zhangi 2 3 0 0 0 1 0 - 0 - ? - - 1 ? ? ? ? ? 1 - ? 0 - - - - - - - 0 - - - ? ? ? ? ? ? ? ? ? ? ? ? ? ? ? 1 ? ? ? ? ? ? ? ? ? ? - ? ? ? ? ? ? ? ? ? ? ? ? ? ? ? 1 1 2 0 0 0 - 1 1 0 0 1 - ? ? ? ? 0 - ? 1 - - - - - - - - - - - - - - - - - - - - 0 ? 0 0 ? 1 2 1 1 1 ? ? ? ? ? ? ? ? 0 0 ? ? 2 1 1 1 0 0 ? ? 0 0 1 ? ? 1 0 ? ? ? 1 ? 1 ? ? 4 ? 1 1 1 0 0 ? ? 0 1 2 0 1 1 ? 1 2 1 0 1 3 ?

Jidapterus_edentus 2 3 0 0 0 1 0 - 0 - 1 - - 1 ? ? ? ? ? 1 - ? 0 - - - - - - - 0 - - - ? ? ? ? ? ? ? ? ? ? ? ? ? ? ? 1 ? ? ? 1 0 ? ? ? ? ? - 1 ? ? ? ? ? ? 0 0 ? ? ? ? ? ? 1 1 2 0 0 0 - 1 1 0 0 1 - ? ? 1 0 0 - 1 1 - - - - - - - - - - - - - - - - - - - - 0 ? 0 0 ? 1 2 1 1 1 ? ? ? ? ? ? ? ? ? 0 ? ? 2 1 ? 1 0 0 1 ? ? 0 1 ? 0 ? ? ? ? ? ? 1 1 0 ? 4 ? 1 1 1 0 0 1 1 ? ? 3 ? 1 1 1 1 2 1 ? ? 3 -

Eoazhdarcho_liaoxiensis ? ? ? ? 0 ? ? - ? ? ? ? ? ? ? ? ? ? ? ? ? ? ? ? ? ? ? ? ? ? ? ? ? ? ? ? ? ? ? ? ? ? ? ? ? ? ? ? ? ? ? ? 0 ? ? ? ? ? ? ? ? ? ? ? ? ? ? ? ? ? - - ? ? ? ? ? 1 2 0 0 0 - 1 1 ? 0 1 - 0 0 1 0 0 - 1 1 - - - - - - - - - - - - - - - - - - - - ? ? 0 0 ? 1 2 ? 1 1 ? ? 0 ? ? ? ? ? ? 0 ? 2 2 ? ? ? 0 0 ? ? ? 0 1 1 ? ? 0 8 ? ? 1 1 1 ? ? 4 ? 2 1 1 0 ? ? 1 0 ? ? ? ? 1 ? ? ? 1 ? ? ? ?

Shenzhoupterus_chaoyangensis 2 3 0 0 ? 1 0 - 0 - 1 - - 1 0 1 2 ? 1 1 - 1 0 - - - - - - - 0 - - - 1 - - - ? 1 1 0 1 ? 0 1 3 0 1 ? 2 3 0 1 ? 0 0 0 2 0 ? 1 ? ? ? ? 1 ? ? ? ? ? ? ? ? ? 1 1 2 0 0 0 - 1 1 0 0 1 - ? ? 1 ? 0 - ? 1 - - - - - - - - - - - - - - - - - - - - 0 ? ? ? ? 1 2 ? 1 1 ? 1 0 ? ? ? ? ? 0 0 ? 2 2 ? ? ? ? ? ? ? ? 0 1 ? ? 1 0 8 ? ? ? ? ? ? 0 ? ? 2 1 ? 0 ? 1 ? 0 ? ? ? ? 1 ? 1 ? ? 0 1 3 -

Zhejiangopterus_linhaiensis 0 3 0 0 ? 1 0 - 0 - ? - - 1 0 1 2 4 1 0 - 1 0 - - - - - - - 0 - - - 1 - - - - 0 - - ? ? 0 0 - 0 1 1 2 3 0 1 0 0 1 0 1 0 - 1 1 ? ? ? 1 ? ? ? ? ? ? ? ? ? 1 1 2 0 0 0 - 1 1 0 0 1 - ? ? 1 0 0 - ? 1 - - - - - - - - - - - - - - - - - - - - 0 1 ? ? ? 2 2 ? 1 1 0 ? 1 1 1 0 0 ? 0 0 ? 2 2 ? ? 1 ? ? 1 ? ? 0 1 ? ? 1 1 8 ? ? 1 1 ? ? ? 4 ? 2 ? 1 0 ? 1 ? 3 1 1 0 1 1 ? 1 2 1 0 ? ? ?

Azhdarcho_lancicollis 1 3 0 0 0 1 0 - ? - ? - - 1 ? ? ? ? ? 0 - 1 0 - - - - - - - ? - - - ? ? ? ? ? ? ? ? ? ? ? ? ? ? ? ? ? ? 0 ? ? ? ? ? ? ? ? ? ? ? ? ? ? ? 0 0 - - ? ? ? 1 1 1 2 0 0 0 - 1 ? 0 ? ? ? 0 0 1 ? 0 - ? 1 - - - - - - - - - - - - - - - - - - - - 0 1 0 0 1 2 2 1 1 1 ? 1 1 ? ? ? ? 1 0 0 1 2 2 1 0 1 0 0 ? 1 0 0 1 1 1 1 1 8 1 ? 1 1 1 0 0 ? ? ? ? ? 0 0 ? 1 3 ? ? ? ? 1 1 1 2 1 1 ? ? ?

Arambourgiania_philadelphiae ? ? ? ? ? ? ? ? ? ? ? ? ? ? ? ? ? ? ? ? ? ? ? ? ? ? ? ? ? ? ? ? ? ? ? ? ? ? ? ? ? ? ? ? ? ? ? ? ? ? ? ? ? ? ? ? ? ? ? ? ? ? ? ? ? ? ? ? ? ? ? ? ? ? ? ? ? ? ? ? ? ? ? ? ? ? ? ? ? ? ? ? ? ? ? ? ? ? ? ? ? ? ? ? ? ? ? ? ? ? ? ? ? ? ? ? ? ? ? 0 0 1 2 2 1 1 ? ? ? ? ? ? ? ? ? ? ? ? ? ? ? ? ? ? ? ? ? ? ? ? ? ? ? ? ? ? ? ? ? ? ? ? ? ? ? ? ? ? ? ? 1 ? ? ? ? ? ? ? ? ? ? ? ? ? ?

Quetzalcoatlus_northropi 0 3 0 0 0 1 0 - 0 - 1 - - 1 0 1 2 ? ? 0 - 1 1 - 1 6 [23] 0 1 1 0 - - - 1 - - - - ? ? ? 1 0 0 ? ? ? ? 1 2 3 0 1 ? 0 1 0 1 0 - 1 ? ? ? ? ? ? 0 0 - - 0 1 ? 1 1 1 2 0 0 0 - 1 1 0 0 1 - 0 0 1 0 0 - 1 1 - - - - - - - - - - - - - - - - - - - - 0 1 0 0 1 2 2 1 1 1 0 1 1 1 ? ? ? 1 0 0 1 2 2 1 1 1 0 0 ? 1 0 0 1 1 1 1 1 8 1 1 1 1 1 0 0 4 1 2 ? 1 0 0 ? 1 3 ? ? ? 1 1 1 1 2 1 0 1 3 -

Liaodactylus_primus 2 1 ? 0 0 1 0 ? 0 1 1 ? ? 1 0 ? ? 4 1 0 ? ? ? ? ? ? ? ? ? ? 0 ? ? ? ? ? ? ? ? 0 ? ? ? ? ? 0 ? 1 1 1 3 2 0 1 0 ? 0 0 1 0 ? 1 ? ? 1 0 0 ? ? 1 ? 0 1 0 ? 1 1 1 0 0 0 0 ? 0 0 0 0 1 ? 1 ? 0 2 0 ? 1 0 0 ? 2 0 1 1 3 0 1 0 1 2 0 1 ? ? 0 0 0 0 0 1 ? ? ? ? ? ? ? ? ? ? ? ? ? ? ? ? ? ? ? ? ? ? ? ? ? ? ? ? ? ? ? ? ? ? ? ? ? ? ? ? ? ? ? ? ? ? ? ? ? ? ? ? ? ? ? ? ? ? ? ? ? ? ? ? ? ?

;

ccode + 39 44 59 63 65 76 80 89.90 97 116 130 142 144 147 150.151 161 178 203 212 218 222.223;

ccode - 40.43 45.58 60.62 64 66.75 77.79 81.88 91.96 98.115 117.129 131.141 143 145.146 148.149 152.160 162.177 179.202 204.211 213.217 219.221;

cnames

{0 Skull_aspect_ratio,_length_with_maximum_at_squamosal_relative_to_height_with_maximum_at_jaw_articulation_exclusive_of_cranial_crests: continuous,_values_from_1.664_to_12.438_scaled_from_0_to_1;

{1 Skull,_length_to_squamosal_relative_to_dorsal_vertebra_length: continuous,_values_from_6.348_to_59.164_scaled_from_0_to_1;

{2 Mandble,_length_relative_to_skull_length_to_squamosal: continuous,_values_from_0.603_to_1.152_scaled_from_0_to_1;

{3 Rostrum,_length_to_external_naris_(or_nasoantorbital_fenestra)_relative_to_skull_length_to_squamosal: continuous,_values_from_0.021_to_0.888_scaled_from_0_to_1;

{4 External_naris,_length_relative_to_skull_length_to_squamosal: continuous,_values_from_0.056_to_0.391_scaled_from_0_to_1;

{5 External_naris,_length_relative_to_height: continuous,_values_from_0.679_to_9.444_scaled_from_0_to_1;

{6 Antorbital_fenestra,_length_relative_to_skull_length_to_squamosal: continuous,_values_from_0.093_to_0.333_scaled_from_0_to_1;

{7 Antorbital_fenestra,_length_relative_to_height: continuous,_values_from_0.884_to_2.646_scaled_from_0_to_1;

{8 Nasoantorbital_fenestra,_length_relative_to_skull_length_to_squamosal: continuous,_values_from_0.126_to_0.846_scaled_from_0_to_1;

{9 Nasoantorbital_fenestra,_length_relative_to_height: continuous,_values_from_0.859_to_9.706_scaled_from_0_to_1;

{10 Orbit,_length_relative_to_height: continuous,_values_from_0.429_to_1.838_scaled_from_0_to_1;

{11 Supratemporal_fenestra,_length_relative_to_skull_length_to_squamosal: continuous,_values_from_0.026_to_0.235_scaled_from_0_to_1;

{12 Subtemporal_fenestra,_length_relative_to_width: continuous,_values_from_1.120_to_7.061_scaled_from_0_to_1;

{13 Rostral_tooth_row,_length_relative_to_skull_length: continuous,_values_from_0.051_to_0.807_scaled_from_0_to_1;

{14 Teeth,_maximum_number_divided_by_1000: continuous;

{15 Mandibular_symphysis,_length_relative_to_mandible_length: continuous,_values_from_0.052_to_0.688_scaled_from_0_to_1;

{16 Mandibular_tooth_row,_length_relative_mandible_length: continuous,_values_from_0.228_to_0.884_scaled_from_0_to_1;

{17 Mid-cervical_vertebra,_maximum_length_relative_to_mid-width: continuous,_values_from_1.011_to_15.918_scaled_from_0_to_1;

{18 Mid-cervical_vertebra,_maximum_length_relative_to_dorsal_vertebra_length: continuous,_values_from_0.523_to_22.419_scaled_from_0_to_1;

{19 Dorsal_vertebra,_length_relative_to_maximum_diameter: continuous,_values_from_0.928_to_2.333_scaled_from_0_to_1;

{20 Caudal_vertebra,_length_relative_to_dorsal_vertebra_length: continuous,_values_from_0.355_to_7.188_scaled_from_0_to_1;

{21 Caudal_vertebra,_length_relative_to_diameter: continuous,_values_from_0.747_to_9.932_scaled_from_0_to_1;

{22 Scapula,_length_relative_to_coracoid_length: continuous,_values_from_0.740_to_2.750_scaled_from_0_to_1;

{23 Radius,_diameter_relative_to_ulna_diameter: continuous,_values_from_0.364_to_1.171_scaled_from_0_to_1;

{24 Humerus,_length_relative_to_dorsal_vertebra_length: continuous,_values_from_3.706_to_18.426_scaled_from_0_to_1;

{25 Ulna,_length_relative_to_humerus_length: continuous,_values_from_0.842_to_1.919_scaled_from_0_to_1;

{26 Pteroid,_length_relative_to_ulna_length: continuous,_values_from_0.111_to_0.784_scaled_from_0_to_1;

{27 Metacarpal_IV,_length_relative_to_humerus_length: continuous,_values_from_0.128_to_2.996_scaled_from_0_to_1;

{28 Metacarpal_IV_mid-width_relative_to_combined_ulna_and_radius_mid-width: continuous,_values_from_0.175_to_0.948_scaled_from_0_to_1;

{29 Metacarpal_IV_proximal_end,_dorsoventral_width_relative_to_mid-width: continuous,_values_from_1.250_to_2.975_scaled_from_0_to_1;

{30 Manual_digit_IV_first_phalanx,_length_relative_to_humerus_length: continuous,_values_from_0.057_to_3.523_scaled_from_0_to_1;

{31 Manual_digit_IV_second_phalanx,_length_relative_to_first_phalanx_length: continuous,_values_from_0.502_to_1.575_scaled_from_0_to_1;

{32 Manual_digit_IV_third_wing_phalanx,_length_relative_to_first_phalanx_length: continuous,_values_from_0.285_to_1.317_scaled_from_0_to_1;

{33 Manual_digit_IV_fourth_wing_phalanx,_length_relative_to_first_phalanx_length: continuous,_values_from_0.178_to_1.287_scaled_from_0_to_1;

{34 Ilium_anterior_process,_length_relative_to_posterior_process_length: continuous,_values_from_0.183_to_3.640_scaled_from_0_to_1;

{35 Femur,_length_relative_to_humerus_length: continuous,_values_from_0.628_to_1.971_scaled_from_0_to_1;

{36 Tibia,_length_relative_to_femur_length: continuous,_values_from_0.839_to_1.993_scaled_from_0_to_1;

{37 Fibula,_free_length_relative_to_tibia_length: continuous,_values_from_0.156_to_1.005_scaled_from_0_to_1;

{38 Metatarsal_III,_length_relative_to_tibia_length: continuous,_values_from_0.110_to_0.554_scaled_from_0_to_1;

{39 Skull_dorsal_margin,_curvature_exclusive_of_cranial_crests: ordered convex straight concave;

{40 Rostrum_anterior_margin,_shape: flat_surface blunt sharp_tip rostral;

{41 Rostral_process_cross-section,_shape: triangular elliptical;

{42 Rostrum,_shape: compressed_laterally shortened_anteroposteriorly depressed_dorsoventrally;

{43 Jaws,_lateral_taper: attenuated subparallel wide;

{44 Rostrum_anterior_end,_orientation: ordered upturned straight downturned;

{45 Rostrum_anterior_expansion: absent present;

{46 Skull_anterior_expansion,_horizontal_outline: elliptical triangular quadrangular;

{47 External_naris_(or_nasoantorbital_fenestra)_dorsal_and_ventral_margins,_orientation: acute_angle subparallel;

{48 External_naris_(or_nasoantorbital_fenestra)_anterior_margin,_position_relative_to_premaxillary_toothrow: dorsal posterior;

{49 Antorbital_(or_nasoantorbital)_fossa_on_jugal: present absent;

{50 Antorbital_fenestra_dorsal_and_ventral_margins,_orientation: subparallel acute_angle;

{51Antorbital_fenestra_ventral_margin,_position_relative_to_external_naris_ventral_margin: same_level ventral;

{52 External_naris_and_antorbital_fenestra,_configuration: separate confluent;

{53 Antorbital_(or_nasoantorbital_fenestra)_posterior_margin,_shape: nearly_straight_angle rounded_outline;

{54 Orbit_outline,_shape: subcircular piriform inverted_triangle;

{55 Orbit,_dorsal_position: middle_of_the_skull_with_the_ventral_margin_of_the_orbit_below_the_middle_of_the_antorbital_(or_nasoantorbital)_fenestra_and_the_dorsal_margin_of_the_orbit_above_the_dorsal_margin_of_the_antorbital_(or_nasoantorbital)_fenestra high_in_the_skull_with_the_ventral_margin_of_the_orbit_the_same_level_or_above_the_middle_of_the_antorbital_(or_nasoantorbital)_fenestra low_in_the_skull_with_the_entire_orbit_lower_than_the_dorsal_margin_of_the_antorbital_(or_nasoantorbital)_fenestra;

{56 Infratemporal_fenestra,_shape: trapezoidal inverted_triangle upright_triangle oval elliptical;

{57 Infratemporal_fenestra,_position_relative_to_orbit: entire_infratemporal_fenestra_posterior_to_orbit infratemporal_fenestra_reaches_under_posterior_margin_of_orbit;

{58 Premaxillary_bar_(internasal_process),_width: wide narrow;

{59 Premaxilla_maxillary_process,_position: ordered contacts_nasal reaches_posterior_half_of_external_naris anterior_to_middle_of_external_naris;

{60 Premaxilla_(internasal_process)_posterior_margin,_position: terminate_between_nasals contacts_frontals;

{61 Premaxillary_crest: absent present;

{62 Premaxillary_crest_anterior_margin,_position_relative_to_skull_anterior_margin: level posterior;

{63 Premaxillary_crest_anterior_margin,_orientation: ordered inclined_posteriorly vertical curving_anterodorsally;

{64 Premaxillary_crest,_shape: tall_triangle_decreasing_in_height_posteriorly low_blade low_with_anterior_humped_margin comb-like_with_straight_dorsal_margin round_dorsal_margin tall_triangle_increasing_in_height_posteriorly rectangular;

{65 Premaxillary_crest,_position: ordered anterior_to_external_naris_(or_nasoantorbital_fenestra)_anterior_margin between_external_naris_(or_nasoantorbital_fenestra)_anterior_margin_and_orbit above_orbit above_occipital_region;

{66 Premaxillary_crest_dorsal_spine: absent present;

{67 Premaxillary_crest,_thickness: single_plate two_plates_separated_by_trabeculae;

{68 Premaxillary_crest,_texture: striated smooth branching_system_of_grooves;

{69 Maxilla_posterior_end,_shape: narrow ventrally_expanded;

{70 Maxilla_ascending_process,_shape: broad tapered slender;

{71 Maxilla_and_nasal_contact,_position: maxilla_contacts_main_body_of_nasal maxilla_contacts_only_descending_process_of_nasal;

{72 Maxilla_premaxillary_and_jugal_processes,_shape: jugal_process_wider both_narrow premaxillary_process_wider both_wide;

{73 Nasal_descending_process: present absent;

{74 Nasal_descending_process,_position: lateral medial;

{75 Nasal_descending_process,_length: short elongate;

{76 Nasal_descending_process,_orientation: ordered inclined_anteriorly ventral inclined_posteriorly;

{77 Nasal_process,_lateral_pneumatic_foramen: absent present;

{78 Frontal_crest: absent present;

{79 Frontal_crest,_shape: low_and_blunt low_and_elongated high_and_expanded;

{80 Frontal_crest_anterior_margin,_position: anterior_to_orbit above_orbit posterior_to_orbit;

{81 Frontal_anterior_margin,_position_relative_to_preorbital_bar_anterior_margin: anterior posterior;

{82 Lacrimal_foramen: absemt present;

{83 Lacrimal_descending_process_posterior_margin,_shape: flat orbital_process;

{84 Parietal_crest: absent present;

{85 Parietal_crest,_shape: blunt expanded_into_rounded_margin tapered_into_triangular_process elongate_process;

{86 Squamosal,_shape: unexpanded rounded expanded;

{87 Squamosal,_position: above_or_level_with_base_of_lacrimal_process_of_jugal entirely_below_base_of_lacrimal_process_of_jugal;

{88 Jugal_posterior_process: present absent;

{89 Quadrate,_inclination_relative_to_ventral_margin_of_skull: ordered anteriorly subvertical 120˚_posteriorly 150˚_posteriorly;

{90 Mandibular_articulation,_position_relative_to_center_of_orbit: ordered posterior_to_orbit posterior_to_center_below_orbit underneath_center_below_orbit anterior_to_center_below_orbit anterior_to_orbit;

{91 Quadrate,_shape: wide thin_and_cylindrical;

{92 Jugal_ventral_margin,_shape: straight concave;

{93 Jugal_anterior_margin,_position: does_not_reach_anterior_margin_of_the_nasoantorbital_fenestra reaches_anterior_margin_of_the_nasoantorbital_fenestra;

{94 Jugal_postorbital_process_and_lacrimal,_configuration: do_not_contact contact_to_form_lower_orbital_bar;

{95 Jugal_ascending_and_postorbital_processes,_shape: separated_by_distinct_angle infilled_by_concave_flange;

{96 Jugal_ascending_process_base,_width: broad narrow;

{97 Jugal_ascending_process,_inclination: ordered anterodorsal vertical posterodorsal;

{98 Jugal_postorbital_process_anterior_margin,_shape: flat orbital_process;

{99 Jugal_posterior_process,_orientation: posterior ventral;

{100 Jugal_maxillary_process: absent present;

{101 Occiput,_orientation: posteroventral ventral;

{102 Basioccipital,_length_relative_to_width: shorter_than_wide longer_than_wide;

{103 Basisphenoid_body,_length: shorter_than_wide at_least_longer_than_wide;

{104 Elongate_basipterygoid_processes: absent present;

{105 Supraoccipital_crest: absent present;

{106 Supraoccipital,_pneumatic_foraminae: absent present;

{107 Palate,_posterior_end,_shape: concave convex;

{108 Palatal_ridge: absent present;

{109 Palatal_ridge,_position: tapering_anteriorly confined_posteriorly;

{110 Palatal_ridge_shape: narrow_strip strong_keel;

{111 Palatines,_shape: broad_and_flat thin_bars;

{112 Internal_nares_and_maxilla,_configuration: contact do_not_contact;

{113 Interpterygoid_opening,_length_relative_to_subtemporal_fenestra_length: at_least_subtemporal_fenestra shorter_than_subtemporal_fenestra;

{114 Mandible_articulation_condyles,_orientation: parasagittal oblique;

{115 Nutrient_foramina_positioned_in_a_row_along_the_lateral_margin_of_the_jaws: present absent;

{116 Mandible_anterior_end,_orientation: ordered upturned straight downturned;

{117 Mandible_anterior_margin,_shape: blunt prow sharp_tip;

{118 Mandible,_shape: compressed_laterally shortened_anteroposteriorly depressed_dorsoventrally;

{119 Mandible_anterior_end,_expansion: absent present;

{120 Mandible_anterior_end_dorsal_margin,_shape: level distinct_eminence;

{121 Mandible_anterior_end_distinct_eminence,_height: low high;

{122 Mandibular_symphysis,_fusion: unfused fused;

{123 Mandibular_symphysis_ventral_margin,_position: subparallel_to_rami oblique_to_rami;

{124 Mandible_anterior_end_lateral_surfaces,_texture: flat large_foramina pitted;

{125 Mandible_mid-depth,_relative_to_length: at_most_one-ninth_the_length more_than_one-ninth_the_length;

{126 Mandibular_rami_distinct_dorsal_eminence: present absent;

{127 Mandibular_rami_dorsal_eminence,_shape: rounded pointed;

{128 Mandibular_sulcus: absent present;

{129 Mandible_symphysis_anterior_end,_shape: flat_or_concave fossa keel;

{130 Retroarticular_process,_orientation_relative_to_mandible: ordered posteroventral subhorizontal posterodorsal;

{131 Retroarticular_process,_shape: triangular subcircular elongate blunt;

{132 Mandibular_crest: absent present;

{133 Mandibular_crest,_shape: blade_like_and_low massive_and_deep;

{134 Dentary,_length: do_not_separate_angular_and_surangular separate_angular_and_surangular;

{135 Teeth: present absent;

{136 Teeth,_variation_in_shape_along_tooth_row: isodont heterodont;

{137 Mesial_heterodont_teeth,_shape: recurved_triangle slender_needle recurved_spike;

{138 Cheek_teeth,_shape: recurved_triangle bulbous_triangle slender_needle recurved_cone labiolingually_compressed_triangle recurved_spike;

{139 Teeth,_texture: smooth striated sharp_mesial_and_distal_keels medial_carinae;

{140 Teeth,_maximum_crown_height_relative_to_basal_width: less_than_four_times_width at_least_four_times_width;

{141 Teeth,_lateral_orientation: vertical inclined_laterally;

{142 Teeth,_average_spacing_between_successive_teeth: ordered nearly_touching less_than_teeth_widths subequal_to_teeth_widths more_than_teeth_widths;

{143 Teeth,_size_variation: transition_along_tooth_row sharp_disparity_in_size_between_mesial_and_distal_teeth;

{144 Upper_dentition,_size_relative_to_lower_dentition: ordered upper_dentition_significantly_larger upper_dentition_subequal_or_slightly_larger upper_dentition_significantly_smaller;

{145 Teeth,_maximum_curvature: displacement_of_curvature_less_than_tooth_diameter displacement_of_curvature_at_least_tooth_diameter;

{146 Teeth,_curvature_orientation: posterior lingual;

{147 Teeth,_inclination: ordered upright mesial_teeth_procumbent procumbent;

{148 Jaw_lateral_margins,_shape: straight undulating;

{149 Cheek_teeth,_denticles: present absent;

{150 Teeth_largest_denticles,_shape: ordered serrations cuspules crenulations low_cusps tall_cusps;

{151 Teeth,_maximum_denticle_number: ordered more_than_50 between_six_and_49 five;

{152 Rostral_dentition_anterior_end,_position_relative_to_rostrum_tip: reaches_tip posterior;

{153 Maxillary_teeth,_position_of_largest_teeth: mesial middle distal;

{154 Fifth_and_sixth_teeth_distinctly_smaller_than_fourth_and_seventh: absent present;

{155 Mandibular_dentition_anterior_end,_position_relative_to_tip_of_rostrum: reaches_tip posterior;

{156 Rostrum_occlusal_margin,_orientation: horizontal_or_ventrally_reflected dorsally_reflected;

{157 Atlantoaxis,_fusion: unfused fused;

{158 Mid-cervical_vertebra_neural_arch_lateral_surface,_pneumatic_foramen: absent present;

{159 Mid-cervical_vertebra_centrum_lateral_surface,_pneumatic_foramen: absent present;

{160 Mid-cervical_vertebra_lateral_to_neural_canal,_pneumatic_foramina: absent present;

{161 Mid-cervical_vertebra_neural_spines,_height: ordered tall low extremely_reduced;

{162 Mid-cervical_vertebrae_neural_spines,_lateral_outline_shape: blade triangular ridge;

{163 Mid-cervical_vertebra,_postexapophyses: absent present;

{164 Mid-cervical_vertebra_neural_arch_and_centrum,_configuration: distinct continuous;

{165 Mid-cervical_vertebra_ribs,_shape: elongate reduced;

{166 Cervical_8_neural_spine,_height: tall low;

{167 Cervical_9,_shape: similar_to_dorsal_vertebrae similar_to_cervical_8;

{168 Notarium: absent present;

{169 Anterior_dorsal_vertebra_neural_spines, shape: unfused;

{170 Caudal_vertebra,_number: more_than_15 at_most_15;

{171 Caudal_vertebra_zygapophyses,_length: short extremely_elongate;

{172 Caudal_vertebra_centrum,_shape: single duplex;

{173 Scapulocoracoid,_orientation_relative_to_vertebral_column: subparallel rotated_laterally;

{174 Scapula_posterior_end,_shape: elongate_and_laterally_compressed suboval_and_expanded;

{175 Scapula,_shape: elongate stout_with_constricted_shaft;

{176 Scapula_articulates_with_vertebral_column: absent present;

{177 Coracoid_ventral_margin,_shape: flat broad_tubercle deep_flange;

{178 Coracoid,_shape: ordered semicircular broad_shaft narrow_shaft;

{179 Sternocoracoid_articulations,_position_with_respect_to_one_another: lateral anterior_and_posterior;

{180 Sternum,_constriction_posterior_to_cristospine: present absent;

{181 Cristopine,_shape: shallow_and_elongated deep_and_short;

{182 Coracoid_sternal_articulation,_shape: flattened oval;

{183 Coracoid_sternal_articulation,_posterior_expansion: absent present;

{184 Sternum,_shape: narrow quadrangular semicircular triangular;

{185 Humerus_proximal_end_ventral_surface,_pneumatic_foramen: absent present;

{186 Humerus_proximal_end_dorsal_surface,_pneumatic_foramen: absent present;

{187 Humerus_shaft,_shape: straight bowed;

{188 Humerus_mid-shaft,_constriction: present absent;

{189 Humerus_distal_end_anterior_aspect,_pneumatic_foramen: absent present;

{190 Humerus_distal_aspect,_pneumatic_foramen: absent present;

{191 Humerus_distal_aspect,_shape: hourglass crescentic_or_D-shape subtriangular;

{192 Deltopectoral_crest,_position_on_humerus: placed_proximally placed_more_distally_on_shaft;

{193 Humerus_deltopectoral_crest,_shape: subtriangular_with_proximal_apex proximally_leaning_trapezoid proximally_curving_hook oblong_process_with_constricted_neck low_and_rectangular elongate_and_proximally_expanded hatchet-shape warped tall_rectangular_process knife-shape;

{194 Ulnar_crest_of_humerus,_size: reduced distinct;

{195 Ulnar_crest_of_humerus,_shape: narrow_and_directed_posteriorly massive_with_a_developed_proximal_ridge;

{196 Ulna_shaft_anterior_surface: shape: flat longitudinal_ridge;

{197 Ulna_distal_tuberculum,_position: middle_of_the_distal_end ventral_part_of_the_distal_end;

{198 Radius_distal_end_cross-section,_shape: suboval subtriangular_with_large_anterior_process;

{199 Distal_syncarpal_ventral_articular_facet_for_Metacarpal_IV,_size_relative_to_dorsal_facet: ventral_facet_larger subequal_in_size;

{200 Distal_syncarpal,_cross-section_shape: rectangular triangular;

{201 Pteroid,_shape: angled_at_midsection stout_hook straight_and_tapered_with_expanded_proximal_end straight_with_expanded_ends curved_slender_rod curved_and_not_tapered;

{202 Preaxial_carpal,_shape: longer_than_wide at_most_long_as_wide;

{203 Metacarpals,_number_articulating_with_carpus: ordered four_or_more two one;

{204 Metacarpals_I_to_III_distal_ends,_positions: disparate approximate;

{205 Metacarpal_IV_proximal_cross-section,_shape: anteroposteriorly_compressed subrectangular;

{206 Metacarpal_IV_shaft_cross-section,_shape: subrectangular anteroposteriorly_compressed;

{207 Metacarpal_distal_end_between_condyles,_shape: flat median_ridge;

{208 Manual_unguals,_size_relative_to_pedal_unguals: less_than_twice_the_size_of_pedal_unguals at_least_twice_the_size_of_pedal_unguals;

{209 Manual_digit_IV_first_phalanx_proximal_end_ventral_surface,_pneumatic_foramen: absent present;

{210 Manual_digit_IV_phalanges_shaft_cross-sections,_shape: round_to_subtriangular concave_posteriorly oval ventral_ridge;

{211 Ischium_ventral_margin,_shape: straight convex;

{212 Pubis_anterior_margin,_shape_in_lateral_view: ordered convex straight slightly_concave deeply_concave;

{213 Pubis_and_ischium_contact,_shape: confluent_along_length partially_separated_by_oval_opening;

{214 Prepubis,_length_relative_to_width: greater_than_width less_than_or_equal_to_width;

{215 Femur,_shape: strongly_bowed slight_curvature;

{216 Femur_proximal_end,_pneumatic_foramen: absent present;

{217 Femoral_neck,_shape: indistinct constricted;

{218 Greater_trochanter,_shape: ordered reduced distinct_process anteriorly-curved_hook;

{219 Femur_distal_end,_epicondyles_size: reduced_and_confluent_with_distal_condyles expanded_into_distinct_distal_flanges;

{220 Femoral_head,_orientation_relative_to_shaft: at_most_145° greater_than_145°;

{221 Metatarsal_IV,_length_relative_to_metatarsals_I_to_III: subequal significantly_shorter;

{222 Pedal_digit_V,_number_of_phalanges: ordered four three two at_most_one;

{223 Pedal_digit_V_second_phalanx,_shape: ordered straight curved bent_at_midsection;

;

agroup =0 (outgroup) 0.3;

agroup =1 (aspect) 4 11.12 20 25 32 38 42 44 53 60 74 79 81 93.94 97 108 111;

agroup =2 (nonaspect) 1.3 5.10 13.19 21.24 26.31 33.37 39.41 43 45.52 54.59 61.73 75.78 80 82.92 95.96 98.107 109.110;

hold 24000;

co auto;

mult =replic 2000 ratchet;

best;

proc/;
